# Genetic Analysis of Neurite Outgrowth Inhibitor‐Associated Genes in Parkinson's Disease: A Cross‐Sectional Cohort Study

**DOI:** 10.1111/cns.70070

**Published:** 2024-10-02

**Authors:** Xiurong Huang, Yige Wang, Yaqin Xiang, Yuwen Zhao, Hongxu Pan, Zhenhua Liu, Qian Xu, Qiying Sun, Jieqiong Tan, Xinxiang Yan, Jinchen Li, Beisha Tang, Jifeng Guo

**Affiliations:** ^1^ Department of Neurology, Xiangya Hospital Central South University Changsha Hunan China; ^2^ Department of Geriatrics, Xiangya Hospital Central South University Changsha Hunan China; ^3^ Centre for Medical Genetics & Hunan Key Laboratory of Medical Genetics, School of Life Sciences Central South University Changsha Hunan China; ^4^ National Clinical Research Center for Geriatric Disorders Xiangya Hospital, Central South University Changsha Hunan China; ^5^ Key Laboratory of Hunan Province in Neurodegenerative Disorders Central South University Changsha Hunan China; ^6^ Hunan International Scientific and Technological Cooperation Base of Neurodegenerative and Neurogenetic Diseases Changsha China

**Keywords:** axon growth, genotype–phenotype association, neurite outgrowth inhibitor‐associated genes, Parkinson's disease, rare variant

## Abstract

**Background:**

Parkinson's disease (PD) is a neurodegenerative disease caused by a combination of aging, environmental, and genetic factors. Previous research has implicated both causative and susceptibility genes in PD development. Nogo‐A, a neurite outgrowth inhibitor, has been shown to impact axon growth through ligand‐receptor interactions negatively, thereby involved in the deterioration of dopaminergic neurons. However, rare genetic studies have identified the relationship between neurite outgrowth inhibitor (Nogo)‐associated genes and PD from a signaling pathway perspective.

**Methods:**

We enrolled 3959 PD patients and 2931 healthy controls, categorized into two cohorts based on their family history and age at onset: sporadic early Parkinson's disease & familial Parkinson's disease (sEOPD & FPD) cohort and sporadic late Parkinson's disease (sLOPD) cohort. We selected 17 Nogo‐associated genes and stratified them into three groups via their function, respectively, ligand, receptors, and signaling pathway groups. Additionally, we conducted the burden analysis in rare variants, the logistic regression analysis in common variants, and the genotype–phenotype association analysis. Last, bioinformatics analysis and functional experiments were conducted to identify the role of the *MTOR* gene in PD.

**Results:**

Our findings demonstrated that the missense variants in the *MTOR* gene might increase PD risk, while the deleterious variants in the receptor subtype of Nogo‐associated genes might mitigate PD risk. However, common variants of Nogo‐associated genes showed no association with PD development in two cohorts. Furthermore, genotype–phenotype association analysis suggested that PD patients with *MTOR* gene variants exhibited relatively milder motor symptoms but were more susceptible developing dyskinesia. Additionally, bioinformatics analysis results showed *MTOR* gene was significantly decreased in PD, indicating a potential negative role of the mTOR in PD pathogenesis. Experimental data further demonstrated that MHY1485, a mTOR agonist, could rescue MPP^+^‐induced axon inhibition, further implicating the involvement of mTOR protein in PD by regulating cell growth and axon growth.

**Conclusions:**

Our preliminary investigation highlights the association of Nogo‐associated genes with PD onset in the Chinese mainland population and hints at the potential role of the *MTOR* gene in PD. Further research is warranted to elucidate the mechanistic pathways underlying these associations and their therapeutic implications.

## Graphical abstract

Here, we enrolled 3959 PD patients and 2931 healthy controls, categorized into two cohorts: the sporadic early Parkinson's disease & familial Parkinson's disease (sEOPD & FPD) cohort and the sporadic late Parkinson's disease (sLOPD) cohort, to identify the role of 17 Neurite Outgrowth Inhibitor‐associated genes. In specific, we conducted the burden analysis in rare variants, the Logistic regression analysis in common variants, and the genotype–phenotype association analysis. Last, Bioinformatics analysis and functional experiments were conducted to identify the role of the *MTOR* gene in PD. Our preliminary investigation highlights the association of Nogo‐associated genes with PD onset in the Chinese mainland population and elucidates the role of mTOR protein in PD via mediating axon growth.

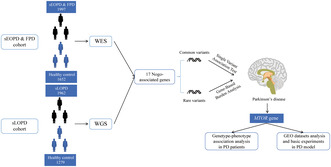

**Abbreviations**
AAO: age‐at‐onset
DEGs: differential expression genes
Dmis: damaging variant
EGFR: epithelial growth factor receptor
ER: endoplasmic reticulum
FDR: false discovery rate
FPD: familial Parkinson's disease
iPSC: induced pluripotent stem cells
LB: Lewy bodies
LoF: loss of function variant
MAF: minor allele frequency
MDS: movement disorder society
NgR: nogo receptor
Nogo: neurite outgrowth inhibitor
PD: Parkinson's disease
RBD: REM sleep behavior disorder
sEOPD: sporadic early Parkinson's disease
SKAT‐O: sequence kernel association test‐optimal
sLOPD: sporadic late Parkinson's disease
TrkB: tropomyosin receptor kinase B
WES: whole exome sequence
WGS: whole genome sequence

## 1 Background

Parkinson's disease (PD) is a common neurodegenerative disease characterized by symptoms such as resting tremor, rigidity, bradykinesia, postural gait abnormalities, and various nonmotor manifestations, including constipation, hyposmia, and REM sleep behavior disorder (RBD) [1]. Pathologically, PD is manifested by the loss of dopaminergic neurons and the presence of Lewy bodies (LB) in the substantia nigra [1, 2]. The interplay of aging, genetics, and environmental factors results in PD onset [3]. Additionally, previous studies have revealed that genetic factors play a greater role in early‐onset PD (EOPD) and familial PD (FPD). Since the *SNCA* gene was initially found, many pathogenic and susceptibility genes were identified to involve PD, such as *DJ‐1*, *LRRK2*, *PINK1*, and the *ATP13A2* genes [4, 5, 6]. However, a genetic study showed that despite identifying 23 pathogenic genes and the *GBA* gene, they just account for only a fraction of EOPD and FPD patients, indicating we still need to further explore other genetic factors [7].

Previous studies from our group demonstrated that the *NUS1* gene was a candidate gene for PD, with *NUS1* deficiency triggering increased apoptosis of dopaminergic neurons in *Drosophila* [8, 9, 10]. Notably, the *NUS1* gene expresses the specific neurite outgrowth inhibitor‐B (Nogo‐B) receptor, which is critical for protein N‐glycosylation, dolichol synthesis, and cholesterol metabolism [11, 12]. Nogo‐B is a member of the Nogo family, expressed by the *RTN4* gene. Additionally, the *RTN4* gene expresses three isoforms, including Nogo‐A, Nogo‐B, and Nogo‐C, which share the common C‐terminus and are located in the endoplasmic reticulum (ER) [13, 14]. Besides, the Nogo‐66 domain, presented in all isoforms, interacts with multiple receptors, while Nogo‐A specifically interacts with other receptors via its N‐terminus domain, Nogo‐Δ20, inhibiting axon growth and regeneration, which is primarily expressed in oligodendrocytes [13, 15, 16].

**FIGURE 1:**
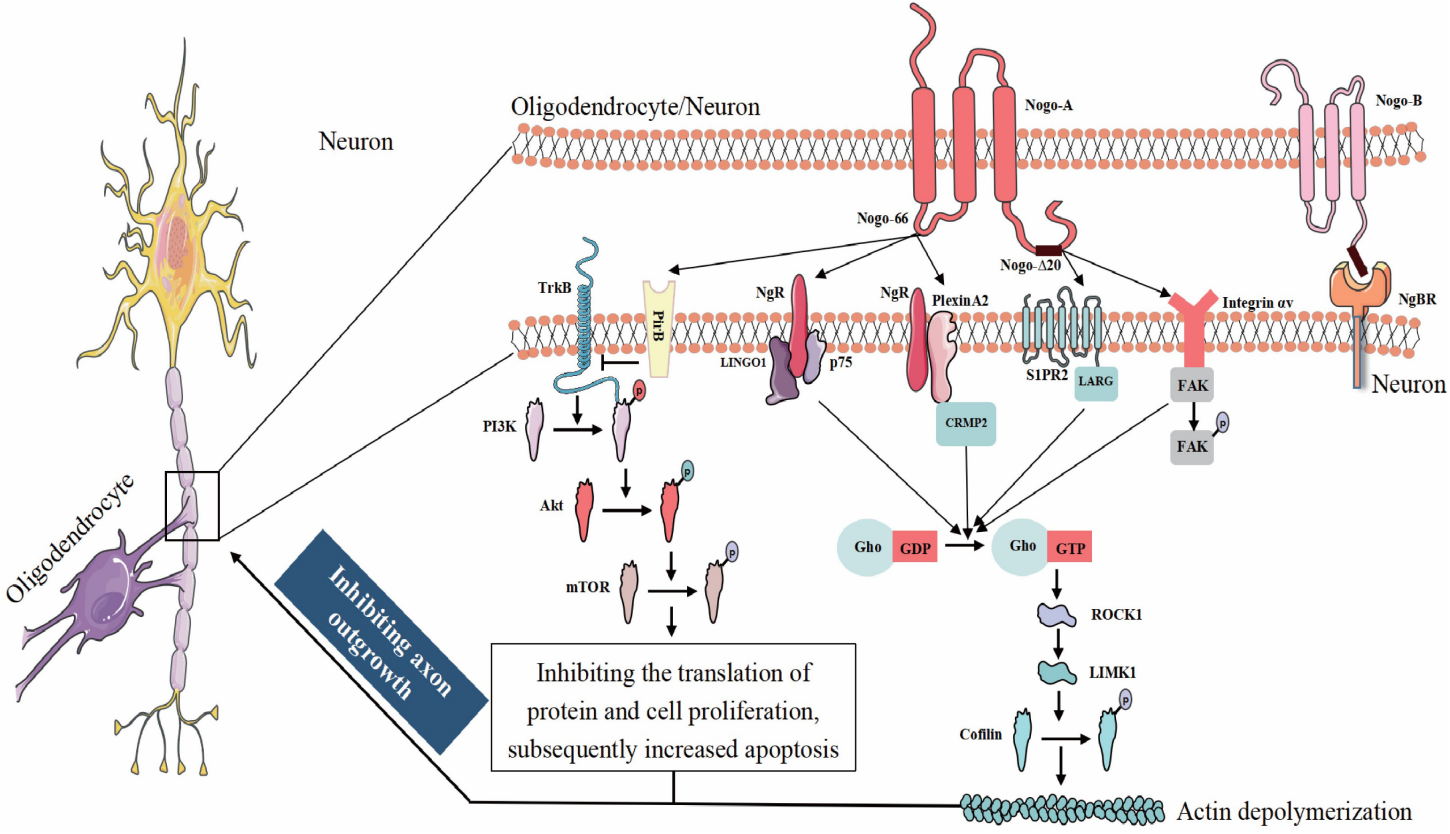
An overview of the Nogo‐related proteins and the pathways they are involved in. Nogo‐A protein can regulate the activity of the RhoA‐ROCK1‐LIMK1 signaling pathway through a variety of ligand‐receptor interactions, including NgR‐LINGO1, NgR‐PlexinA2‐CRMP2, Nogo‐A‐S1PR2, and Nogo‐A‐Integrin αv, which causes actin depolymerization and microtubule decomposition, thereby exerting the function of inhibiting axon growth. Additionally, the Nogo‐A protein can inhibit the activity of TrkB and its downstream PI3K‐Akt–mTOR signal pathway by binding to PirB, thereby restraining protein transcription, translation, and cell proliferation and inducing cell apoptosis.

Notably, Nogo‐A can contribute to actin depolymerization by interacting with many receptors and regulating the RhoA‐ROCK1‐LIMK1 pathway [17]. The Nogo‐66 domain can interact with the Nogo receptor (NgR) to activate the ROCK1‐LIMK1 pathway, LINGO1, p75, as well as PlexinA2, manifested as co‐receptors [18, 19, 20]. PlexinA2 can modulate the phosphorylation of CRMP2, subsequently promoting the activation of the ROCK1‐LIMK1 pathway [21, 22, 23, 24]. Moreover, another ligand, Nogo‐Δ20, interacts, respectively, with S1PR2 and Integrin αv to attune the RhoA‐ROCK1‐LIMK1 pathway [25, 26]. In addition, Nogo‐66 can inhibit the activity of Tropomyosin Receptor Kinase B (TrkB) via PirB, attenuating the PI3K‐AKT–mTOR pathway, thus inhibiting protein translation and cell proliferation [27, 28]. Collectively, Nogo‐A is involved in negatively regulating axon growth and regeneration, which suggests its potential role in PD pathogenesis, as illustrated in Figure 1.

Previous studies have indicated that Nogo‐A had a negative effect on dopaminergic neuron survival. For example, LINGO1 was upregulated in a PD animal model and interacted with the epithelial growth factor receptor (EGFR) to impede dopaminergic neuron survival [29]. On the contrary, blocking LINGO1 expression could prevent neuron death and abnormal behavior in animal models [30]. However, clinical studies in China and Germany failed to find an association between the *LINGO1* gene and PD risk [31, 32].

Given the conflicting results, it is critical to explore whether Nogo‐associated genes participate in PD pathogenesis. According to the age‐at‐onset (AAO) and family history, we divided all PD patients into two groups, respectively, the sporadic early Parkinson's disease and familial Parkinson's disease (sEOPD & FPD) cohort and the sporadic late Parkinson's disease (sLOPD) cohort. Specifically, those with an AAO of no more than 50 years old or with a family history were grouped into the sEOPD & FPD cohort, while sporadic late‐onset PD patients (AAO > 50 years and without family history) constituted the sLOPD cohort. Subjects in the sEOPD & FPD cohorts underwent whole exome sequencing (WES) to detect variants primarily in exonic regions to look for potential novel disease‐causing genes. In contrast, subjects in the sLOPD cohort underwent whole genome sequencing (WGS) to explore the risk loci, particularly focusing on intronic variants. Based on the above two cohorts, we aimed to systematically screen and identify Nogo‐associated genes, providing insights into their role in PD pathogenesis.

## 2 Methods

### 2.1 Participants

We recruited a total of 3959 PD patients and 2931 healthy controls. All PD patients were diagnosed by two professional physicians following either the United Kingdom Parkinson's Disease Society Brain Bank Diagnostic Criteria or Movement Disorder Society (MDS) Clinical Diagnostic Criteria for Parkinson's disease [33, 34]. The recruitment of PD patients was carried out in Xiangya Hospital and other collaborative hospitals, with enrollment in the Parkinson's Disease & Movement Disorders Multicenter Database and Collaborative Network in China (PD‐MDCNC). All healthy controls were excluded from neurological diseases and divided into two cohorts. The Xiangya Ethics Committee approved our study, and all participants provided informed consent.

### 2.2 Nogo‐Associated Genes Selection

A comprehensive search of the PubMed database was performed to screen Nogo‐associated genes, categorized into three groups based on their role in axon growth: ligand, receptor, and signal pathways. The specific genes involved were listed in Table S1. Additionally, a flowchart about how the study was designed and performed was exhibited in Figure S1.

### 2.3 Variants Selection

First, all these PD patients have excluded the pathogenic/likely pathogenic variants of 23 PD pathogenic genes. Then, we screened the rare variants of two cohorts and the common variants of the sEOPD & FPD cohort in the coding region of Nogo‐associated genes. In contrast, the common variants of the sLOPD cohort contained the whole genome region. According to the minor allele frequency (MAF), variants with MAF ≥ 0.01 were classified as common, while those with lower frequencies were considered rare. Meanwhile, rare variants were further subdivided into four groups, including synonymous variant, missense variant, loss of function variant (LoF), and damaging variant (Dmis), with Dmis being missense variants predicted as damaging via CADD (C‐score > 12.37) and LoF comprising stop gain/loss, splicing variants, and frameshift mutations. Generally, the deleterious variants included LoF and Dmis in our study.

### 2.4 Genotype–Phenotype Association Analysis

In the sEOPD & FPD cohort, PD patients were divided into carrier, and noncarrier groups based on the presence of gene variants. The relationship between carrier status and various motor or nonmotor scales (details provided in Table S2), such as UPDRS, Hoehn‐Yahr, NMSS, and SCOPA‐AUT, was analyzed by using linear and logistic regression and corrected with age, sex, and disease duration in PLINK.

### 2.5 Bioinformatics Analysis of Nogo‐Associated Genes in PD Database

The PD‐related datasets (GSE205450 and GSE68719) were obtained from the GEO database (https://www.ncbi.nlm.nih.gov/geo/). The samples in GSE205450 were the caudate and putamen of postmortem PD and control, and the samples in GSE68719 were the human prefrontal cortex (Brodmann Area 9) of PD and control. Differential expression genes (DEGs), meeting the criteria of *p*‐adjusted < 0.05 and |logFC| > 0.15, were screened and identified using GEO2R (https://www.ncbi.nlm.nih.gov/geo/geo2r/). Additionally, overlapping DEGs between the two datasets were visualized via a Venn diagram. Based on the overlapping DEGs, GO functional annotation and KEGG analysis were performed via the Hiplot website (https://hiplot.com.cn/home/index.html). Furthermore, the expression levels of Nogo‐associated genes in the two datasets, with significant differences in rare and common variant analysis, were retrieved from the GEO database. Statistical analysis of the relative expression of these genes between healthy controls and PD patients was accomplished via GraphPad software, with *t*‐tests.

### 2.6 Cell Culture and Immunofluorescence Staining

SH‐SY5Y cell differentiation was induced by retinoic acid (RA, 10 μM, 7 days) and then equally seeded into 6‐well plates and cultured in DMEM‐F12 medium (Gibco) supplemented with 20% FBS (Procell). After 24 h of incubation and treatment, immunofluorescence staining was assessed. The cells were incubated with mitoTracker (Invitrogen/Thermo) to show the mitochondria (37°C, 30 min). Then, cells were fixed in 4% PFA for 15 min, followed by permeabilization with 0.1% Triton X‐100 for 10 min. Subsequently, the cells were blocked with 1% BSA for 1 h and then incubated overnight at 4°C with anti‐Tuj1 antibody (Proteintech), followed by incubation with secondary antibody Alexa Fluor 488 goat anti‐mouse IgG (Invitrogen/Thermo). The nuclei were stained with Hoechst dye (Life Technologies). Immunofluorescence images were captured using an ApoTome.2 Microscope (Zeiss). Image processing and neurite length analysis were performed by ImageJ software. Statistical analysis of neurite length was accomplished via GraphPad software with one‐way ANOVA.

### 2.7 Statistical Analysis

Descriptive statistics were used to summarize the data, with measurement data presented as mean ± standard deviation and enumeration data as percentages. All data, which were used with parametric equivalent, have been subject to testing for normality with the Shapiro–Wilk test. Gene burden analysis was performed via Sequence Kernel Association Test‐optimal (SKAT‐O) and Fisher test to compare rare variants of Nogo‐associated genes between patients and healthy controls. Besides, gender and the first five principal components were considered as covariates to adjust in the sEOPD & FPD cohorts. Logistic regression analysis was utilized to assess the relationship between single common variants and PD risk, corrected with age, sex, and disease duration in PLINK. Correction for multiple comparisons was applied using the false discovery rate (FDR) for rare variant analysis (FDR‐*p* < 0.05) and Bonferroni correction for common variant analysis and genotype–phenotype association analysis (significant level *α* < 0.05/n).

## 3 Results

### 3.1 Demographic Characteristics

In our study, we stratified PD patients and healthy controls into two cohorts: the sEOPD & FPD cohort, comprising 1997 cases and 1652 controls, and the sLOPD cohort, comprising 1962 cases and 1279 controls. Specifically, the key demographic characteristics, including age, age at onset, and gender, were described in Table 1.

**TABLE 1:** The basic demographic characteristics of included subjects.

Group	sEOPD & FPD cohort		sLOPD cohort	
	Case (*n* = 1997)	Control (*n* = 1652)	Case (*n* = 1962)	Control (*n* = 1279)
Age	52.14 ± 8.87	62.03 ± 12.59	66.76 ± 7.08	62.32 ± 7.11
Age at onset	46.33 ± 8.18	—	61.88 ± 6.93	—
Gender (male/female)	1091/906	795/857	984/978	613/666

### 3.2 Burden Analysis of Rare Variants

The Nogo‐associated genes were subdivided into ligand, receptor, and signal pathway groups according to their function. Initially, we implemented the SKAT‐O test and Fisher test to, respectively, analyze the aggregation burden of rare variants in these genes. As shown in Table 2, Tables S3 and S4, deleterious variants within the receptor subtype of Nogo‐associated genes showed a significant association with PD in the sEOPD & FPD cohort (SKAT‐O *p* = 0.044), suggesting a potential protective effect (95% CI 0.69–0.99). However, we failed to identify the association among the other subtypes in the two cohorts.

**TABLE 2:** The aggregation burden analysis of Nogo‐related genes subset in the two cohorts.

Group	sEOPD & FPD cohort				sLOPD cohort			
	Missense	Dmis	LoF	Deleterious variants	Missense	Dmis	LoF	Deleterious variants
Ligand	62 (0.842)	42 (0.730)	2 (1.000)	44 (0.789)	45 (0.837)	33 (0.893)	1 (0.488)	34 (0.836)
Receptor	308 (0.096)	211 (0.075)	21 (1.000)	232 (0.044)	231 (0.664)	157 (0.699)	5 (0.488)	162 (0.691)
Signal pathway	175 (0.096)	154 (0.127)	3 (1.000)	157 (0.210)	126 (0.837)	112 (0.893)	4 (0.488)	116 (0.836)

*Note:* Data were described as the number of variants (MAF < 0.01). *N* (FDR‐P): *N* is the total number of rare variants identifed in Nogo‐associated genes, FDR‐P is the *p* value of SKAT‐O test after FDR correction. The statistically significant *p*‐value was shown in bold text after FDR correction (FDR‐*p* value < 0.05).

Abbreviations: All, all variants; Dmis, damaging missense; Deleterious, Dmis+LoF; LoF, loss‐of‐function variant; MAF, minor allele frequency; Missense, missense variant.

**FIGURE 2:**
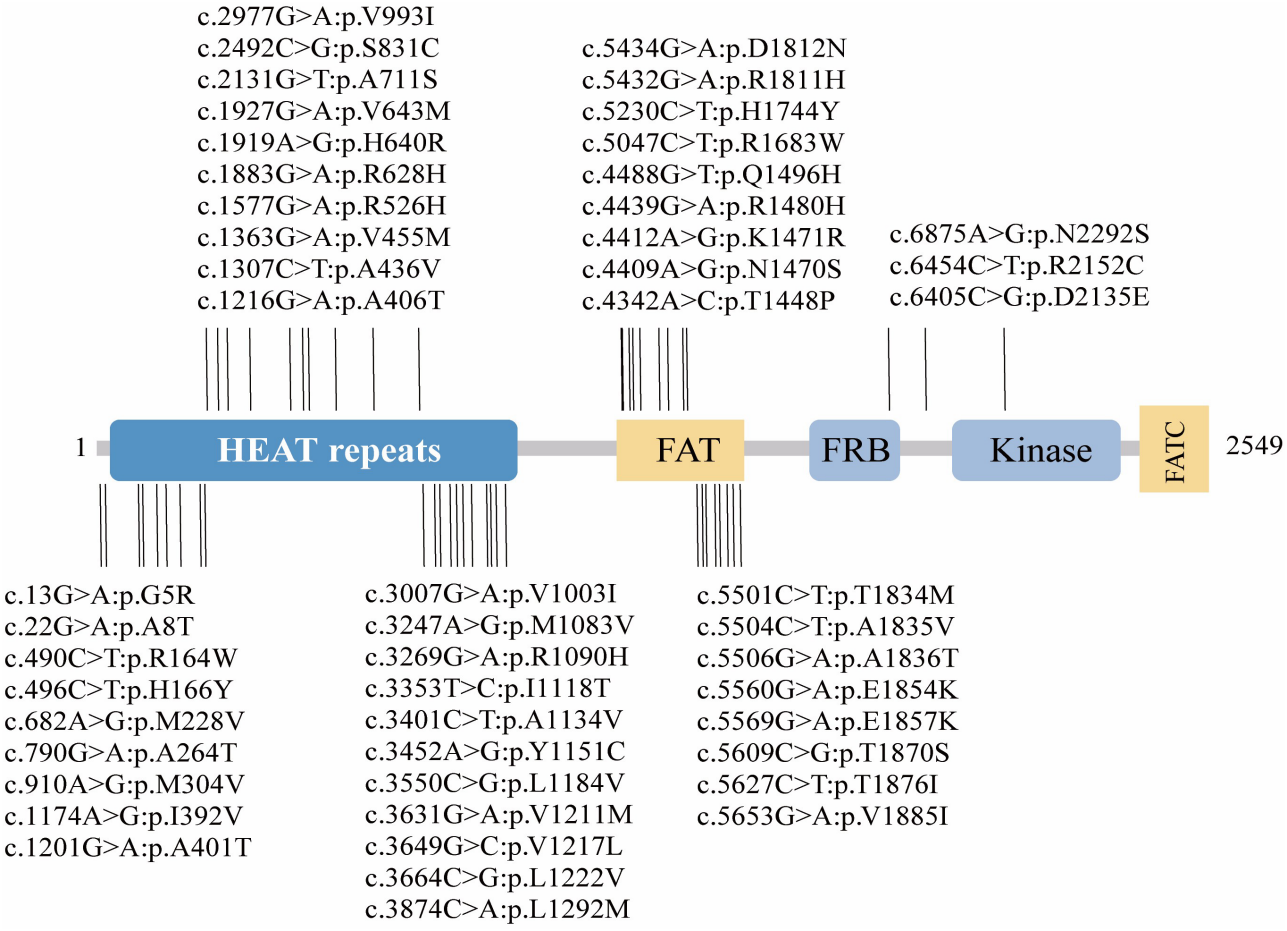
The schematic diagram of mTOR protein and the location of rare missense variants identified in the sEOPD & FPD cohort. The domain of mTOR protein contains HEAT repeats, FRAP‐ATM‐TTRAP (FAT) domain, FRB domain, kinase domain, and FATC domain. Mutations in this study of the *MTOR* gene are shown above the schematic. Mutation of the *MTOR* gene is only found in *Patients, ^#^Healthy controls, and ^^^Both patients and healthy controls.

To explore the individual contributions of Nogo‐associated genes to PD risk, we conducted the SKAT‐O analysis and Fisher test in both cohorts. In the sEOPD & FPD cohort, we found significant associations between rare missense variants in the *LILRB1* gene (SKAT‐O *p* = 0.034), *MTOR* gene (SKAT‐O *p* = 0.034), and PD risk after FDR correction (Table 3). Specifically, rare missense variants in the *MTOR* gene may increase PD risk (95% CI 1.20–2.91), while those in the *LILRB1* gene did not significantly impact PD susceptibility (95% CI 0.75–1.34), as shown in Table S5. Nevertheless, no correlation was observed between rare variants of Nogo‐associated genes and PD in the sLOPD cohort (Table 4, Table S6). The specific rare variants of Nogo‐related genes identified in the sEOPD & FPD and sLOPD cohort were shown in Tables S2 and S8. Additionally, we mapped the structure domain of the mTOR protein and highlighted the rare missense variants of the *MTOR* gene in the corresponding domain, which were all found in our cohort (Figure 2). Our results unveiled that most missense variants were located in the HEAT repeat domain and FAT domain, suggesting potential functional implications.

**TABLE 3:** The burden analysis of Nogo‐related genes rare variants in the sEOPD & FPD cohort.

Gene	sEOPD & FPD cohort				
	All	Dmis	LoF	Deleterious variants	Missense
RTN4	91 (0.938)	42 (0.905)	2 (1.000)	44 (0.905)	62 (0.904)
RTN4R	60 (0.905)	20 (0.905)	—	20 (0.905)	35 (0.904)
LINGO1	39 (0.905)	14 (0.640)	—	14 (0.640)	16 (0.642)
NGFR	47 (0.905)	24 (0.905)	4 (0.938)	28 (0.905)	28 (0.757)
LILRB1	116 (0.147)	18 (0.849)	11 (0.938)	29 (0.773)	**69 (0.034)**
PLXNA2	151 (0.876)	75 (0.161)	3 (0.416)	78 (0.136)	84 (0.281)
DPYSL2	35 (0.905)	13 (0.905)	—	13 (0.905)	16 (0.642)
ITGAV	61 (0.876)	35 (0.253)	1 (0.938)	36 (0.281)	44 (0.510)
S1PR2	43 (0.905)	12 (0.849)	2 (0.938)	14 (0.830)	16 (0.642)
RHOA	2 (0.905)	1 (0.870)	—	1 (0.870)	1 (0.757)
ROCK1	50 (0.905)	28 (1.000)	—	28 (1.000)	29 (1.000)
LIMK1	56 (0.361)	23 (0.849)	1 (0.764)	24 (0.830)	31 (0.642)
NTRK2	41 (0.905)	13 (0.855)	—	13 (0.855)	15 (0.757)
PIK3CA	31 (1.000)	10 (0.905)	—	10 (0.905)	13 (0.904)
PIK3CB	46 (0.905)	25 (0.905)	—	25 (0.905)	29 (0.904)
AKT1	31 (0.905)	7 (0.905)	—	7 (0.905)	7 (0.904)
MTOR	106 (0.147)	47 (0.074)	2 (1.000)	49 (0.281)	**50 (0.034)**

*Note:* Data were described as the number of variants (MAF < 0.01). *N* (FDR‐P): *N* is the total number of rare variants identifed in Nogo‐associated genes, FDR‐P is the *p* value of SKAT‐O test after FDR correction. The statistically significant *p*‐value was shown in bold text after FDR correction (FDR‐*p* value < 0.05).

Abbrevaitions: All, all variants; Deleterious, Dmis+LoF; Dmis, damaging missense; LoF, loss‐of‐function variant; MAF, minor allele frequency; Missense, missense variant.

**TABLE 4:** The burden analysis of Nogo‐related genes rare variants in the sLOPD cohort.

Gene	sLOPD cohort				
	All	Dmis	LoF	Deleterious variants	Missense
RTN4	68 (0.898)	33 (0.904)	1 (0.651)	34 (0.904)	45 (0.903)
RTN4R	53 (0.898)	20 (0.904)	—	20 (0.904)	34 (0.903)
LINGO1	28 (0.898)	9 (0.904)	—	9 (0.904)	13 (0.903)
NGFR	30 (0.898)	15 (0.904)	—	15 (0.904)	15 (0.903)
LILRB1	86 (0.557)	12 (0.407)	5 (0.651)	17 (0.523)	56 (0.635)
PLXNA2	118 (0.898)	61 (0.904)	0 (1.000)	61 (0.904)	67 (0.903)
DPYSL2	32 (0.557)	8 (0.317)	—	8 (0.383)	11 (0.635)
ITGAV	33 (0.898)	21 (0.904)	—	21 (0.904)	23 (0.903)
S1PR2	25 (0.898)	11 (0.904)	—	11 (0.904)	12 (0.903)
RHOA	14 (0.898)	—	—	—	3 (0.903)
ROCK1	33 (0.898)	17 (0.317)	1 (0.651)	18 (0.383)	19 (0.635)
LIMK1	42 (0.898)	17 (0.904)	1 (0.651)	18 (0.904)	20 (0.903)
NTRK2	34 (0.898)	14 (0.904)	0 (1.000)	14 (0.904)	15 (0.903)
PIK3CA	13 (0.898)	4 (0.904)	—	4 (0.904)	7 (0.903)
PIK3CB	28 (0.898)	15 (0.904)	—	15 (0.904)	16 (0.903)
AKT1	36 (0.898)	8 (0.904)	1 (0.651)	9 (0.904)	8 (0.903)
MTOR	78 (0.898)	37 (0.904)	1 (0.651)	38 (0.904)	38 (0.903)

*Note:* Data were described as the number of variants (MAF < 0.01). *N* (FDR‐P): *N* is the total number of rare variants identifed in Nogo‐associated genes, FDR‐P is the *p* value of SKAT‐O test after FDR correction.

Abbrevaitions: All, all variants; Deleterious, Dmis+LoF; Dmis, damaging missense; LoF, loss‐of‐function variant; MAF, minor allele frequency; Missense, missense variant.

### 3.3 Association Analysis of Common Variants

Common variants with MAF < 0.01 were analyzed. A total of 219 common variants were identified in the sEOPD & FPD cohort and 2256 in the sLOPD cohort. Manhattan plots were mapped to visualize the distribution of common variants in this study with thresholds set at 0.01/*N* and 0.05/*N* (Figure S2), where *N* represented the number of common variants in each group. However, none of the common variants reached statistical significance in relation to PD risk in either cohort.

### 3.4 Genotype–Phenotype Association Analysis

From the above results, the missense variants of *MTOR* genes may be involved in PD. Next, we conducted a genotype–phenotype association analysis to explore whether *MTOR* gene mutation modified the phenotypes of PD. Intriguingly, the PD patients carried with the missense, Dmis, and deleterious variants had milder motor symptoms, including a lower UPDRS‐III score, bradykinesia score, and H‐Y stage, while the PD patients carried with rare missense variants in *the MTOR* gene were more susceptible to dyskinesia. However, there was no statistically significant difference after the Bonferroni correction, just suggestive. The clinical manifestations of carriers and noncarriers were depicted in Table 5.

**TABLE 5:** Genotype–phenotype association analysis of *MTOR* gene rare variants in the sEOPD & FPD cohort.

Clinical characteristics	Missense				Dmis				LoF				Deleterious			
	Carrier (*n* = 67)	Non‐carrier (*n* = 1930)	*p*	*β*/OR	Carrier (*n* = 64)	Non‐carrier (*n* = 1933)	*p*	*β*/OR	Carrier (*n* = 24)	Non‐carrier (*n* = 1973)	*p*	*β*/OR	Carrier (*n* = 88)	Non‐carrier (*n* = 1909)	*p*	*β*/OR
Age at onset	45.57 ± 7.213	46.37 ± 8.212	0.290	−1.098	46.03 ± 6.551	46.35 ± 8.228	0.532	−0.665	48.46 ± 8.011	46.31 ± 8.176	0.304	1.748	46.76 ± 7.072	46.32 ± 8.229	0.988	0.014
UPDRS‐I	2.27 ± 1.776	2.40 ± 2.063	0.771	−0.075	2.22 ± 1.682	2.40 ± 2.065	0.614	−0.134	2.27 ± 1.804	2.40 ± 2.057	0.838	−0.088	2.23 ± 1.705	2.40 ± 2.068	0.588	−0.123
UPDRS‐II	9.85 ± 5.560	11.66 ± 6.723	0.069	−1.371	9.76 ± 5.519	11.66 ± 6.722	0.063	−1.432	10.55 ± 4.906	11.61 ± 6.713	0.712	−0.463	9.98 ± 5.341	11.67 ± 6.741	0.073	−1.187
UPDRS‐III	21.87 ± 13.642	26.98 ± 15.709	**0.023**	−4.193	21.56 ± 13.593	26.98 ± 15.706	**0.019**	−4.450	22.73 ± 16.216	26.86 ± 15.660	0.338	−2.940	21.88 ± 14.257	27.03 ± 15.697	**0.011**	−4.110
Tremor score	2.79 ± 3.595	3.79 ± 3.717	0.063	−0.873	2.73 ± 3.633	3.79 ± 3.716	0.053	−0.931	2.91 ± 2.793	3.76 ± 3.726	0.355	−0.722	2.78 ± 3.410	3.80 ± 3.725	**0**.**031**	−0.890
Stiffness score	4.50 ± 3.607	5.53 ± 4.215	0.104	−0.849	4.47 ± 3.697	5.53 ± 4.211	0.109	−0.855	5.86 ± 4.950	5.50 ± 4.190	0.459	0.641	4.85 ± 4.090	5.53 ± 4.203	0.326	−0.451
Bradykinesia score	8.06 ± 6.070	10.01 ± 6.592	**0.040e**	−1.644	7.92 ± 5.787	10.01 ± 6.599	**0**.**030**	−1.776	7.41 ± 6.412	9.98 ± 6.581	0.103	−2.166	7.78 ± 5.927	10.04 ± 6.596	**0.006**	−1.919
Postural instability score	3.24 ± 2.565	3.88 ± 3.101	0.189	−0.468	3.19 ± 2.576	3.88 ± 3.099	0.157	−0.516	2.86 ± 3.060	3.87 ± 3.085	0.180	−0.792	3.10 ± 2.700	3.90 ± 3.099	0.054	−0.603
H‐Y stage	1.89 ± 0.771	2.15 ± 0.853	**0.024**	−0.218	1.86 ± 0.772	2.15 ± 0.853	**0.013**	−0.246	1.977 ± 0.8657	2.14 ± 0.8515	0.536	−0.099	1.89 ± 0.795	2.151 ± 0.853	**0.014**	−0.210
Dyskinesia	23.3%	14.6%	**0.040**	1.970	22.4%	14.7%	0.056	1.914	9.1%	15.0%	0.600	0.657	18.8%	14.7%	0.155	1.560
Wearing‐off	30.2%	21.2%	0.106	1.607	30.0%	21.2%	0.127	1.581	12.5%	21.5%	0.351	0.548	25.3%	21.3%	0.406	1.251
Freezing gait	21.7%	25.9%	0.593	0.839	22.4%	25.9%	0.720	0.888	18.2%	25.9%	0.568	0.720	21.3%	26.0%	0.542	0.839
MMSE	27.50 ± 3.008	27.13 ± 3.098	0.632	0.193	27.41 ± 3.067	27.14 ± 3.096	0.752	0.131	26.18 ± 4.004	27.16 ± 3.082	0.283	−0.764	27.10 ± 3.337	27.15 ± 3.083	0.787	−0.098
PDSS	117.73 ± 31.185	116.89 ± 28.524	0.943	0.290	120.76 ± 27.550	116.79 ± 28.649	0.410	3.423	109.24 ± 37.627	117.02 ± 28.485	0.202	−8.644	117.65 ± 30.706	116.89 ± 28.519	0.972	0.128
RBDQ‐HK	13.13 ± 13.310	13.21 ± 16.160	0.818	0.527	12.25 ± 12.730	13.24 ± 16.168	0.844	−0.465	16.29 ± 23.331	13.17 ± 15.958	0.383	3.290	13.38 ± 16.268	13.20 ± 16.061	0.765	0.605
ESS	6.54 ± 5.107	7.14 ± 6.116	0.880	−0.128	6.60 ± 4.915	7.14 ± 6.120	0.915	−0.094	6.65 ± 6.809	7.13 ± 6.075	0.785	−0.389	6.61 ± 5.439	7.15 ± 6.113	0.813	−0.178
HRS	20.63 ± 6.470	20.10 ± 6.150	0.896	0.118	20.67 ± 6.519	20.10 ± 6.149	0.864	0.158	20.65 ± 4.663	20.11 ± 6.178	0.736	0.507	20.66 ± 6.030	20.09 ± 6.168	0.745	0.258
HAMD	5.68 ± 4.737	5.66 ± 5.561	0.720	−0.282	5.50 ± 6.519	5.67 ± 5.563	0.580	−0.449	6.12 ± 5.776	5.66 ± 5.531	0.605	0.692	5.67 ± 4.905	5.67 ± 5.562	0.836	−0.145
PDQ‐39	27.08 ± 22.479	28.66 ± 25.651	0.728	−1.186	26.11 ± 21.452	28.69 ± 25.672	0.550	−2.107	22.59 ± 24.650	28.68 ± 25.547	0.416	−4.627	25.15 ± 22.222	28.77 ± 25.685	0.344	−2.860

*Note:* The results are adjusted for age, sex, and disease duration. *p* value is the *p* value after logistic/linear regression analysis; *β*/OR, *β* = ln (OR), and the OR is the odds ratio. Dyskinesia, wearing‐off, and freezing gait are shown via OR, and other scales are *β*. The bold values in the table are *p* < 0.05, with statistical significance.

Abbreviations: All, all variants; Deleterious variants, Dmis + LoF; Dmis, damaging missense; ESS, Epworth Sleepiness Scale; HAMD, Hamilton Depression Scale; HRS, hyposmia rating scale; LoF, loss‐of‐function variant; Missense, missense variant; MMSE, mini‐mental state examination; PDQ39, the Parkinson's Disease Questionnaire; PDSS, Parkinson's Disease Sleep Scale; PFS, Parkinson Fatigue Scale; RBDQ‐HK, REM Sleep Behavior Disorder Screening Questionnaire‐Hong Kong; SCOPA‐AUT, the Scale for Outcomes in Parkinson's Disease for Autonomic Symptoms; UPDRS, Unified Parkinson's Disease Rating Scale.

### 3.5 mTOR Involvement in PD by Regulating Axon Growth

**FIGURE 3:**
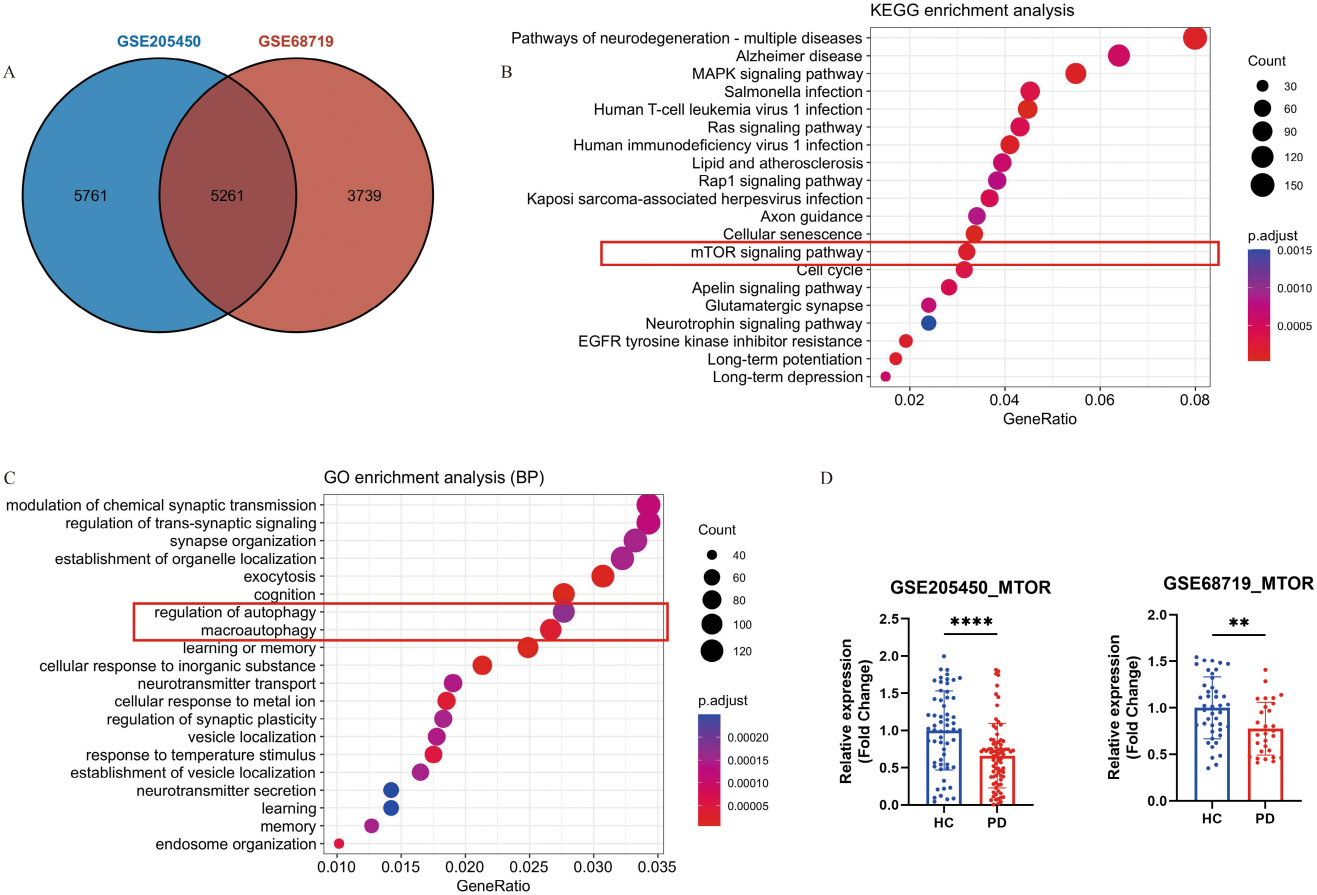
Identification of the involvement of mTOR in Parkinson's disease via the GEO database. (A) Venn diagram of DEGs from the two PD datasets, with *p*‐adjusted < 0.05 and∣logFC∣ > 0.15 as the threshold. (B) KEGG pathway enrichment analysis (TOP 20) of the overlapping DEGs (5261 genes) in PD, with BH correction. (C) GO enrichment analysis (TOP 20) of the overlapping DEGs (5261 genes) in PD, with BH correction. (D) The relative mRNA expression of the *MTOR* gene in the two PD databases, with *t‐*tests. **p < 0.01, and ****p < 0.0001.

Aimed at exploring whether the mTOR pathway is involved in PD, we initially selected two PD datasets (GSE205450 and GSE68719) from the GEO database [35, 36]. As depicted in Figure 3, a total of 5261 overlapping DEGs were identified between the two datasets. Subsequent KEGG and GO enrichment analysis of these 5261 DEGs revealed enrichment in the mTOR signaling pathway and autophagy process, suggesting their relevance to PD. Moreover, the relative mRNA expression of the *MTOR* gene was significantly decreased in PD, indicating a potential negative role of mTOR in PD pathogenesis.

**FIGURE 4:**
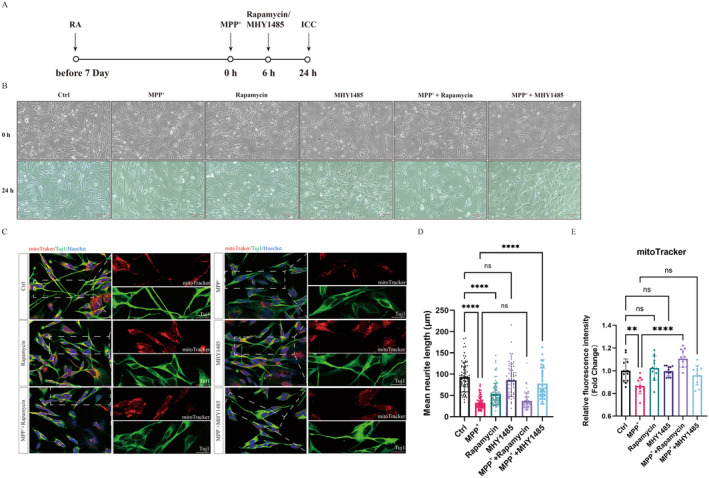
mTOR protein is involved in Parkinson's disease by regulating cell growth and axon growth. After differentiation via RA (10 μM, 7 days), SH‐SY5Y cells were treated with MPP^+^ (500 μM, 24 h), Rapamycin (2.5 μM, 18 h), or MHY1485 (10 μM, 18 h) to detect neurite length and cell death. (A) The procedure of cell plating and drug handling. (B) This bright field image of cells was taken, respectively, at 0 and 24 h with MPP^+^ treatment. Scale bar, 100 μm. (C) SH‐SY5Y cells were stained with Tuj1 (green), mitoTracker (red), and Hoechst (blue). (D and E) the quantification of axon length (Tuj1) and mitoTracker intensity (mitoTracker), analyzed by ImageJ and visualized by GraphPad Prism 9. Scale bar, 20 μm. Data were presented as the mean ± SD. ****p < 0.0001, **p < 0.01, ns, no different significance.

Based on these findings, we proceeded to explore the impact of mTOR on cell growth and axon growth, as detailed in Figure 4A. Treatment with MPP^+^ led to significant growth inhibition in SH‐SY5Y cells, which was exacerbated by mTOR inhibitor rapamycin but slightly mitigated by the mTOR activator MHY1485 (Figure 4B). Furthermore, MPP^+^ or rapamycin treatment resulted in reduced neurite length, which was rescued by MHY1485 (Figure 4C,D). Last, MPP^+^ treatment caused a decrease in mitoTracker intensity, while rapamycin significantly improved mitoTracker intensity but not MHY1485 (Figure 4E). These results suggest a complex interaction among mTOR, axon growth, and PD pathogenesis, highlighting the potential therapeutic implications of modulating the mTOR pathway in PD treatment.

## 4 Discussion

Axonal injury is a prominent feature observed in many neurodegenerative diseases, especially in PD [37, 38, 39]. Studies have highlighted the presence of α‐synuclein aggregation in axons, dendritic defects, and fragmentation in induced pluripotent stem cells (iPSC) derived from PD patients [40]. Meanwhile, the long‐term enhancement of synapses in PD patients is weakened, resulting in reduced synaptic plasticity [41]. Previous studies revealed that Nogo‐associated genes have been implicated in inhibiting neuronal axon growth through ligand‐receptor interactions, mediated by downstream signaling pathways such as the RhoA‐ROCK1 and PI3K‐Akt–mTOR pathways [18, 19, 20]. Given these insights, our study aimed to investigate the role of Nogo‐associated genes, particularly focusing on the related signaling pathway, in PD susceptibility and phenotype modulation.

Our analysis revealed a significant association between rare missense variants of the *MTOR* gene and increased PD risk in the sEOPD & FPD cohorts. Genotype–phenotype association analysis showed that PD patients carried with *MTOR* gene variants exhibited milder motor symptoms but were more susceptible to dyskinesia, indicating a potential role of the *MTOR* gene in modulating PD phenotypes. Furthermore, bioinformatics analysis and basic experiments suggested that *MTOR* gene defects may contribute to PD pathogenesis by interfering with axon growth regulation.

Nevertheless, there is much debate about the role of the mTOR signaling pathway in PD. Some studies showed the reduced Akt–mTOR signal pathway activity in PD animal models induced by H_2_O_2_, MPTP, and 6‐OHDA treatment, but others have yielded conflicting results [42, 43, 44, 45]. From a genetic point of view, the combination of *RPTOR* rs11868112, *RPS6KA2* rs6456121, and *SNCA* rs356219 could collectively cause an earlier age at the onset of PD, of which the *RPTOR* gene expresses Raptor protein, one component of the mTORC1 complex, and the *RPS6KA2* gene expresses RSK3 protein, engaged in the activation of the mTOR pathway [46]. Besides, mTOR can enhance the translation of universal protein, promoting axon growth and synaptic plasticity via coordinate eukaryotic translation initiation factor eIF4E, so mTOR is inhibited in the Nogo‐associated signaling pathway [47]. Likewise, RTP801, which inhibits mTOR activation, is significantly elevated in the substantia nigra of PD patients and leads to cell death [48]. Our findings supported that dysregulation of mTOR activity, as evidenced by missense variants in the *MTOR* gene, may contribute to PD susceptibility and dyskinesia. Besides, the rescue of neurite length following treatment with a mTOR activator further identified the importance of mTOR in axon growth regulation and PD pathogenesis. However, our study didn't explore the specific mechanism about how mTOR dysfunction causes the PD pathogenesis, which needs further identification.

Intriguingly, our study identified a protective association between rare deleterious variants in the receptor subtype of Nogo‐associated genes and PD risk in the sEOPD & FPD cohort (*p* = 0.044, 95% CI 0.69–0.99). In our study, the receptor subtype of Nogo‐associated genes contained eight genes, which were full of receptors of Nogo‐A. As described above, Nogo‐A plays a negative regulatory role in axon growth through their ligand‐receptor interactions. These findings suggest that alterations in Nogo receptor function may attenuate the inhibitory effects of Nogo ligands on axon growth, potentially reducing PD susceptibility. Consistent with our results, previous studies have implicated Nogo receptor variants, such as NGFR, in reducing PD risk [49]. Another study discovered that increasing the expression of NGFR protein could cause the death of dopaminergic neurons via inhibiting the expression of Erk1/2, which further illustrated that *NGFR* gene variants might reduce the risk of PD by regulating the function of p75 protein [50]. Furthermore, a genetic study identified that *LINGO1* rs11856808 and rs9652490 could improve the risk of PD. In contrast, other studies conducted in Italy and America concluded that the two variants played a protective role in PD onset, and *LINGO1* rs9652490 was not associated with PD onset in the Chinese population [51, 52, 53]. Therefore, further research is warranted to elucidate the precise mechanisms underlying the protective effects of Nogo receptors in PD pathogenesis.

To sum up, our study demonstrated that the mutation in the *MTOR* gene might increase the risk of PD, and the mutation in the receptor subtype of Nogo‐associated genes might reduce the risk of PD, which enlarged the genetic spectrum of the Nogo‐associated genes. In addition, we conducted an initial exploration on the correlation between Nogo‐associated genes and PD in the Chinese population, from the perspective of the signaling pathway. Our study benefits from a large sample size, comprehensive rare and common variant analysis, and genotype–phenotype association assessment, providing valuable insights into the genetic basis of PD in the Chinese population. Moreover, the inclusion of different PD subtypes enhances our understanding of genetic heterogeneity in PD. However, limitations include the lack of analysis of copy number variants, the need for validation in independent cohorts, and the absence of mechanistic studies to elucidate the specific roles of identified variants in PD pathogenesis. Besides, it is necessary to identify the axon growth in the *MTOR* gene knock‐in cell models and animal models in the further study. In conclusion, our findings highlight the potential role of the *MTOR* gene in PD susceptibility and phenotype, and also suggest a protective effect of Nogo receptors in PD risk. Further research is warranted to validate these findings in larger cohorts, investigate copy number variants, and elucidate the underlying mechanisms of identified genetic associations in PD pathogenesis.

## Author Contributions

**Xiurong Huang:** data curation, formal analysis, methodology, writing – original draft. **Yige Wang:** data curation, methodology, writing – review and editing. **Yaqin Xiang:** data curation, writing – review and editing. **Yuwen Zhao:** data curation, writing – review and editing. **Hongxu Pan:** data curation, writing – review and editing. **Zhenhua Liu:** data curation. **Qian Xu:** data curation, funding acquisition. **Qiying Sun:** data curation, funding acquisition. **Jieqiong Tan:** data curation, funding acquisition. **Xinxiang Yan:** data curation, funding acquisition. **Jinchen Li:** funding acquisition, writing‐review & editing. **Beisha Tang:** project administration, supervision, funding acquisition, writing – review and editing. **Jifeng Guo:** project administration, supervision, funding acquisition, writing – review and editing.

## Ethics Statement

This study was approved by the Ethics Committee of Xiangya Hospital of Central south university.

## Consent

All participants have signed informed consent.

## Conflicts of Interest

The authors declare no conflicts of interest.

## Supporting information


Data S1.

**Figure S1.** Flowchart of this study. Subjects in the sEOPD & FPD cohorts underwent Whole exome sequence (WES) and Subjects in the sLOPD cohort underwent whole genome sequence (WGS). Based on above two cohorts, we aimed to systematically screen and identify Nogo‐associated genes. Gene‐based burden analysis (SKAT‐O and Fisher tests) were used to identify the relationship between rare variants of Nogo‐associated genes and PD risk. Single variant association test (Logistic regression) were used to identify the relationship between common variants of Nogo‐associated genes and PD risk. Next, genotype–phenotype association analysis in our study was complemented by Linear or Logistic regression, followed by GEO datasets analysis and basic experiments in PD model.

**Figure S2.** Summary of single common variants in sEOPD & FPD cohort and sLOPD cohort. A, Manhattan plot of all common variants in the sEOPD & FPD cohort; B, QQ graph of all common variants in the sEOPD & FPD cohort; C, Manhattan plot of all common variants in the sLOPD cohort; D, QQ graph of all common variants in the sLOPD cohort.

## Data Availability

The cohort datasets used or analyzed are available from the corresponding author on reasonable request, and the GEO datasets are available from the GEO database.
