# Supplementary material for: Genetic Analysis of Neurite Outgrowth Inhibitor‐Associated Genes in Parkinson's Disease: A Cross‐Sectional Cohort Study
Source: CNS Neurosci Ther. 2024 Oct 2;30(10):e70070. doi: 10.1111/cns.70070 (PMC11445604; doi:10.1111/cns.70070)
Supplement: Supplementary file 1 — Data S1. [file CNS-30-e70070-s002.docx]

**SUPPLEMENTARY MATERIALS**

**Supplemental methods**

**Whole-exome sequencing and Whole-genome sequencing**

We collected peripheral blood from the outpatient of Xiangya hospital and extracted genomic DNA via following standard protocol. Based on the research methods in our previous studies, whole-exome sequencing (WES) and whole-genome sequencing (WGS) were conducted on the sEOPD & FPD cohort and sLOPD cohort respectively (Guo et al., 2018; Pan et al., 2020; Zhao et al., 2020). Specifically, WES were conducted by using SureSelect Human All Exon Kit V6 (Agilent) to catch the whole-exome DNA and make sample libraries ready, followed by paired-end 2×150 bp sequencing with Illumina HiSeq X10 (Illumina, San Diego, CA), and 123-fold of average sequencing depth. In addition, WGS were sequenced via the Illumina Nova sequencing platform in paired-end 2×150 bp mode, with 12-fold average sequencing depth. Besides, we performed sequencing data according to a bioinformatics method, which was commonly used for next-generation sequencing (NGS) data processing and analysis. First, we handled paired-end sequencing reads to the human reference genome (hg19) by using the Burrows-Wheeler Aligner (Bernstein, Bernstein, Balakrishnan, & Korbee, 1989). Then, Picard tool (http://broadinsti tute.github.io/picard/) was applied to delete duplicate reads, as well as format conversing and indexing. Next, the Genome Analysis Toolkit (GATK) was employed to process single nucleotide variants (SNVs) and insertions/deletions, and excluded low-quality variants (Schunk et al., 1989). Last, we have annotated the variants with RefSeq (hg19) to describe each called variant, via ANNOVAR (Yang & Wang, 2015) and VarCards (Li et al., 2018), which was used to clarify the variant position, predicted cDNA and Amino acid sequence alteration, allele frequencies in human populations, and pathogenicity prediction (Li et al., 2018; Yang & Wang, 2015).

**Quality control**

As we represented in our previous studies, PLINK software v1.90 were put into recode WES and WGS data into a binary PLINK input format for controlling individual and variant quality (Pan et al., 2020). We excluded the individuals who exhibited ambiguous sex (sex assignment conflict in PLINK), deviation from heterozygosity/genotype detection, low genotype detection rates, cryptic relatedness, or different descent (non-Chinese). In addition, low-quality genotypes would be removed when the genotype quality score (GQ) on the Phred scale lowering than 20, the allele depth (AD) lowering than five, the read depth (DP) of the WES data lowering than 10, GQ lowering than 15, AD lowering than two and DP lowering than five for WGS data and variants with low call rates (missing rate > 5%) or deviation from Hardy-Weinberg equilibrium (P < 1×10^−3^). When the missense variant was predicted by CADD to evaluate the damaging, the missense variants with C-score> 12.37 were regarded as Damaging variants. Finally, they would be used for further analysis after individuals and variants passing the QC thresholds.

**Supplementary Table 1 The basic characteristics of involved genes**

| category | Gene | Protein | Cytogenetic location | Genomic coordinates（hg19） | Refseq |
| --- | --- | --- | --- | --- | --- |
| Ligand | *RTN4* | Nogo-A  Nogo-B  Nogo-C | 2p16.1 | Chr2:55199325-55364967 | NM_020532.5  NM_153828.3 NM_007008.3 |
|  |  |  |  |  |  |
| Receptor | *RTN4R* | NgR | 22q11.21 | Chr22:20228938-20255841 | NM_023004.6 |
|  | *LINGO1* | LINGO1 | 15q24.3 | Chr15:77905369-78113242 | NM_001301186.2 |
|  | *NGFR* | p75^NTR^ | 17q21.33 | Chr17:47572655-47592370 | NM_002507.4 |
|  | *LILRB1* | PirB | 19q13.42 | Chr19:55128583-55149007 | NM_001081637.3 |
|  | *PLXNA2* | PlexinA2 | 1q32.2 | Chr1:208195587-208417729 | NM_025179.4 |
|  | *DPYSL2* | CRMP2 | 8p21.2 | Chr8:26371547-26515691 | NM_001197293.3 |
|  | *ITGAV* | Integrin αV | 2q32.1 | Chr2:187454783-187545628 | NM_001144999.3 |
|  | *S1PR2* | S1PR2 | 19p13.2 | Chr19:10332109-10342007 | NM_004230.4 |
|  |  |  |  |  |  |
| Signal pathway | *RHOA* | RhoA | 3p21.31 | Chr3:49396578-49449409 | NM_001313941.2 |
|  | *ROCK1* | ROCK1 | 18q11.1 | Chr18:18526867-18691774 | NM_005406.3 |
|  | *LIMK1* | LIMK1 | 7q11.23 | Chr7:73498134-73536855 | NM_001204426.2 |
|  | *NTRK2* | TrkB | 9q21.33 | Chr9:87283437-87641969 | NM_001007097.3 |
|  | *PIK3CA*  *PIK3CB* | PI3K | 3q26.32  3q22.3 | Chr3:178866145-178957881  Chr3:138371540-138553770 | NM_006218.4 NM_001256045.2 |
|  | *AKT1* | Akt | 14q32.33 | Chr14:105235686-105262085 | NM_001014431.2 |
|  | *MTOR* | mTOR | 1p36.22 | Chr1:11166592-11322608 | NM_001386500.1 |

**Supplementary Table 2 the concrete scales in our study**

| Category | Scales |
| --- | --- |
| Motor related scales | The Unified Parkinson's Disease Rating Scale（UPDRS） |
|  | Hoehn-Yahr stage |
|  | Freeze Gait Scale |
|  | Dyskinesia Scale |
|  |  |
| Non-motor related scales | Non-Motor Symptoms Scale（NMSS） |
|  | Functional gastrointestinal disorders scale |
|  | The Hamilton Depression Rating Scale |
|  | Cambridge-Hopkins questionnaire for restless legs syndrome（CH-RLSq） |
|  | The Hyposmia Rating Scale |
|  | The Parkinson Disease Sleep Scale（PDSS） |
|  | REM Sleep Behavior Disorder Questionnaire Hong Kong（RBDQ-HK） |
|  | Epworth Sleepiness Scale (ESS） |
|  | The 39-item Parkinson’s Disease Questionnaire（PDQ39） |
|  |  |
| Cognitive function | The Mini–Mental State Examination（MMSE） |

**Supplementary Table 3 The Fisher test of Nogo-related genes subset in the sEOPD & FPD cohorts**

| Gene | sEOPD & FPD cohort | | | | | | | | | | | | | | | |
| --- | --- | --- | --- | --- | --- | --- | --- | --- | --- | --- | --- | --- | --- | --- | --- | --- |
|  | Dmis | | | | Lof | | | | Deleterious | | | | Missense | | | |
|  | Case | Ctrl | p | LL, UL | Case | Ctrl | p | LL, UL | Case | Ctrl | p | LL, UL | Case | Ctrl | p | LL, UL |
| Ligand | 59 | 54 | 0.634 | 0.61, 1.33 | 2 | 6 | 0.357 | 0.03, 1.54 | 61 | 60 | 0.358 | 0.58, 1.22 | 81 | 66 | 0.934 | 0.72, 1.43 |
| Receptor | 256 | 248 | 0.106 | 0.70, 1.02 | 11 | 15 | 0.357 | 0.25, 1.41 | 267 | 263 | **0.075** | **0.69, 0.99** | 480 | 410 | 0.923 | 0.84, 1.11 |
| Signal pathway | 204 | 133 | 0.087 | 1.02, 1.62 | 48 | 39 | 1.000 | 0.65, 1.60 | 252 | 172 | 0.075 | 1.00, 1.51 | 221 | 144 | 0.070 | 1.03, 1.61 |

Data were described as the number of variants (MAF<0.01). The number of Case is the total number of alleles carried with all patients.The number of ctrl is the total number of alleles carried with all healthy controls. p is the P value of Fisher test after FDR correction, LL and UL is respectively the lower limit and upper limit of 95% confidence intervals in odds ratio (OR). Missense, missense variant; Dmis, damaging missense; LoF, loss-of-function variant; Deleterious, Dmis+LoF; MAF, minor allele frequency.

**Supplementary Table 4 The Fisher test of Nogo-related genes subset in the sLOPD cohorts**

| Gene | sLOPD cohort | | | | | | | | | | | | | | | |
| --- | --- | --- | --- | --- | --- | --- | --- | --- | --- | --- | --- | --- | --- | --- | --- | --- |
|  | Dmis | | | | Lof | | | | Deleterious | | | | Missense | | | |
|  | Case | Ctrl | p | LL, UL | Case | Ctrl | p | LL, UL | Case | Ctrl | p | LL, UL | Case | Ctrl | p | LL, UL |
| Ligand | 56 | 33 | 0.664 | 0.71, 1.76 | 3 | 0 | 0.749 | 0.27, - | 59 | 33 | 0.520 | 0.75, 1.85 | 69 | 41 | 0.694 | 0.73, 1.66 |
| Receptor | 229 | 134 | 0.664 | 0.90, 1.41 | 7 | 3 | 0.749 | 0.35, 9.13 | 236 | 137 | 0.520 | 0.91, 1.42 | 379 | 227 | 0.526 | 0.92, 1.31 |
| Signal pathway | 131 | 93 | 0.664 | 0.69, 1.21 | 6 | 6 | 0.749 | 0.17, 2.44 | 137 | 99 | 0.520 | 0.69, 1.18 | 141 | 104 | 0.526 | 0.67, 1.15 |

Data were described as the number of variants (MAF<0.01). The number of Case is the total number of alleles carried with all patients.The number of ctrl is the total number of alleles carried with all healthy controls. p is the P value of Fisher test after FDR correction, LL and UL is respectively the lower limit and upper limit of 95% confidence intervals in odds ratio (OR). Missense, missense variant; Dmis, damaging missense; LoF, loss-of-function variant; Deleterious, Dmis+LoF; MAF, minor allele frequency.

**Supplementary Table 5 The Fisher test of Nogo-related genes rare variants in the sEOPD & FPD cohort**

| Gene | sEOPD & FPD cohort | | | | | | | | | | | | | | | |
| --- | --- | --- | --- | --- | --- | --- | --- | --- | --- | --- | --- | --- | --- | --- | --- | --- |
|  | Dmis | | | | Lof | | | | Deleterious | | | | Missense | | | |
|  | Case | Ctrl | p | LL, UL | Case | Ctrl | p | LL, UL | Case | Ctrl | p | LL, UL | Case | Ctrl | p | LL, UL |
| *RTN4* | 59 | 54 | 0.903 | 0.61, 1.33 | 2 | 6 | 0.608 | 0.03, 1.54 | 61 | 60 | 0.909 | 0.58, 1.22 | 81 | 66 | 1.000 | 0.72, 1.43 |
| *RTN4R* | 25 | 25 | 0.903 | 0.45, 1.50 | - | - | - | - | 25 | 25 | 0.909 | 0.45, 1.50 | 53 | 44 | 1.000 | 0.65, 1.53 |
| *LINGO1* | 7 | 13 | 0.641 | 0.15, 1.20 | - | - | - | - | 7 | 13 | 0.641 | 0.15, 1.20 | 9 | 14 | 0.620 | 0.20, 1.32 |
| *NGFR* | 34 | 22 | 0.903 | 0.73, 2.30 | 2 | 2 | 1.000 | 0.06, 11.41 | 36 | 24 | 0.909 | 0.72, 2.18 | 95 | 58 | 0.401 | 0.97, 1.93 |
| *LILRB1* | 11 | 14 | 0.903 | 0.27, 1.54 | 6 | 8 | 0.905 | 0.18, 2.04 | 17 | 22 | 0.836 | 0.32, 1.26 | 110 | 91 | 1.000 | 0.75, 1.34 |
| *PLXNA2* | 67 | 81 | 0.204 | 0.48, 0.95 | 0 | 3 | 0.608 | 0.00, 2.00 | 67 | 84 | 0.177 | 0.47, 0.92 | 82 | 92 | 0.382 | 0.53, 1.00 |
| *DPYSL2* | 25 | 24 | 0.903 | 0.47, 1.58 | - | - | - | - | 25 | 24 | 0.909 | 0.47, 1.58 | 28 | 30 | 1.000 | 0.44, 1.34 |
| *ITGAV* | 42 | 29 | 0.903 | 0.73, 2.00 | 1 | 0 | 1.000 | 0.02, - | 43 | 29 | 0.909 | 0.75, 2.05 | 56 | 39 | 1.000 | 0.77, 1.85 |
| *S1PR2* | 45 | 40 | 0.903 | 0.59, 1.46 | 2 | 2 | 1.000 | 0.06, 11.41 | 47 | 42 | 0.909 | 0.60, 1.44 | 47 | 42 | 1.000 | 0.60, 1.44 |
| *RHOA* | 1 | 0 | 1.000 | 0.02, - | - | - | - | - | 1 | 0 | 1.000 | 0.02, Inf | 2 | 0 | 1.000 | 0.16, Inf |
| *ROCK1* | 35 | 26 | 0.903 | 0.65, 1.93 | - | - | - | - | 35 | 26 | 0.909 | 0.65, 1.93 | 35 | 27 | 1.000 | 0.63, 1.85 |
| *LIMK1* | 33 | 27 | 1.000 | 0.59, 1.75 | 0 | 1 | 0.905 | 0.00, 32.26 | 33 | 28 | 1.000 | 0.57, 1.68 | 39 | 30 | 1.000 | 0.65, 1.80 |
| *NTRK2* | 10 | 6 | 0.903 | 0.45, 4.62 | - | - | - | - | 10 | 6 | 0.909 | 0.45, 4.62 | 11 | 7 | 1.000 | 0.46, 3.96 |
| *PIK3CA* | 18 | 13 | 0.971 | 0.53, 2.55 | - | - | - | - | 18 | 13 | 0.971 | 0.53, 2.55 | 21 | 17 | 1.000 | 0.51, 2.07 |
| *PIK3CB* | 36 | 26 | 0.903 | 0.67, 1.98 | - | - | - | - | 36 | 26 | 0.909 | 0.67, 1.98 | 39 | 27 | 1.000 | 0.71, 2.04 |
| *AKT1* | 3 | 4 | 0.903 | 0.09, 3.67 | - | - | - | - | 3 | 4 | 0.909 | 0.09, 3.67 | 3 | 4 | 1.000 | 0.09, 3.67 |
| *MTOR* | 68 | 31 | 0.099 | 1.18, 2.90 | 48 | 38 | 1.000 | 0.67, 1.65 | 116 | 69 | 0.254 | 1.03, 1.92 | 71 | 32 | **0.063** | **1.20, 2.91** |

Data were described as the number of variants (MAF<0.01). The number of Case is the total number of alleles carried with all patients.The number of ctrl is the total number of alleles carried with all healthy controls. p is the P value of Fisher test after FDR correction, LL and UL is respectively the lower limit and upper limit of 95% confidence intervals in odds ratio (OR). Missense, missense variant; Dmis, damaging missense; LoF, loss-of-function variant; Deleterious, Dmis+LoF; MAF, minor allele frequency.

**Supplementary Table 6 The Fisher test of Nogo-related genes rare variants in the sLOPD cohort**

| Gene | sLOPD cohort | | | | | | | | | | | | | | | |
| --- | --- | --- | --- | --- | --- | --- | --- | --- | --- | --- | --- | --- | --- | --- | --- | --- |
|  | Dmis | | | | Lof | | | | Deleterious | | | | Missense | | | |
|  | Case | Ctrl | p | LL, UL | Case | Ctrl | p | LL, UL | Case | Ctrl | p | LL, UL | Case | Ctrl | p | LL, UL |
| *RTN4* | 56 | 33 | 1.000 | 0.71, 1.76 | 3 | 0 | 0.789 | 0.27, - | 59 | 33 | 1.000 | 0.75, 1.85 | 69 | 41 | 1.000 | 0.73, 1.66 |
| *RTN4R* | 24 | 12 | 1.000 | 0.63, 2.87 | - | - | - | - | 24 | 12 | 1.000 | 0.63, 2.87 | 52 | 32 | 1.000 | 0.67, 1.71 |
| *LINGO1* | 6 | 4 | 1.000 | 0.23, 4.72 | - | - | - | - | 6 | 4 | 1.000 | 0.23, 4.72 | 8 | 7 | 1.000 | 0.24, 2.41 |
| *NGFR* | 21 | 14 | 1.000 | 0.47, 2.08 | - | - | - | - | 21 | 14 | 1.000 | 0.47, 2.08 | 60 | 37 | 1.000 | 0.69, 1.64 |
| *LILRB1* | 10 | 9 | 1.000 | 0.26, 2.02 | 7 | 2 | 0.795 | 0.43, 22.55 | 17 | 11 | 1.000 | 0.44, 2.38 | 77 | 46 | 1.000 | 0.75, 1.62 |
| *PLXNA2* | 76 | 46 | 1.000 | 0.74, 1.60 | 0 | 1 | 0.789 | 0.00, 25.42 | 76 | 47 | 1.000 | 0.72, 1.56 | 82 | 49 | 1.000 | 0.75, 1.60 |
| *DPYSL2* | 24 | 7 | 1.000 | 0.94, 6.17 | - | - | - | - | 24 | 7 | 1.000 | 0.94, 6.17 | 28 | 11 | 1.000 | 0.80, 3.71 |
| *ITGAV* | 24 | 20 | 1.000 | 0.41, 1.49 | - | - | - | - | 24 | 20 | 1.000 | 0.41, 1.49 | 28 | 22 | 1.000 | 0.46, 1.52 |
| *S1PR2* | 44 | 22 | 1.000 | 0.76, 2.30 | - | - | - | - | 44 | 22 | 1.000 | 0.76, 2.30 | 44 | 23 | 1.000 | 0.74, 2.17 |
| *RHOA* | - | - | - | - | - | - | - | - | - | - | - | - | 2 | 4 | 1.000 | 0.03, 2.27 |
| *ROCK1* | 20 | 21 | 1.000 | 0.32, 1.20 | 1 | 0 | 1.000 | 0.02, - | 21 | 21 | 1.000 | 0.34, 1.25 | 22 | 21 | 1.000 | 0.36, 1.31 |
| *LIMK1* | 24 | 18 | 1.000 | 0.45, 1.70 | 0 | 1 | 0.789 | 0.00, 25.42 | 24 | 19 | 1.000 | 0.43, 1.59 | 27 | 20 | 1.000 | 0.47, 1.66 |
| *NTRK2* | 11 | 6 | 1.000 | 0.40, 3.94 | 3 | 5 | 0.789 | 0.06, 2.01 | 14 | 11 | 1.000 | 0.35, 2.02 | 11 | 7 | 1.000 | 0.36, 3.12 |
| *PIK3CA* | 10 | 8 | 1.000 | 0.29, 2.38 | - | - | - | - | 10 | 8 | 1.000 | 0.29, 2.38 | 12 | 9 | 1.000 | 0.34, 2.34 |
| *PIK3CB* | 21 | 14 | 1.000 | 0.47, 2.08 | - | - | - | - | 21 | 14 | 1.000 | 0.47, 2.08 | 21 | 15 | 1.000 | 0.45, 1.91 |
| *AKT1* | 6 | 3 | 1.000 | 0.28, 8.07 | 1 | 0 | 1.000 | 0.02, - | 7 | 3 | 1.000 | 0.35, 9.13 | 6 | 3 | 1.000 | 0.28, 8.07 |
| *MTOR* | 39 | 23 | 1.000 | 0.64, 1.95 | 1 | 0 | 1.000 | 0.02, - | 40 | 23 | 1.000 | 0.66, 1.99 | 40 | 25 | 1.000 | 0.62, 1.80 |

Data were described as the number of variants (MAF<0.01). The number of Case is the total number of alleles carried with all patients.The number of ctrl is the total number of alleles carried with all healthy controls. p is the P value of Fisher test after FDR correction, LL and UL is respectively the lower limit and upper limit of 95% confidence intervals in odds ratio (OR). Missense, missense variant; Dmis, damaging missense; LoF, loss-of-function variant; Deleterious, Dmis+LoF; MAF, minor allele frequency.

**Supplementary Table 7 the rare variants of Nogo-related genes identified in the sEOPD & FPD cohort**

| **Gene** | **Chr** | **Position** | **Ref** | **Alt** | **NM_number** | **AAChangerefGene** | **Consequence** | **gnomAD_exome_EAS** | **gnomAD_genome_EAS** | **ExAC_EAS** | **CADD** | **WES**  **(Case)** | **WES**  **(Control)** |
| --- | --- | --- | --- | --- | --- | --- | --- | --- | --- | --- | --- | --- | --- |
| MTOR | chr1 | 11319445 | C | T | NM_004958 | c.22G>A:p.A8T | Missense | 6.52E-05 | - | 0 | 23.3 | 0 | 1 |
| MTOR | chr1 | 11317004 | G | A | NM_004958 | c.490C>T:p.R164W | Missense | - | - | - | 26.9 | 0 | 1 |
| MTOR | chr1 | 11316998 | G | A | NM_004958 | c.496C>T:p.H166Y | Missense | - | 0.0006 | - | 24 | 1 | 2 |
| MTOR | chr1 | 11316072 | T | C | NM_004958 | c.682A>G:p.M228V | Missense | - | - | - | 12.13 | 1 | 0 |
| MTOR | chr1 | 11313946 | C | T | NM_004958 | c.790G>A:p.A264T | Missense | 5.80E-05 | - | - | 23.1 | 5 | 1 |
| MTOR | chr1 | 11186751 | G | A | NM_004958 | c.6454C>T:p.R2152C | Missense | 0 | - | 0 | 24.5 | 1 | 0 |
| MTOR | chr1 | 11308082 | T | C | NM_004958 | c.910A>G:p.M304V | Missense | 5.80E-05 | - | - | 7.934 | 0 | 1 |
| MTOR | chr1 | 11307733 | T | C | NM_004958 | c.1174A>G:p.I392V | Missense | 0.0002 | - | 0.0003 | 10.14 | 2 | 0 |
| MTOR | chr1 | 11307706 | C | T | NM_004958 | c.1201G>A:p.A401T | Missense | 5.80E-05 | 0 | 0.0001 | 26.3 | 0 | 1 |
| MTOR | chr1 | 11307691 | C | T | NM_004958 | c.1216G>A:p.A406T | Missense | - | - | - | 20.7 | 1 | 1 |
| MTOR | chr1 | 11303276 | G | A | NM_004958 | c.1307C>T:p.A436V | Missense | - | - | - | 32 | 0 | 1 |
| MTOR | chr1 | 11186800 | G | C | NM_004958 | c.6405C>G:p.D2135E | Missense | - | - | - | 26.9 | 1 | 0 |
| MTOR | chr1 | 11189856 | C | T | NM_004958 | c.5653G>A:p.V1885I | Missense | 5.80E-05 | 0 | 0.0001 | 22.1 | 1 | 0 |
| MTOR | chr1 | 11189882 | G | A | NM_004958 | c.5627C>T:p.T1876I | Missense | 0.0002 | - | 0.0001 | 23.3 | 1 | 0 |
| MTOR | chr1 | 11190639 | C | T | NM_004958 | c.5560G>A:p.E1854K | Missense | 0 | - | 0 | 22.9 | 2 | 0 |
| MTOR | chr1 | 11190698 | G | A | NM_004958 | c.5501C>T:p.T1834M | Missense | 0 | 0 | 0 | 22.8 | 1 | 0 |
| MTOR | chr1 | 11190765 | C | T | NM_004958 | c.5434G>A:p.D1812N | Missense | 0 | - | 0 | 22.4 | 2 | 0 |
| MTOR | chr1 | 11190767 | C | T | NM_004958 | c.5432G>A:p.R1811H | Missense | 0 | - | 0 | 31 | 1 | 0 |
| MTOR | chr1 | 11292515 | G | C | NM_004958 | c.2492C>G:p.S831C | Missense | - | - | - | 24.8 | 0 | 1 |
| MTOR | chr1 | 11194424 | G | A | NM_004958 | c.5230C>T:p.H1744Y | Missense | 0.0002 | - | 0.0001 | 18.22 | 1 | 0 |
| MTOR | chr1 | 11273472 | C | T | NM_004958 | c.3269G>A:p.R1090H | Missense | 5.81E-05 | - | 0.0001 | 35 | 0 | 1 |
| MTOR | chr1 | 11217266 | T | C | NM_004958 | c.4412A>G:p.K1471R | Missense | - | - | - | 22.7 | 1 | 0 |
| MTOR | chr1 | 11272529 | G | A | NM_004958 | c.3401C>T:p.A1134V | Missense | 0.0044 | 0.0056 | 0.0049 | 16.89 | 22 | 10 |
| MTOR | chr1 | 11217336 | T | G | NM_004958 | c.4342A>C:p.T1448P | Missense | - | - | - | 22.9 | 1 | 0 |
| MTOR | chr1 | 11264688 | G | T | NM_004958 | c.3874C>A:p.L1292M | Missense | - | - | - | 27.8 | 1 | 0 |
| MTOR | chr1 | 11269506 | G | C | NM_004958 | c.3664C>G:p.L1222V | Missense | - | 0 | - | 20.2 | 0 | 1 |
| MTOR | chr1 | 11270876 | C | G | NM_004958 | c.3649G>C:p.V1217L | Missense | - | 0 | - | 13.12 | 1 | 0 |
| MTOR | chr1 | 11270894 | C | T | NM_004958 | c.3631G>A:p.V1211M | Missense | - | - | - | 22.4 | 1 | 0 |
| MTOR | chr1 | 11217269 | T | C | NM_004958 | c.4409A>G:p.N1470S | Missense | - | 0 | - | 13.96 | 0 | 1 |
| MTOR | chr1 | 11272380 | G | C | NM_004958 | c.3550C>G:p.L1184V | Missense | 0.0005 | - | 0.0003 | 23.2 | 2 | 0 |
| MTOR | chr1 | 11217239 | C | T | NM_004958 | c.4439G>A:p.R1480H | Missense | 0 | 0 | 0 | 31 | 0 | 1 |
| MTOR | chr1 | 11272478 | T | C | NM_004958 | c.3452A>G:p.Y1151C | Missense | 0 | 0 | 0 | 24.8 | 1 | 0 |
| MTOR | chr1 | 11272898 | A | G | NM_004958 | c.3353T>C:p.I1118T | Missense | - | - | - | 23.9 | 1 | 0 |
| MTOR | chr1 | 11210265 | C | A | NM_004958 | c.4488G>T:p.Q1496H | Missense | - | - | - | 25.2 | 0 | 1 |
| MTOR | chr1 | 11273494 | T | C | NM_004958 | c.3247A>G:p.M1083V | Missense | 0.0013 | 0.0006 | 0.0018 | 16.56 | 3 | 0 |
| MTOR | chr1 | 11199444 | G | A | NM_004958 | c.5047C>T:p.R1683W | Missense | 5.81E-05 | 0.0006 | - | 34 | 0 | 1 |
| MTOR | chr1 | 11288748 | C | T | NM_004958 | c.3007G>A:p.V1003I | Missense | - | - | - | 23.8 | 1 | 0 |
| MTOR | chr1 | 11288778 | C | T | NM_004958 | c.2977G>A:p.V993I | Missense | 5.80E-05 | - | - | 17.13 | 1 | 0 |
| MTOR | chr1 | 11190706 | G | - | NM_004958 | c.5493delC:p.A1832Pfs*46 | Frameshift deletion | - | - | - | - | 24 | 19 |
| MTOR | chr1 | 11190698 | G | - | NM_004958 | c.5501delC:p.T1834Rfs*44 | Frameshift deletion | - | - | - | - | 24 | 19 |
| MTOR | chr1 | 11190695 | G | A | NM_004958 | c.5504C>T:p.A1835V | Missense | - | - | - | 22.3 | 0 | 1 |
| MTOR | chr1 | 11190693 | C | T | NM_004958 | c.5506G>A:p.A1836T | Missense | 0.0003 | 0.0012 | 0.0005 | 12.62 | 4 | 1 |
| MTOR | chr1 | 11297977 | C | A | NM_004958 | c.2131G>T:p.A711S | Missense | - | - | - | 33 | 2 | 0 |
| MTOR | chr1 | 11190630 | C | T | NM_004958 | c.5569G>A:p.E1857K | Missense | 0 | - | 0 | 23.8 | 0 | 2 |
| MTOR | chr1 | 11298534 | C | T | NM_004958 | c.1927G>A:p.V643M | Missense | 0 | - | 0 | 23.6 | 1 | 0 |
| MTOR | chr1 | 11190590 | G | C | NM_004958 | c.5609C>G:p.T1870S | Missense | 0.0006 | 0.0006 | 0.0003 | 14.82 | 1 | 1 |
| MTOR | chr1 | 11298542 | T | C | NM_004958 | c.1919A>G:p.H640R | Missense | 0.0004 | - | 0.0003 | 13.37 | 1 | 0 |
| MTOR | chr1 | 11298578 | C | T | NM_004958 | c.1883G>A:p.R628H | Missense | 0 | - | 0 | 23.3 | 1 | 0 |
| MTOR | chr1 | 11300569 | C | T | NM_004958 | c.1577G>A:p.R526H | Missense | 0 | 0.0006 | - | 24.7 | 1 | 0 |
| MTOR | chr1 | 11319454 | C | T | NM_004958 | c.13G>A:p.G5R | Missense | 0 | - | 0 | 22.9 | 1 | 0 |
| MTOR | chr1 | 11303220 | C | T | NM_004958 | c.1363G>A:p.V455M | Missense | 0 | 0.0006 | 0 | 25.9 | 2 | 0 |
| MTOR | chr1 | 11181361 | T | C | NM_004958 | c.6875A>G:p.N2292S | Missense | 0 | 0 | 0 | 17.54 | 0 | 1 |
| PLXNA2 | chr1 | 208200597 | A | C | NM_025179 | c.5676T>G:p.I1892M | Missense | 5.80E-05 | - | 0.0001 | 4.581 | 1 | 0 |
| PLXNA2 | chr1 | 208200640 | C | T | NM_025179 | c.5633G>A:p.R1878Q | Missense | 0 | 0 | 0 | 34 | 0 | 1 |
| PLXNA2 | chr1 | 208200649 | C | T | NM_025179 | c.5624G>A:p.R1875Q | Missense | 0.001 | 0.0006 | 0.001 | 27.2 | 6 | 8 |
| PLXNA2 | chr1 | 208200677 | C | G | NM_025179 | c.5596G>C:p.G1866R | Missense | 5.80E-05 | - | - | 23.5 | 2 | 0 |
| PLXNA2 | chr1 | 208201419 | C | T | NM_025179 | c.5524G>A:p.V1842M | Missense | 0.0001 | 0 | 0.0001 | 23.3 | 0 | 1 |
| PLXNA2 | chr1 | 208201440 | C | T | NM_025179 | c.5503G>A:p.E1835K | Missense | 0 | - | - | 35 | 0 | 1 |
| PLXNA2 | chr1 | 208202188 | T | C | NM_025179 | c.5425A>G:p.S1809G | Missense | - | - | - | 20.5 | 0 | 1 |
| PLXNA2 | chr1 | 208202203 | T | A | NM_025179 | c.5410A>T:p.I1804F | Missense | - | - | - | 33 | 0 | 1 |
| PLXNA2 | chr1 | 208202313 | G | A | NM_025179 | c.5300C>T:p.T1767M | Missense | - | 0.0006 | - | 34 | 0 | 1 |
| PLXNA2 | chr1 | 208205047 | G | A | NM_025179 | c.5113C>T:p.R1705W | Missense | 0 | 0 | 0 | 35 | 1 | 0 |
| PLXNA2 | chr1 | 208206804 | G | A | NM_025179 | c.4915C>T:p.P1639S | Missense | - | - | - | 29.6 | 0 | 1 |
| PLXNA2 | chr1 | 208206836 | G | C | NM_025179 | c.4883C>G:p.T1628R | Missense | - | - | - | 33 | 1 | 0 |
| PLXNA2 | chr1 | 208207840 | T | C | NM_025179 | c.4862A>G:p.Y1621C | Missense | 0 | - | - | 28 | 0 | 1 |
| PLXNA2 | chr1 | 208207855 | G | A | NM_025179 | c.4847C>T:p.T1616M | Missense | 0 | - | 0 | 28 | 1 | 0 |
| PLXNA2 | chr1 | 208211790 | C | T | NM_025179 | c.4690G>A:p.V1564M | Missense | 0 | 0 | 0 | 34 | 1 | 0 |
| PLXNA2 | chr1 | 208211792 | A | G | NM_025179 | c.4688T>C:p.V1563A | Missense | - | - | - | 33 | 1 | 0 |
| PLXNA2 | chr1 | 208211799 | C | A | NM_025179 | c.4681G>T:p.A1561S | Missense | 0.0001 | - | 0.0001 | 29.1 | 3 | 4 |
| PLXNA2 | chr1 | 208211813 | C | T | NM_025179 | c.4667G>A:p.R1556H | Missense | 5.80E-05 | 0.0006 | 0 | 35 | 1 | 0 |
| PLXNA2 | chr1 | 208212178 | A | G | NM_025179 | c.4652T>C:p.M1551T | Missense | 5.80E-05 | - | 0.0001 | 27.7 | 0 | 1 |
| PLXNA2 | chr1 | 208212220 | T | C | NM_025179 | c.4610A>G:p.Y1537C | Missense | 0 | 0 | - | 26 | 1 | 0 |
| PLXNA2 | chr1 | 208212307 | T | G | NM_025179 | c.4523A>C:p.D1508A | Missense | - | - | - | 25.1 | 0 | 1 |
| PLXNA2 | chr1 | 208215573 | C | T | NM_025179 | c.4156G>A:p.V1386M | Missense | 0 | - | - | 34 | 1 | 0 |
| PLXNA2 | chr1 | 208215620 | C | T | NM_025179 | c.4109G>A:p.R1370H | Missense | 0 | 0 | 0 | 29.4 | 1 | 0 |
| PLXNA2 | chr1 | 208215689 | T | C | NM_025179 | c.4040A>G:p.H1347R | Missense | - | - | - | 6.131 | 1 | 0 |
| PLXNA2 | chr1 | 208215699 | C | T | NM_025179 | c.4030G>A:p.G1344R | Missense | 0 | - | 0 | 22.7 | 0 | 1 |
| PLXNA2 | chr1 | 208216423 | C | T | NM_025179 | c.4000G>A:p.V1334I | Missense | 0.0002 | - | 0.0001 | 29.8 | 0 | 1 |
| PLXNA2 | chr1 | 208216477 | C | T | NM_025179 | c.3946G>A:p.D1316N | Missense | - | - | - | 34 | 1 | 0 |
| PLXNA2 | chr1 | 208217991 | C | T | NM_025179 | c.3736G>A:p.G1246S | Missense | 0 | - | 0 | 29.5 | 1 | 0 |
| PLXNA2 | chr1 | 208218017 | G | T | NM_025179 | c.3710C>A:p.P1237Q | Missense | - | - | - | 29.3 | 0 | 1 |
| PLXNA2 | chr1 | 208218042 | T | C | NM_025179 | c.3685A>G:p.I1229V | Missense | 0 | - | 0 | 9.951 | 1 | 0 |
| PLXNA2 | chr1 | 208218068 | A | G | NM_025179 | c.3659T>C:p.V1220A | Missense | - | - | - | 17.81 | 0 | 1 |
| PLXNA2 | chr1 | 208219257 | AA | - | NM_025179 | c.3460_3461del:p.L1154Gfs*41 | Frameshift deletion | - | - | - | - | 0 | 1 |
| PLXNA2 | chr1 | 208219337 | T | G | NM_025179 | c.3381A>C:p.Q1127H | Missense | - | - | - | 23.4 | 0 | 1 |
| PLXNA2 | chr1 | 208224386 | G | A | NM_025179 | c.3223C>T:p.R1075X | Stopgain | 0 | - | 0 | 48 | 0 | 1 |
| PLXNA2 | chr1 | 208224650 | T | C | NM_025179 | c.3112A>G:p.I1038V | Missense | 0 | - | 0 | 22.5 | 1 | 0 |
| PLXNA2 | chr1 | 208224685 | C | T | NM_025179 | c.3077G>A:p.R1026Q | Missense | 0 | - | 0 | 32 | 0 | 2 |
| PLXNA2 | chr1 | 208224737 | G | T | NM_025179 | c.3025C>A:p.P1009T | Missense | 0.0005 | 0.0006 | 0.0005 | 17.8 | 0 | 1 |
| PLXNA2 | chr1 | 208227804 | C | G | NM_025179 | c.2818G>C:p.E940Q | Missense | - | - | - | 26.7 | 0 | 1 |
| PLXNA2 | chr1 | 208227830 | C | T | NM_025179 | c.2792G>A:p.R931H | Missense | 0.0021 | 0.0037 | 0.0022 | 22.8 | 13 | 12 |
| PLXNA2 | chr1 | 208227831 | G | A | NM_025179 | c.2791C>T:p.R931C | Missense | 0.0008 | 0.0006 | 0.0008 | 32 | 2 | 4 |
| PLXNA2 | chr1 | 208227855 | C | T | NM_025179 | c.2767G>A:p.V923M | Missense | 0.0002 | - | 0.0001 | 17.77 | 3 | 1 |
| PLXNA2 | chr1 | 208227879 | C | T | NM_025179 | c.2743G>A:p.V915I | Missense | - | - | - | 26.8 | 0 | 1 |
| PLXNA2 | chr1 | 208234173 | C | T | NM_025179 | c.2596G>A:p.V866M | Missense | 0 | - | - | 22.6 | 1 | 0 |
| PLXNA2 | chr1 | 208252702 | C | T | NM_025179 | c.2489G>A:p.R830H | Missense | 0 | - | 0 | 23.5 | 1 | 0 |
| PLXNA2 | chr1 | 208252706 | C | T | NM_025179 | c.2485G>A:p.E829K | Missense | 0.0002 | - | 0.0002 | 24.1 | 0 | 1 |
| PLXNA2 | chr1 | 208252736 | G | A | NM_025179 | c.2455C>T:p.R819W | Missense | 0.0008 | - | 0.0008 | 26.4 | 1 | 3 |
| PLXNA2 | chr1 | 208252768 | C | T | NM_025179 | c.2423G>A:p.R808Q | Missense | 0.0002 | - | 0.0004 | 35 | 0 | 1 |
| PLXNA2 | chr1 | 208255837 | A | G | NM_025179 | c.2315T>C:p.M772T | Missense | - | - | - | 13.6 | 1 | 0 |
| PLXNA2 | chr1 | 208257814 | A | G | NM_025179 | c.2209T>C:p.Y737H | Missense | - | - | - | 24.9 | 1 | 0 |
| PLXNA2 | chr1 | 208257852 | G | A | NM_025179 | c.2171C>T:p.A724V | Missense | 0 | - | 0 | 18.12 | 0 | 2 |
| PLXNA2 | chr1 | 208257869 | C | A | NM_025179 | c.2154G>T:p.K718N | Missense | - | - | - | 17.39 | 0 | 1 |
| PLXNA2 | chr1 | 208266217 | G | A | NM_025179 | c.2011C>T:p.R671C | Missense | 0 | - | - | 35 | 1 | 0 |
| PLXNA2 | chr1 | 208269447 | G | C | NM_025179 | c.1909C>G:p.L637V | Missense | - | - | - | 23.3 | 1 | 0 |
| PLXNA2 | chr1 | 208270124 | G | C | NM_025179 | c.1836C>G:p.I612M | Missense | 5.80E-05 | - | 0.0001 | 15.66 | 0 | 1 |
| PLXNA2 | chr1 | 208270138 | C | T | NM_025179 | c.1822G>A:p.G608R | Missense | 0.0005 | - | 0.0001 | 28.5 | 1 | 3 |
| PLXNA2 | chr1 | 208270191 | G | A | NM_025179 | c.1769C>T:p.A590V | Missense | 0 | 0 | 0 | 21.2 | 1 | 0 |
| PLXNA2 | chr1 | 208272216 | A | G | NM_025179 | c.1706T>C:p.I569T | Missense | - | - | - | 24.6 | 0 | 1 |
| PLXNA2 | chr1 | 208272219 | C | T | NM_025179 | c.1703G>A:p.S568N | Missense | 0.0003 | - | - | 11.43 | 4 | 0 |
| PLXNA2 | chr1 | 208272229 | G | A | NM_025179 | c.1693C>T:p.H565Y | Missense | - | - | - | 0.079 | 0 | 1 |
| PLXNA2 | chr1 | 208315702 | C | T | NM_025179 | c.1478G>A:p.R493H | Missense | 0 | 0 | 0 | 23 | 0 | 1 |
| PLXNA2 | chr1 | 208315799 | C | T | NM_025179 | c.1381G>A:p.D461N | Missense | 0 | 0 | 0 | 23.5 | 0 | 1 |
| PLXNA2 | chr1 | 208315804 | C | T | NM_025179 | c.1376G>A:p.R459Q | Missense | 5.95E-05 | - | - | 23.3 | 1 | 0 |
| PLXNA2 | chr1 | 208383636 | T | C | NM_025179 | c.1360A>G:p.K454E | Missense | - | - | - | 21.7 | 0 | 1 |
| PLXNA2 | chr1 | 208383693 | C | T | NM_025179 | c.1303G>A:p.V435M | Missense | - | - | - | 31 | 1 | 0 |
| PLXNA2 | chr1 | 208383704 | C | T | NM_025179 | c.1292G>A:p.R431H | Missense | 0 | - | 0 | 27.6 | 1 | 0 |
| PLXNA2 | chr1 | 208383731 | A | G | NM_025179 | c.1265T>C:p.L422P | Missense | - | - | - | 23.9 | 1 | 0 |
| PLXNA2 | chr1 | 208383752 | C | T | NM_025179 | c.1244G>A:p.G415D | Missense | 5.80E-05 | - | 0.0001 | 31 | 0 | 1 |
| PLXNA2 | chr1 | 208390097 | C | T | NM_025179 | c.1171G>A:p.V391I | Missense | 0 | - | 0 | 23.7 | 0 | 1 |
| PLXNA2 | chr1 | 208390101 | C | G | NM_025179 | c.1167G>C:p.K389N | Missense | - | - | - | 23.2 | 0 | 1 |
| PLXNA2 | chr1 | 208390136 | C | T | NM_025179 | c.1132G>A:p.E378K | Missense | 0 | - | 0 | 26.7 | 0 | 1 |
| PLXNA2 | chr1 | 208390186 | C | T | NM_025179 | c.1082G>A:p.R361Q | Missense | 0.0004 | 0.0006 | 0.0001 | 21.1 | 5 | 4 |
| PLXNA2 | chr1 | 208390199 | C | T | NM_025179 | c.1069G>A:p.A357T | Missense | - | - | - | 23.6 | 0 | 1 |
| PLXNA2 | chr1 | 208390358 | C | T | NM_025179 | c.910G>A:p.G304R | Missense | 0.0003 | 0 | 0.0006 | 24.2 | 0 | 2 |
| PLXNA2 | chr1 | 208390398 | G | T | NM_025179 | c.870C>A:p.F290L | Missense | - | - | - | 25.6 | 1 | 0 |
| PLXNA2 | chr1 | 208390457 | C | T | NM_025179 | c.811G>A:p.A271T | Missense | 5.80E-05 | - | 0.0001 | 23.6 | 0 | 1 |
| PLXNA2 | chr1 | 208390748 | G | A | NM_025179 | c.520C>T:p.R174C | Missense | 0.0007 | 0.0012 | 0.0009 | 25.6 | 1 | 0 |
| PLXNA2 | chr1 | 208390764 | C | T | NM_025179 | c.504G>A:p.M168I | Missense | - | - | - | 23.6 | 0 | 1 |
| PLXNA2 | chr1 | 208390903 | G | T | NM_025179 | c.365C>A:p.S122Y | Missense | 5.80E-05 | 0.0012 | 0 | 23.4 | 0 | 1 |
| PLXNA2 | chr1 | 208390951 | A | G | NM_025179 | c.317T>C:p.V106A | Missense | - | 0 | - | 1.19 | 0 | 1 |
| PLXNA2 | chr1 | 208390957 | C | T | NM_025179 | c.311G>A:p.S104N | Missense | - | - | - | 9.689 | 1 | 0 |
| PLXNA2 | chr1 | 208391023 | G | A | NM_025179 | c.245C>T:p.A82V | Missense | 0.0003 | - | 0.0006 | 20.6 | 2 | 1 |
| PLXNA2 | chr1 | 208391027 | C | G | NM_025179 | c.241G>C:p.V81L | Missense | - | - | - | 23.3 | 3 | 0 |
| PLXNA2 | chr1 | 208391063 | G | A | NM_025179 | c.205C>T:p.R69W | Missense | 0 | - | - | 31 | 0 | 1 |
| PLXNA2 | chr1 | 208391124 | C | G | NM_025179 | c.144G>C:p.W48C | Missense | - | - | - | 25 | 1 | 0 |
| PLXNA2 | chr1 | 208391146 | A | - | NM_025179 | c.122delT:p.F41Sfs*12 | Frameshift deletion | - | - | - | - | 0 | 1 |
| PLXNA2 | chr1 | 208391192 | C | A | NM_025179 | c.76G>T:p.V26L | Missense | 0.0006 | 0.0012 | 0.0006 | 9.567 | 2 | 0 |
| PLXNA2 | chr1 | 208391236 | A | G | NM_025179 | c.32T>C:p.L11P | Missense | 0.0012 | 0.0019 | 0.0011 | 0.001 | 5 | 9 |
| RTN4 | chr2 | 55200306 | G | A | NM_020532 | c.3565C>T:p.R1189C | Missense | 0.0009 | 0.0006 | 0.0007 | 28.4 | 0 | 1 |
| RTN4 | chr2 | 55200703 | C | T | NM_020532 | c.3532G>A:p.A1178T | Missense | - | - | - | 27.3 | 2 | 0 |
| RTN4 | chr2 | 55200706 | T | C | NM_020532 | c.3529A>G:p.M1177V | Missense | 5.80E-05 | - | 0.0001 | 14.1 | 1 | 0 |
| RTN4 | chr2 | 55200720 | TTC | - | NM_020532 | c.3513_3515del:p.K1171del | NonFrameshift deletion | - | - | - | - | 1 | 0 |
| RTN4 | chr2 | 55200727 | T | G | NM_020532 | c.3508A>C:p.N1170H | Missense | - | - | - | 26.9 | 0 | 1 |
| RTN4 | chr2 | 55200964 | C | T | NM_020532 | c.3470G>A:p.R1157Q | Missense | 5.81E-05 | - | - | 35 | 1 | 0 |
| RTN4 | chr2 | 55200966 | T | G | NM_020532 | c.3468A>C:p.E1156D | Missense | 5.81E-05 | - | - | 28.8 | 1 | 0 |
| RTN4 | chr2 | 55209706 | G | A | NM_020532 | c.3305C>T:p.T1102M | Missense | 0.0009 | 0.0012 | 0.0007 | 32 | 4 | 3 |
| RTN4 | chr2 | 55209718 | T | C | NM_020532 | c.3293A>G:p.H1098R | Missense | 5.80E-05 | - | 0.0001 | 27 | 0 | 3 |
| RTN4 | chr2 | 55209722 | - | CTT | NM_020532 | c.3288_3289insAAG:p.L1096_G1097insK | NonFrameshift deletion | - | - | - | - | 1 | 0 |
| RTN4 | chr2 | 55209764 | T | C | NM_020532 | c.3247A>G:p.I1083V | Missense | 0.0004 | - | 0.0001 | 4.276 | 3 | 0 |
| RTN4 | chr2 | 55214692 | G | C | NM_020532 | c.3156C>G:p.I1052M | Missense | 0.0004 | - | 0.0001 | 27.6 | 5 | 1 |
| RTN4 | chr2 | 55214712 | C | T | NM_020532 | c.3136G>A:p.A1046T | Missense | - | - | - | 34 | 1 | 0 |
| RTN4 | chr2 | 55252447 | C | T | NM_020532 | c.2788G>A:p.E930K | Missense | 0.0038 | 0.0025 | 0.0035 | 23.4 | 19 | 11 |
| RTN4 | chr2 | 55252486 | G | A | NM_020532 | c.2749C>T:p.H917Y | Missense | - | - | - | 22.2 | 0 | 1 |
| RTN4 | chr2 | 55252537 | T | G | NM_020532 | c.2698A>C:p.I900L | Missense | - | - | - | 8.077 | 1 | 0 |
| RTN4 | chr2 | 55252552 | A | G | NM_020532 | c.2683T>C:p.S895P | Missense | - | - | - | 15.33 | 1 | 0 |
| RTN4 | chr2 | 55252633 | T | C | NM_020532 | c.2602A>G:p.I868V | Missense | 5.80E-05 | - | 0.0001 | 13.72 | 0 | 1 |
| RTN4 | chr2 | 55252699 | A | G | NM_020532 | c.2536T>C:p.F846L | Missense | - | - | - | 0.48 | 0 | 1 |
| RTN4 | chr2 | 55252734 | T | A | NM_020532 | c.2501A>T:p.E834V | Missense | 0.0001 | 0 | 0.0002 | 25.7 | 2 | 1 |
| RTN4 | chr2 | 55252743 | T | C | NM_020532 | c.2492A>G:p.Q831R | Missense | - | - | - | 24.4 | 1 | 0 |
| RTN4 | chr2 | 55252762 | T | C | NM_020532 | c.2473A>G:p.K825E | Missense | - | - | - | 14.55 | 1 | 0 |
| RTN4 | chr2 | 55252804 | T | C | NM_020532 | c.2431A>G:p.K811E | Missense | 0 | - | 0 | 14.04 | 0 | 1 |
| RTN4 | chr2 | 55252821 | A | T | NM_020532 | c.2414T>A:p.L805H | Missense | - | - | - | 16.24 | 0 | 1 |
| RTN4 | chr2 | 55252832 | T | G | NM_020532 | c.2403A>C:p.E801D | Missense | - | - | - | 19.43 | 1 | 0 |
| RTN4 | chr2 | 55252874 | T | A | NM_020532 | c.2361A>T:p.K787N | Missense | 5.80E-05 | - | 0.0002 | 10.46 | 0 | 1 |
| RTN4 | chr2 | 55252944 | A | T | NM_020532 | c.2291T>A:p.V764E | Missense | 0.0001 | 0 | 0.0001 | 13.73 | 0 | 1 |
| RTN4 | chr2 | 55253004 | G | T | NM_020532 | c.2231C>A:p.P744Q | Missense | - | - | - | 25.1 | 1 | 1 |
| RTN4 | chr2 | 55253007 | T | G | NM_020532 | c.2228A>C:p.E743A | Missense | - | - | - | 25.3 | 0 | 1 |
| RTN4 | chr2 | 55253043 | T | A | NM_020532 | c.2192A>T:p.H731L | Missense | 5.80E-05 | - | - | 11.24 | 1 | 0 |
| RTN4 | chr2 | 55253044 | G | C | NM_020532 | c.2191C>G:p.H731D | Missense | - | - | - | 8.616 | 1 | 0 |
| RTN4 | chr2 | 55253157 | G | A | NM_020532 | c.2078C>T:p.P693L | Missense | 0.0002 | - | 0.0002 | 23.7 | 1 | 1 |
| RTN4 | chr2 | 55253290 | T | C | NM_020532 | c.1945A>G:p.I649V | Missense | 0.0004 | - | 0.0006 | 0.001 | 0 | 2 |
| RTN4 | chr2 | 55253305 | C | T | NM_020532 | c.1930G>A:p.V644I | Missense | 0 | - | 0 | 8.937 | 1 | 0 |
| RTN4 | chr2 | 55253409 | G | A | NM_020532 | c.1826C>T:p.P609L | Missense | 0.0004 | 0.0019 | 0.0005 | 26.8 | 2 | 4 |
| RTN4 | chr2 | 55253467 | G | A | NM_020532 | c.1768C>T:p.L590F | Missense | 5.80E-05 | 0 | 0.0001 | 20.9 | 1 | 0 |
| RTN4 | chr2 | 55253522 | A | C | NM_020532 | c.1713T>G:p.I571M | Missense | 5.80E-05 | - | - | 7.03 | 1 | 0 |
| RTN4 | chr2 | 55253608 | C | T | NM_020532 | c.1627G>A:p.V543M | Missense | 0.0002 | 0.0012 | 0.0005 | 11.55 | 2 | 0 |
| RTN4 | chr2 | 55253831 | - | TTA | NM_020532 | c.1403_1404insTAA:p.N468_P469insN | NonFrameshift deletion | - | 0.0006 | - | - | 0 | 1 |
| RTN4 | chr2 | 55253851 | T | C | NM_020532 | c.1384A>G:p.I462V | Missense | - | - | - | 23.3 | 1 | 0 |
| RTN4 | chr2 | 55253886 | G | A | NM_020532 | c.1349C>T:p.T450M | Missense | 0.0002 | 0 | 0.0002 | 27.4 | 1 | 3 |
| RTN4 | chr2 | 55253886 | G | T | NM_020532 | c.1349C>A:p.T450K | Missense | 0 | - | 0 | 27.9 | 0 | 1 |
| RTN4 | chr2 | 55253920 | C | G | NM_020532 | c.1315G>C:p.E439Q | Missense | - | - | - | 25.4 | 1 | 0 |
| RTN4 | chr2 | 55253933 | G | T | NM_020532 | c.1302C>A:p.H434Q | Missense | 0 | 0 | - | 0.585 | 0 | 1 |
| RTN4 | chr2 | 55254121 | T | G | NM_020532 | c.1114A>C:p.K372Q | Missense | - | - | - | 0.171 | 1 | 0 |
| RTN4 | chr2 | 55254190 | C | T | NM_020532 | c.1045G>A:p.A349T | Missense | - | - | - | 0.013 | 0 | 1 |
| RTN4 | chr2 | 55254279 | G | C | NM_020532 | c.956C>G:p.P319R | Missense | - | - | - | 3.412 | 1 | 0 |
| RTN4 | chr2 | 55254286 | C | G | NM_020532 | c.949G>C:p.A317P | Missense | 5.81E-05 | - | - | 0.038 | 1 | 1 |
| RTN4 | chr2 | 55254313 | G | T | NM_020532 | c.922C>A:p.P308T | Missense | 5.81E-05 | - | 0 | 11.16 | 1 | 0 |
| RTN4 | chr2 | 55254327 | G | A | NM_020532 | c.908C>T:p.S303L | Missense | 0 | 0 | 0 | 23.5 | 0 | 1 |
| RTN4 | chr2 | 55254372 | T | C | NM_020532 | c.863A>G:p.D288G | Missense | 0.003 | 0.0006 | 0.0014 | 12.86 | 6 | 9 |
| RTN4 | chr2 | 55254439 | G | C | NM_020532 | c.796C>G:p.Q266E | Missense | 5.80E-05 | - | - | 0.02 | 0 | 1 |
| RTN4 | chr2 | 55254445 | T | C | NM_020532 | c.790A>G:p.T264A | Missense | - | - | - | 0.001 | 1 | 0 |
| RTN4 | chr2 | 55254496 | T | C | NM_020532 | c.739A>G:p.K247E | Missense | - | - | - | 25.4 | 0 | 1 |
| RTN4 | chr2 | 55254514 | G | A | NM_020532 | c.721C>T:p.L241F | Missense | 0 | - | 0 | 22.8 | 0 | 1 |
| RTN4 | chr2 | 55254516 | G | A | NM_020532 | c.719C>T:p.P240L | Missense | - | - | - | 25.5 | 0 | 1 |
| RTN4 | chr2 | 55276955 | G | A | NM_020532 | c.482C>T:p.P161L | Missense | 0 | - | . | 23 | 1 | 0 |
| RTN4 | chr2 | 55276987 | G | T | NM_020532 | c.450C>A:p.S150R | Missense | - | - | - | 16.29 | 0 | 1 |
| RTN4 | chr2 | 55277061 | C | T | NM_020532 | c.376G>A:p.A126T | Missense | - | - | - | 11.76 | 1 | 0 |
| RTN4 | chr2 | 55277078 | G | A | NM_020532 | c.359C>T:p.P120L | Missense | - | - | - | 23.1 | 1 | 0 |
| RTN4 | chr2 | 55277130 | C | G | NM_020532 | c.307G>C:p.E103Q | Missense | - | - | - | 22.3 | 1 | 0 |
| RTN4 | chr2 | 55277133 | G | A | NM_020532 | c.304C>T:p.P102S | Missense | 0 | - | - | 23.6 | 1 | 1 |
| RTN4 | chr2 | 55277141 | G | A | NM_020532 | c.296C>T:p.P99L | Missense | 0.0011 | 0.0025 | 0.0044 | 11.4 | 6 | 4 |
| RTN4 | chr2 | 55277269 | CTCCAGCAC | - | NM_020532 | c.160_168del:p.V54_E56del | NonFrameshift deletion | 0.0002 | 0.0025 | 0.0006 | - | 1 | 0 |
| RTN4 | chr2 | 55277305 | - | TCCTCCTCTTCC | NM_020532 | c.131_132insGGAAGAGGAGGA:p.E43_D44insEEEE | NonFrameshift deletion | 0 | - | - | - | 0 | 1 |
| RTN4 | chr2 | 55277306 | T | A | NM_020532 | c.131A>T:p.D44V | Missense | - | - | - | 22.5 | 0 | 1 |
| RTN4 | chr2 | 55277307 | C | T | NM_020532 | c.130G>A:p.D44N | Missense | 0 | - | 0 | 23.2 | 0 | 1 |
| RTN4 | chr2 | 55277312 | TCTTCCTCCTCCTCT | - | NM_020532 | c.111_125del:p.E39_E43del | NonFrameshift deletion | - | - | - | - | 1 | 3 |
| RTN4 | chr2 | 55277314 | TTCC | - | NM_020532 | c.120_123del:p.E41Rfs*36 | Frameshift deletion | - | - | - | - | 1 | 3 |
| RTN4 | chr2 | 55277315 | TCC | - | NM_020532 | c.120_122del:p.E43del | NonFrameshift deletion | 0 | - | - | - | 0 | 1 |
| RTN4 | chr2 | 55277323 | CTCT | - | NM_020532 | c.111_114del:p.E38Rfs*39 | Frameshift deletion | - | - | - | - | 1 | 3 |
| RTN4 | chr2 | 55277406 | A | C | NM_020532 | c.31T>G:p.S11A | Missense | 0.0004 | 0.0006 | 0.0001 | 21.9 | 1 | 0 |
| ITGAV | chr2 | 187455214 | G | C | NM_001145000 | c.149G>C:p.G50A | Missense | 0.0001 | - | - | 25.8 | 2 | 0 |
| ITGAV | chr2 | 187455233 | C | G | NM_001145000 | c.168C>G:p.F56L | Missense | 0 | - | 0 | 26.4 | 1 | 0 |
| ITGAV | chr2 | 187465075 | C | T | NM_001144999 | c.14C>T:p.T5I | Missense | - | - | - | 2.357 | 2 | 1 |
| ITGAV | chr2 | 187465102 | T | G | NM_001144999 | c.41T>G:p.M14R | Missense | 9.76E-05 | - | - | 4.079 | 0 | 1 |
| ITGAV | chr2 | 187466849 | G | A | NM_001144999 | c.149G>A:p.R50Q | Missense | 0.0013 | 0.0012 | 0.0015 | 16.98 | 3 | 0 |
| ITGAV | chr2 | 187466873 | C | G | NM_001144999 | c.173C>G:p.A58G | Missense | - | - | - | 22.4 | 0 | 1 |
| ITGAV | chr2 | 187490314 | C | A | NM_001144999 | c.385C>A:p.Q129K | Missense | 0 | - | - | 11.57 | 1 | 0 |
| ITGAV | chr2 | 187495524 | A | G | NM_001144999 | c.386A>G:p.Q129R | Missense | - | - | - | 14.46 | 0 | 1 |
| ITGAV | chr2 | 187500841 | G | A | NM_001144999 | c.526G>A:p.V176I | Missense | 0.0009 | 0.0006 | 0.0008 | 2.901 | 2 | 2 |
| ITGAV | chr2 | 187500913 | A | G | NM_001144999 | c.598A>G:p.I200V | Missense | - | - | - | 13.26 | 1 | 0 |
| ITGAV | chr2 | 187505681 | A | G | NM_001144999 | c.805A>G:p.I269V | Missense | 5.97E-05 | - | 0 | 9.986 | 1 | 0 |
| ITGAV | chr2 | 187506129 | A | T | NM_001144999 | c.835A>T:p.I279F | Missense | 0.0007 | 0.0025 | 0.0002 | 26.8 | 1 | 5 |
| ITGAV | chr2 | 187506154 | G | A | NM_001144999 | c.860G>A:p.R287H | Missense | 0 | - | - | 34 | 1 | 2 |
| ITGAV | chr2 | 187506210 | G | A | NM_001144999 | c.916G>A:p.A306T | Missense | 0.0002 | 0.0006 | - | 15.53 | 0 | 4 |
| ITGAV | chr2 | 187506229 | C | T | NM_001144999 | c.935C>T:p.T312M | Missense | 5.80E-05 | 0 | - | 21.1 | 1 | 0 |
| ITGAV | chr2 | 187511418 | G | A | NM_001144999 | c.1027G>A:p.A343T | Missense | 0 | - | 0 | 33 | 0 | 1 |
| ITGAV | chr2 | 187511452 | A | G | NM_001144999 | c.1061A>G:p.K354R | Missense | 0.0002 | - | - | 8.813 | 0 | 1 |
| ITGAV | chr2 | 187511584 | T | C | NM_001144999 | c.1193T>C:p.I398T | Missense | - | - | 0 | 14.94 | 1 | 0 |
| ITGAV | chr2 | 187516797 | A | G | NM_001144999 | c.1348A>G:p.T450A | Missense | 0.0001 | 0.0006 | - | 20.8 | 0 | 1 |
| ITGAV | chr2 | 187519406 | A | G | NM_001144999 | c.1397A>G:p.D466G | Missense | 0.0004 | - | 0.0003 | 22.7 | 1 | 0 |
| ITGAV | chr2 | 187519408 | G | A | NM_001144999 | c.1399G>A:p.G467S | Missense | 0.0002 | - | 0.0002 | 31 | 1 | 1 |
| ITGAV | chr2 | 187521046 | G | A | NM_001144999 | c.1499G>A:p.S500N | Missense | - | - | - | 13.62 | 1 | 0 |
| ITGAV | chr2 | 187521057 | A | G | NM_001144999 | c.1510A>G:p.S504G | Missense | 0.0008 | 0.0006 | 0.001 | 9.346 | 6 | 4 |
| ITGAV | chr2 | 187521127 | G | A | NM_001144999 | c.1580G>A:p.R527Q | Missense | 0 | 0.0006 | 0 | 33 | 0 | 1 |
| ITGAV | chr2 | 187523776 | A | T | NM_001144999 | c.1593A>T:p.E531D | Missense | - | - | - | 24.3 | 2 | 0 |
| ITGAV | chr2 | 187523835 | C | T | NM_001144999 | c.1652C>T:p.T551I | Missense | - | - | - | 20.5 | 0 | 1 |
| ITGAV | chr2 | 187529241 | T | C | NM_001144999 | c.1808T>C:p.I603T | Missense | 0.0005 | 0.0006 | 0.0005 | 28.4 | 0 | 1 |
| ITGAV | chr2 | 187529253 | A | G | NM_001144999 | c.1820A>G:p.N607S | Missense | 5.80E-05 | - | - | 24.3 | 1 | 0 |
| ITGAV | chr2 | 187529298 | C | G | NM_001144999 | c.1865C>G:p.A622G | Missense | - | - | - | 25.8 | 1 | 0 |
| ITGAV | chr2 | 187529321 | T | G | NM_001144999 | c.1888T>G:p.S630A | Missense | - | - | - | 0.013 | 1 | 0 |
| ITGAV | chr2 | 187529871 | T | G | NM_001144999 | c.1954T>G:p.C652G | Missense | 5.85E-05 | - | 0.0001 | 29.8 | 1 | 0 |
| ITGAV | chr2 | 187529899 | G | T | NM_001144999 | c.1982G>T:p.R661L | Missense | - | - | - | 34 | 0 | 1 |
| ITGAV | chr2 | 187529940 | A | T | NM_001144999 | c.2023A>T:p.T675S | Missense | - | - | - | 24 | 1 | 0 |
| ITGAV | chr2 | 187531478 | T | A | NM_001144999 | c.2076T>A:p.D692E | Missense | - | - | - | 24.2 | 1 | 0 |
| ITGAV | chr2 | 187531908 | G | A | NM_001144999 | c.2140G>A:p.V714I | Missense | - | - | - | 21.3 | 0 | 1 |
| ITGAV | chr2 | 187532474 | G | A | NM_001144999 | c.2266G>A:p.V756I | Missense | 0.0003 | - | 0.0003 | 11.66 | 1 | 1 |
| ITGAV | chr2 | 187533499 | A | G | NM_001144999 | c.2306A>G:p.N769S | Missense | 0.0028 | 0.0012 | 0.0023 | 14.89 | 16 | 4 |
| ITGAV | chr2 | 187533544 | C | G | NM_001144999 | c.2351C>G:p.P784R | Missense | 0.0008 | - | 0.0008 | 31 | 2 | 0 |
| ITGAV | chr2 | 187540358 | A | G | NM_001144999 | c.2596A>G:p.I866V | Missense | - | - | - | 23.8 | 0 | 1 |
| ITGAV | chr2 | 187540382 | G | A | NM_001144999 | c.2620G>A:p.D874N | Missense | - | - | - | 26.6 | 0 | 1 |
| ITGAV | chr2 | 187540388 | G | A | NM_001144999 | c.2626G>A:p.G876R | Missense | 0 | - | - | 23.3 | 1 | 0 |
| ITGAV | chr2 | 187540581 | C | T | NM_001144999 | c.2717C>T:p.S906L | Missense | 0 | - | 0 | 32 | 0 | 1 |
| ITGAV | chr2 | 187540610 | C | T | NM_001144999 | c.2746C>T:p.P916S | Missense | 5.81E-05 | - | - | 32 | 1 | 0 |
| ITGAV | chr2 | 187541658 | A | G | NM_001144999 | c.2909A>G:p.Y970C | Missense | 5.80E-05 | - | - | 27.2 | 1 | 1 |
| ITGAV | chr2 | 187542009 | CAGAAACTTAACTG | - | NM_001144999 | c.2999_3012del:p.E1001Vfs*10 | Frameshift deletion | - | - | - | - | 1 | 0 |
| RHOA | chr3 | 49398438 | G | A | NM_001313943 | c.470C>T:p.P157L | Missense | 0 | 0 | - | 0.979 | 1 | 0 |
| RHOA | chr3 | 49399950 | C | T | NM_001313947 | c.266G>A:p.G89E | Missense | 0 | - | 0 | 17.49 | 1 | 0 |
| PIK3CB | chr3 | 138375045 | G | A | NM_006219 | c.3014C>T:p.A1005V | Missense | 0 | - | 0 | 32 | 1 | 0 |
| PIK3CB | chr3 | 138375112 | G | A | NM_006219 | c.2947C>T:p.R983C | Missense | 0 | - | 0 | 35 | 1 | 0 |
| PIK3CB | chr3 | 138376533 | G | A | NM_006219 | c.2941C>T:p.R981W;- | Missense | 0.0002 | 0.0006 | - | 34 | 1 | 2 |
| PIK3CB | chr3 | 138382857 | C | A | NM_006219 | c.2687G>T:p.R896L | Missense | 0.0035 | 0.0019 | 0.004 | 35 | 17 | 12 |
| PIK3CB | chr3 | 138384038 | G | T | NM_006219 | c.2512C>A:p.P838T | Missense | - | - | - | 26.5 | 1 | 0 |
| PIK3CB | chr3 | 138400852 | G | C | NM_006219 | c.2461C>G:p.R821G | Missense | - | - | - | 31 | 1 | 0 |
| PIK3CB | chr3 | 138403497 | A | G | NM_006219 | c.2285T>C:p.L762P | Missense | - | - | - | 28.4 | 1 | 0 |
| PIK3CB | chr3 | 138403527 | C | T | NM_006219 | c.2255G>A:p.R752Q | Missense | 5.80E-05 | 0 | 0 | 23 | 0 | 1 |
| PIK3CB | chr3 | 138403527 | C | A | NM_006219 | c.2255G>T:p.R752L | Missense | 0.0003 | - | - | 21.7 | 2 | 1 |
| PIK3CB | chr3 | 138403551 | G | T | NM_006219 | c.2231C>A:p.T744N | Missense | - | - | - | 7.455 | 1 | 0 |
| PIK3CB | chr3 | 138403632 | T | C | NM_006219 | c.2150A>G:p.N717S | Missense | 0 | 0 | 0 | 10.36 | 1 | 0 |
| PIK3CB | chr3 | 138409875 | C | T | NM_006219 | c.2003G>A:p.R668Q | Missense | 0 | 0 | - | 26.8 | 1 | 0 |
| PIK3CB | chr3 | 138413710 | G | C | NM_006219 | c.1810C>G:p.R604G | Missense | - | - | - | 17.12 | 1 | 0 |
| PIK3CB | chr3 | 138417879 | A | G | NM_006219 | c.1640T>C:p.L547S | Missense | - | - | - | 21.6 | 1 | 0 |
| PIK3CB | chr3 | 138431089 | T | A | NM_006219 | c.1360A>T:p.T454S | Missense | - | - | - | 13 | 0 | 1 |
| PIK3CB | chr3 | 138433432 | A | C | NM_006219 | c.1180T>G:p.L394V | Missense | 0.0002 | 0.0006 | 0.0002 | 21.6 | 1 | 1 |
| PIK3CB | chr3 | 138452258 | G | A | NM_006219 | c.995C>T:p.P332L | Missense | - | 0.0006 | - | 22.9 | 1 | 0 |
| PIK3CB | chr3 | 138453602 | T | C | NM_006219 | c.846A>G:p.I282M | Missense | 0 | 0 | 0 | 8.295 | 1 | 0 |
| PIK3CB | chr3 | 138453640 | G | A | NM_006219 | c.808C>T:p.R270W | Missense | 0 | 0 | 0 | 26.4 | 1 | 0 |
| PIK3CB | chr3 | 138456692 | G | A | NM_006219 | c.658C>T:p.P220S | Missense | - | - | - | 26.5 | 1 | 0 |
| PIK3CB | chr3 | 138456701 | T | C | NM_006219 | c.649A>G:p.N217D | Missense | 0.0003 | - | 0.0001 | 14.14 | 1 | 3 |
| PIK3CB | chr3 | 138461455 | T | A | NM_006219 | c.566A>T:p.E189V | Missense | - | - | - | 21 | 0 | 1 |
| PIK3CB | chr3 | 138461494 | T | C | NM_006219 | c.527A>G:p.Y176C | Missense | - | - | - | 20.5 | 1 | 0 |
| PIK3CB | chr3 | 138461563 | C | A | NM_006219 | c.458G>T:p.R153L | Missense | 0 | - | 0.0001 | 23.9 | 0 | 1 |
| PIK3CB | chr3 | 138461564 | G | A | NM_006219 | c.457C>T:p.R153C | Missense | 0.0003 | - | 0.0003 | 22.9 | 0 | 1 |
| PIK3CB | chr3 | 138474703 | C | T | NM_006219 | c.290G>A:p.R97Q | Missense | 0 | - | - | 24.3 | 2 | 0 |
| PIK3CB | chr3 | 138474779 | G | A | NM_006219 | c.214C>T:p.L72F | Missense | - | - | - | 26.1 | 0 | 1 |
| PIK3CB | chr3 | 138474799 | T | C | NM_006219 | c.194A>G:p.N65S | Missense | 0.0001 | - | 0.0001 | 1.261 | 0 | 1 |
| PIK3CB | chr3 | 138478130 | G | A | NM_006219 | c.56C>T:p.A19V | Missense | 5.80E-05 | 0 | 0 | 23.5 | 0 | 1 |
| PIK3CA | chr3 | 178917492 | A | C | NM_006218 | c.367A>C:p.M123L | Missense | - | - | - | 12.47 | 1 | 0 |
| PIK3CA | chr3 | 178917561 | G | A | NM_006218 | c.436G>A:p.V146I | Missense | 0 | - | 0 | 14.68 | 0 | 2 |
| PIK3CA | chr3 | 178917574 | C | T | NM_006218 | c.449C>T:p.A150V | Missense | - | - | - | 17.87 | 0 | 1 |
| PIK3CA | chr3 | 178917595 | A | G | NM_006218 | c.470A>G:p.N157S | Missense | 5.80E-05 | - | - | 4.343 | 1 | 1 |
| PIK3CA | chr3 | 178921464 | C | A | NM_006218 | c.946C>A:p.P316T | Missense | 0.0001 | - | - | 17.28 | 0 | 1 |
| PIK3CA | chr3 | 178927410 | A | G | NM_006218 | c.1173A>G:p.I391M | Missense | 0 | 0 | 0 | 10.19 | 1 | 2 |
| PIK3CA | chr3 | 178927481 | C | T | NM_006218 | c.1244C>T:p.A415V | Missense | - | - | - | 26 | 1 | 0 |
| PIK3CA | chr3 | 178928340 | C | T | NM_006218 | c.1526C>T:p.S509F | Missense | - | - | - | 22.9 | 1 | 0 |
| PIK3CA | chr3 | 178937462 | G | A | NM_006218 | c.1850G>A:p.R617Q | Missense | 0.0002 | - | 0.0001 | 23 | 1 | 1 |
| PIK3CA | chr3 | 178937492 | A | G | NM_006218 | c.1880A>G:p.K627R | Missense | 5.82E-05 | - | - | 23.9 | 1 | 0 |
| PIK3CA | chr3 | 178941879 | A | G | NM_006218 | c.2198A>G:p.K733R | Missense | 0.0058 | 0.0062 | 0.0059 | 22.4 | 12 | 8 |
| PIK3CA | chr3 | 178941888 | T | C | NM_006218 | c.2207T>C:p.V736A | Missense | 0 | - | 0 | 18.71 | 1 | 0 |
| PIK3CA | chr3 | 178942555 | A | G | NM_006218 | c.2362A>G:p.I788V | Missense | 5.80E-05 | - | - | 10.42 | 1 | 1 |
| LIMK1 | chr7 | 73498334 | T | C | NM_002314 | c.14T>C:p.L5P | Missense | - | - | - | 21.3 | 0 | 1 |
| LIMK1 | chr7 | 73500083 | G | A | NM_002314 | c.61G>A:p.E21K | Missense | 0.0002 | 0 | 0 | 16.89 | 2 | 2 |
| LIMK1 | chr7 | 73500113 | A | G | NM_002314 | c.91A>G:p.R31G | Missense | - | - | - | 17.31 | 0 | 1 |
| LIMK1 | chr7 | 73507610 | C | T | NM_001204426 | c.31C>T:p.R11C | Missense | 0 | 0.0006 | - | 7.703 | 2 | 0 |
| LIMK1 | chr7 | 73507610 | C | G | NM_001204426 | c.31C>G:p.R11G | Missense | 0 | 0 | - | 5.542 | 0 | 1 |
| LIMK1 | chr7 | 73511044 | A | G | NM_002314 | c.245A>G:p.E82G | Missense | - | - | - | 23 | 1 | 0 |
| LIMK1 | chr7 | 73511492 | C | T | NM_002314 | c.374C>T:p.T125M | Missense | 0 | 0 | 0 | 25.6 | 1 | 0 |
| LIMK1 | chr7 | 73513414 | C | A | NM_002314 | c.454C>A:p.P152T | Missense | - | - | - | 22.7 | 1 | 0 |
| LIMK1 | chr7 | 73513421 | C | T | NM_002314 | c.461C>T:p.S154F | Missense | - | - | - | 32 | 1 | 0 |
| LIMK1 | chr7 | 73513514 | C | T | NM_002314 | c.554C>T:p.P185L | Missense | 0 | 0 | 0 | 19.03 | 1 | 1 |
| LIMK1 | chr7 | 73513519 | G | A | NM_002314 | c.559G>A:p.G187S | Missense | 0 | - | 0.0001 | 10.7 | 1 | 0 |
| LIMK1 | chr7 | 73513555 | G | A | NM_002314 | c.595G>A:p.V199I | Missense | 0 | 0 | 0 | 29.2 | 0 | 1 |
| LIMK1 | chr7 | 73520435 | G | A | NM_002314 | c.743G>A:p.R248H | Missense | 0.001 | 0 | 0.0012 | 23.7 | 7 | 11 |
| LIMK1 | chr7 | 73520543 | C | T | NM_002314 | c.851C>T:p.A284V | Missense | 0.001 | 0.0012 | 0.0009 | 8.774 | 1 | 0 |
| LIMK1 | chr7 | 73520557 | C | T | NM_002314 | c.865C>T:p.R289W | Missense | 0.0001 | - | 0.0001 | 32 | 5 | 0 |
| LIMK1 | chr7 | 73520558 | G | A | NM_002314 | c.866G>A:p.R289Q | Missense | 0 | 0.0006 | 0 | 22.4 | 1 | 1 |
| LIMK1 | chr7 | 73521381 | T | G | NM_002314 | c.923T>G:p.L308R | Missense | - | - | - | 9.127 | 1 | 0 |
| LIMK1 | chr7 | 73521402 | G | A | NM_002314 | c.944G>A:p.R315H | Missense | 0.0015 | 0.0006 | 0.0006 | 19.88 | 5 | 4 |
| LIMK1 | chr7 | 73521468 | C | T | NM_002314 | c.1010C>T:p.S337L | Missense | 0.0003 | 0.0006 | 0 | 26.6 | 0 | 2 |
| LIMK1 | chr7 | 73521499 | G | C | NM_002314 | c.1041G>C:p.K347N | Missense | - | - | - | 25.4 | 1 | 0 |
| LIMK1 | chr7 | 73523268 | G | T | NM_002314 | c.1186G>T:p.V396L | Missense | - | - | - | 32 | 1 | 0 |
| LIMK1 | chr7 | 73523341 | C | T | NM_002314 | c.1259C>T:p.T420M | Missense | 0 | - | 0 | 34 | 0 | 1 |
| LIMK1 | chr7 | 73523350 | G | A | NM_002314 | c.1268G>A:p.G423D | Missense | - | - | - | 8.966 | 0 | 1 |
| LIMK1 | chr7 | 73530286 | A | G | NM_002314 | c.1565A>G:p.N522S | Missense | 5.80E-05 | - | 0.0001 | 23.3 | 3 | 0 |
| LIMK1 | chr7 | 73535220 | A | T | NM_002314 | c.1624-2A>T:c.1522-2AT | Splicing | - | - | - | 23.9 | 0 | 1 |
| LIMK1 | chr7 | 73535315 | C | G | NM_001204426 | c.1615C>G:p.P539A | Missense | 0 | - | 0 | 0.013 | 0 | 1 |
| LIMK1 | chr7 | 73535352 | G | A | NM_001204426 | c.1652G>A:p.R551H | Missense | 0 | - | 0 | 21.1 | 1 | 0 |
| LIMK1 | chr7 | 73535524 | G | A | NM_001204426 | c.1735G>A:p.G579S | Missense | 5.83E-05 | - | 0 | 12.79 | 1 | 0 |
| LIMK1 | chr7 | 73535530 | C | A | NM_001204426 | c.1741C>A:p.L581M | Missense | 0.0002 | - | 0.0003 | 19.84 | 0 | 1 |
| LIMK1 | chr7 | 73535585 | G | A | NM_001204426 | c.1796G>A:p.R599Q | Missense | 0 | - | 0 | 22.6 | 1 | 0 |
| LIMK1 | chr7 | 73535587 | C | G | NM_001204426 | c.1798C>G:p.R600G | Missense | - | - | - | 23 | 0 | 1 |
| LIMK1 | chr7 | 73535596 | A | G | NM_001204426 | c.1807A>G:p.S603G | Missense | - | - | - | 11.93 | 1 | 0 |
| DPYSL2 | chr8 | 26371846 | C | A | NM_001197293 | c.5C>A:p.A2D | Missense | 0.0001 | - | - | - | 1 | 1 |
| DPYSL2 | chr8 | 26372002 | C | G | NM_001197293 | c.161C>G:p.S54W | Missense | 0 | - | 0 | - | 0 | 1 |
| DPYSL2 | chr8 | 26372058 | C | T | NM_001197293 | c.217C>T:p.H73Y | Missense | 0.0007 | - | - | - | 2 | 4 |
| DPYSL2 | chr8 | 26439569 | A | T | NM_001197293 | c.439A>T:p.I147F | Missense | - | - | - | 28 | 0 | 1 |
| DPYSL2 | chr8 | 26484172 | G | A | NM_001197293 | c.833G>A:p.R278H | Missense | 0.0038 | 0.0012 | 0.0031 | 22.8 | 19 | 14 |
| DPYSL2 | chr8 | 26484184 | C | T | NM_001197293 | c.845C>T:p.T282M | Missense | 0 | 0 | 0 | 34 | 0 | 1 |
| DPYSL2 | chr8 | 26484761 | A | G | NM_001197293 | c.886A>G:p.I296V | Missense | - | - | - | 22.7 | 1 | 0 |
| DPYSL2 | chr8 | 26485419 | C | T | NM_001197293 | c.968C>T:p.T323M | Missense | 0.0001 | - | 0.0001 | 34 | 0 | 2 |
| DPYSL2 | chr8 | 26501524 | G | A | NM_001197293 | c.1342G>A:p.V448I | Missense | 0.0002 | 0 | 0.0001 | 19.52 | 1 | 1 |
| DPYSL2 | chr8 | 26501564 | A | G | NM_001197293 | c.1382A>G:p.N461S | Missense | 0 | - | 0 | 26.2 | 1 | 1 |
| DPYSL2 | chr8 | 26505233 | C | T | NM_001197293 | c.1513C>T:p.R505C | Missense | 0 | 0 | 0 | 34 | 0 | 1 |
| DPYSL2 | chr8 | 26509809 | C | T | NM_001197293 | c.1633C>T:p.R545C | Missense | 0.0001 | - | 0.0001 | 35 | 0 | 2 |
| DPYSL2 | chr8 | 26509878 | G | A | NM_001197293 | c.1702G>A:p.E568K | Missense | 0 | 0 | - | 23.4 | 1 | 0 |
| DPYSL2 | chr8 | 26509893 | T | C | NM_001197293 | c.1717T>C:p.Y573H | Missense | - | - | - | 29 | 1 | 0 |
| DPYSL2 | chr8 | 26513182 | C | T | NM_001197293 | c.1994C>T:p.A665V | Missense | 0 | - | - | 24.4 | 1 | 0 |
| DPYSL2 | chr8 | 26513197 | G | A | NM_001197293 | c.2009G>A:p.R670H | Missense | 5.82E-05 | 0 | - | 34 | 0 | 1 |
| NTRK2 | chr9 | 87285694 | G | A | NM_001369538 | c.31G>A:p.A11T | Missense | 0.0002 | - | 0.0001 | 22.7 | 1 | 2 |
| NTRK2 | chr9 | 87285730 | G | A | NM_001369538 | c.67G>A:p.V23M | Missense | 0 | - | 0 | 9.037 | 1 | 0 |
| NTRK2 | chr9 | 87285820 | G | A | NM_001369538 | c.157G>A:p.V53M | Missense | 0.0002 | - | 0.0003 | 19.52 | 1 | 0 |
| NTRK2 | chr9 | 87285865 | A | G | NM_001369538 | c.202A>G:p.I68V | Missense | 0 | - | 0 | 13.55 | 1 | 0 |
| NTRK2 | chr9 | 87285872 | A | C | NM_001369538 | c.209A>C:p.E70A | Missense | - | - | - | 26.7 | 0 | 1 |
| NTRK2 | chr9 | 87322782 | C | T | NM_001369538 | c.383C>T:p.T128M | Missense | 5.80E-05 | - | 0.0001 | 25.5 | 1 | 1 |
| NTRK2 | chr9 | 87322806 | G | A | NM_001369538 | c.407G>A:p.R136H | Missense | 0.0002 | - | 0.0001 | 22.8 | 1 | 0 |
| NTRK2 | chr9 | 87338488 | G | T | NM_001369538 | c.584G>T:p.G195V | Missense | 0.0001 | - | 0.0001 | 19.37 | 0 | 1 |
| NTRK2 | chr9 | 87339190 | G | A | NM_001369538 | c.772G>A:p.D258N | Missense | - | - | - | 22.7 | 1 | 0 |
| NTRK2 | chr9 | 87342675 | C | G | NM_001369538 | c.960C>G:p.N320K | Missense | 5.80E-05 | - | - | 6.404 | 0 | 1 |
| NTRK2 | chr9 | 87342827 | A | G | NM_001369538 | c.1112A>G:p.E371G | Missense | - | - | - | 24.5 | 1 | 0 |
| NTRK2 | chr9 | 87342863 | G | T | NM_001369538 | c.1148G>T:p.G383V | Missense | - | - | - | 13.77 | 1 | 0 |
| NTRK2 | chr9 | 87563390 | C | T | NM_001369534 | c.1694C>T:p.A565V | Missense | - | - | - | 24.6 | 1 | 0 |
| NTRK2 | chr9 | 87563428 | G | A | NM_001369534 | c.1732G>A:p.E578K | Missense | - | - | - | 35 | 0 | 1 |
| NTRK2 | chr9 | 87570260 | C | T | NM_001369534 | c.1916C>T:p.S639L | Missense | 0 | - | 0 | 32 | 1 | 0 |
| AKT1 | chr14 | 105236757 | T | C | NM_001014431 | c.1364A>G:p.D455G | Missense | - | - | 0.0001 | 18.36 | 1 | 0 |
| AKT1 | chr14 | 105239256 | C | A | NM_001014431 | c.1131G>T:p.K377N | Missense | - | - | - | 24.3 | 0 | 1 |
| AKT1 | chr14 | 105239892 | C | T | NM_001014431 | c.728G>A:p.R243H | Missense | - | - | - | 34 | 1 | 0 |
| AKT1 | chr14 | 105240317 | C | T | NM_001014431 | c.634G>A:p.A212T | Missense | - | - | - | 20.5 | 0 | 1 |
| AKT1 | chr14 | 105241460 | G | A | NM_001014431 | c.520C>T:p.R174C | Missense | 5.80E-05 | 0 | - | 26.7 | 0 | 1 |
| AKT1 | chr14 | 105242022 | C | T | NM_001014431 | c.402G>A:p.M134I | Missense | - | - | - | 22.9 | 0 | 1 |
| AKT1 | chr14 | 105246479 | G | A | NM_001014431 | c.121C>T:p.R41W | Missense | 0 | - | 0 | 24.7 | 1 | 0 |
| LINGO1 | chr15 | 77906424 | C | T | NM_032808 | c.1825G>A:p.A609T | Missense | 0 | - | 0 | 5.139 | 1 | 1 |
| LINGO1 | chr15 | 77906508 | G | A | NM_032808 | c.1741C>T:p.L581F | Missense | - | - | - | 23 | 0 | 1 |
| LINGO1 | chr15 | 77906637 | C | T | NM_032808 | c.1612G>A:p.E538K | Missense | - | - | - | 23.3 | 0 | 1 |
| LINGO1 | chr15 | 77906828 | A | G | NM_032808 | c.1421T>C:p.L474P | Missense | 0 | - | - | 25.6 | 0 | 1 |
| LINGO1 | chr15 | 77906864 | C | T | NM_032808 | c.1385G>A:p.R462Q | Missense | 0 | 0 | 0 | 14.6 | 0 | 1 |
| LINGO1 | chr15 | 77906909 | C | T | NM_032808 | c.1340G>A:p.R447Q | Missense | 0.0003 | - | 0 | 15.18 | 2 | 1 |
| LINGO1 | chr15 | 77906984 | C | T | NM_032808 | c.1265G>A:p.R422H | Missense | 5.90E-05 | 0.0006 | - | 22.9 | 0 | 1 |
| LINGO1 | chr15 | 77907101 | C | T | NM_032808 | c.1148G>A:p.R383H | Missense | 0 | - | - | 32 | 1 | 0 |
| LINGO1 | chr15 | 77907201 | G | C | NM_032808 | c.1048C>G:p.L350V | Missense | 0 | - | 0 | 22.8 | 1 | 0 |
| LINGO1 | chr15 | 77907254 | C | T | NM_032808 | c.995G>A:p.R332H | Missense | 0 | 0.0006 | 0.0001 | 24.6 | 0 | 2 |
| LINGO1 | chr15 | 77907288 | C | T | NM_032808 | c.961G>A:p.G321R | Missense | 0 | 0 | 0 | 27.7 | 0 | 1 |
| LINGO1 | chr15 | 77907428 | T | A | NM_032808 | c.821A>T:p.N274I | Missense | - | - | 0.0001 | 25.8 | 1 | 0 |
| LINGO1 | chr15 | 77907582 | G | A | NM_032808 | c.667C>T:p.R223W | Missense | 0 | - | 0 | 32 | 0 | 1 |
| LINGO1 | chr15 | 77907884 | C | T | NM_032808 | c.365G>A:p.R122Q | Missense | 0 | 0 | 0 | 22.7 | 1 | 0 |
| LINGO1 | chr15 | 77907979 | C | G | NM_032808 | c.270G>C:p.E90D | Missense | - | - | - | 0.778 | 1 | 0 |
| LINGO1 | chr15 | 77908215 | C | T | NM_032808 | c.34G>A:p.V12M | Missense | 0 | 0.0006 | 0.0016 | 14.78 | 1 | 3 |
| NGFR | chr17 | 47572784 | G | C | NM_002507 | c.5G>C:p.G2A | Missense | - | - | - | 8.299 | 1 | 0 |
| NGFR | chr17 | 47579423 | A | G | NM_002507 | c.67-2A>G | Splicing | - | - | - | 23.8 | 0 | 1 |
| NGFR | chr17 | 47579431 | C | T | NM_002507 | c.73C>T:p.L25F | Missense | 0.0094 | 0.0081 | 0.0078 | 6.467 | 59 | 35 |
| NGFR | chr17 | 47579454 | C | A | NM_002507 | c.96C>A:p.C32X | Stopgain | - | - | - | 29.9 | 1 | 0 |
| NGFR | chr17 | 47579479 | G | A | NM_002507 | c.121G>A:p.G41S | Missense | 0.0004 | - | 0.0003 | 28.4 | 1 | 0 |
| NGFR | chr17 | 47579503 | C | G | NM_002507 | c.145C>G:p.L49V | Missense | - | - | - | 8.018 | 0 | 1 |
| NGFR | chr17 | 47583678 | G | A | NM_002507 | c.226G>A:p.V76M | Missense | 5.94E-05 | - | 0.0001 | 28 | 0 | 1 |
| NGFR | chr17 | 47583814 | G | A | NM_002507 | c.362G>A:p.R121H | Missense | - | - | - | 15.57 | 1 | 0 |
| NGFR | chr17 | 47583823 | C | T | NM_002507 | c.371C>T:p.A124V | Missense | - | - | - | 22.9 | 1 | 0 |
| NGFR | chr17 | 47583869 | G | - | NM_002507 | c.417delG:p.D140Tfs*84 | Frameshift deletion | - | - | - | - | 0 | 1 |
| NGFR | chr17 | 47583930 | C | A | NM_002507 | c.478C>A:p.H160N | Missense | - | - | - | 20.6 | 1 | 0 |
| NGFR | chr17 | 47584005 | G | A | NM_002507 | c.553G>A:p.D185N | Missense | 0.0012 | 0.0019 | 0.0003 | 24.1 | 5 | 1 |
| NGFR | chr17 | 47584011 | G | A | NM_002507 | c.559G>A:p.E187K | Missense | 0 | - | - | 23.4 | 0 | 1 |
| NGFR | chr17 | 47587797 | C | T | NM_002507 | c.592C>T:p.R198W | Missense | 0 | 0 | 0 | 20.9 | 1 | 0 |
| NGFR | chr17 | 47587831 | C | T | NM_002507 | c.626C>T:p.A209V | Missense | - | - | - | 1.284 | 1 | 0 |
| NGFR | chr17 | 47587917 | A | G | NM_002507 | c.712A>G:p.S238G | Missense | 0.0005 | 0.0006 | 0.0007 | 20.1 | 7 | 2 |
| NGFR | chr17 | 47587921 | CCCAGCCCGTGGTGA | - | NM_002507 | c.716_730del:p.Q240_T244del | NonFrameshift deletion | - | - | - | - | 1 | 0 |
| NGFR | chr17 | 47587929 | G | A | NM_002507 | c.724G>A:p.V242M | Missense | 0 | 0 | - | 24.4 | 0 | 1 |
| NGFR | chr17 | 47587929 | G | - | NM_002507 | c.724delG:p.V242Wfs*1 | Frameshift deletion | - | - | - | - | 1 | 0 |
| NGFR | chr17 | 47587996 | T | C | NM_002507 | c.791T>C:p.V264A | Missense | - | - | - | 20.5 | 2 | 1 |
| NGFR | chr17 | 47588013 | A | G | NM_002507 | c.808A>G:p.I270V | Missense | 0.0004 | 0 | 0.0003 | 14.15 | 2 | 4 |
| NGFR | chr17 | 47588014 | T | A | NM_002507 | c.809T>A:p.I270K | Missense | - | - | - | 33 | 0 | 1 |
| NGFR | chr17 | 47590083 | C | A | NM_002507 | c.996C>A:p.D332E | Missense | - | - | - | 13.89 | 0 | 1 |
| NGFR | chr17 | 47590156 | G | C | NM_002507 | c.1069G>C:p.D357H | Missense | - | - | - | 18.42 | 1 | 0 |
| NGFR | chr17 | 47590198 | G | A | NM_002507 | c.1111G>A:p.E371K | Missense | 0 | - | - | 34 | 1 | 0 |
| NGFR | chr17 | 47590238 | G | A | NM_002507 | c.1151G>A:p.R384H | Missense | 0 | - | 0 | 18.7 | 1 | 0 |
| NGFR | chr17 | 47590283 | A | G | NM_002507 | c.1196A>G:p.D399G | Missense | 5.87E-05 | - | - | 25 | 1 | 0 |
| NGFR | chr17 | 47590297 | G | A | NM_002507 | c.1210G>A:p.A404T | Missense | - | 0 | - | 34 | 1 | 0 |
| NGFR | chr17 | 47590303 | C | T | NM_002507 | c.1216C>T:p.R406C | Missense | 0.0001 | - | 0.0001 | 35 | 1 | 2 |
| NGFR | chr17 | 47590304 | G | A | NM_002507 | c.1217G>A:p.R406H | Missense | 0 | - | 0 | 23.5 | 1 | 0 |
| NGFR | chr17 | 47590307 | G | A | NM_002507 | c.1220G>A:p.R407H | Missense | 0.0011 | 0.0025 | 0.0009 | 34 | 6 | 4 |
| NGFR | chr17 | 47590327 | G | A | NM_002507 | c.1240G>A:p.V414M | Missense | 0 | - | 0 | 26.7 | 0 | 2 |
| NGFR | chr17 | 47590364 | C | T | NM_002507 | c.1277C>T:p.P426L | Missense | 0.0001 | 0 | 0 | 26.2 | 0 | 1 |
| ROCK1 | chr18 | 18533560 | T | C | NM_005406 | c.4040A>G:p.K1347R | Missense | 6.44E-05 | - | - | 23.5 | 2 | 0 |
| ROCK1 | chr18 | 18533600 | T | C | NM_005406 | c.4000A>G:p.T1334A | Missense | 0 | 0 | 0 | 15.96 | 0 | 1 |
| ROCK1 | chr18 | 18533608 | G | A | NM_005406 | c.3992C>T:p.T1331M | Missense | 0.0012 | 0.0006 | 0.0007 | 24.7 | 9 | 7 |
| ROCK1 | chr18 | 18533623 | C | T | NM_005406 | c.3977G>A:p.R1326H | Missense | 0.0002 | - | 0.0005 | 34 | 1 | 0 |
| ROCK1 | chr18 | 18533644 | T | A | NM_005406 | c.3956A>T:p.N1319I | Missense | 0 | - | 0 | 22.8 | 0 | 1 |
| ROCK1 | chr18 | 18534989 | T | G | NM_005406 | c.3608A>C:p.E1203A | Missense | - | - | - | 29 | 1 | 0 |
| ROCK1 | chr18 | 18546944 | C | T | NM_005406 | c.3286G>A:p.D1096N | Missense | - | - | - | 22.9 | 1 | 0 |
| ROCK1 | chr18 | 18547849 | C | T | NM_005406 | c.3056G>A:p.R1019K | Missense | - | - | - | 16.55 | 1 | 0 |
| ROCK1 | chr18 | 18547852 | T | C | NM_005406 | c.3053A>G:p.D1018G | Missense | - | - | - | 19.9 | 1 | 0 |
| ROCK1 | chr18 | 18548791 | C | T | NM_005406 | c.2945G>A:p.S982N | Missense | - | - | - | 13.73 | 0 | 1 |
| ROCK1 | chr18 | 18550379 | G | A | NM_005406 | c.2750C>T:p.T917M | Missense | 0 | 0 | 0 | 23.6 | 1 | 0 |
| ROCK1 | chr18 | 18564483 | G | C | NM_005406 | c.2318C>G:p.T773S | Missense | 5.90E-05 | 0.0006 | 0 | 12.92 | 0 | 1 |
| ROCK1 | chr18 | 18571149 | C | T | NM_005406 | c.2131G>A:p.V711M | Missense | - | 0.0006 | - | 32 | 2 | 2 |
| ROCK1 | chr18 | 18571191 | G | A | NM_005406 | c.2089C>T:p.R697C | Missense | 5.80E-05 | - | - | 34 | 0 | 1 |
| ROCK1 | chr18 | 18571233 | G | A | NM_005406 | c.2047C>T:p.R683W | Missense | 5.84E-05 | 0 | 0 | 27.5 | 0 | 1 |
| ROCK1 | chr18 | 18586339 | C | T | NM_005406 | c.1858G>A:p.D620N | Missense | - | - | - | 22.9 | 1 | 0 |
| ROCK1 | chr18 | 18586481 | G | C | NM_005406 | c.1716C>G:p.S572R | Missense | - | - | - | 15.13 | 0 | 1 |
| ROCK1 | chr18 | 18586491 | G | A | NM_005406 | c.1706C>T:p.T569I | Missense | - | - | - | 25.5 | 0 | 1 |
| ROCK1 | chr18 | 18586551 | T | G | NM_005406 | c.1646A>C:p.E549A | Missense | - | - | - | 24.4 | 0 | 1 |
| ROCK1 | chr18 | 18588155 | C | T | NM_005406 | c.1411G>A:p.G471R;- | Missense | - | - | - | 22.8 | 1 | 0 |
| ROCK1 | chr18 | 18603634 | A | G | NM_005406 | c.1219T>C:p.S407P | Missense | 6.34E-05 | - | 0.0001 | 23 | 1 | 0 |
| ROCK1 | chr18 | 18619499 | C | T | NM_005406 | c.985G>A:p.V329I | Missense | 5.80E-05 | - | 0.0001 | 22.5 | 0 | 1 |
| ROCK1 | chr18 | 18619521 | T | G | NM_005406 | c.963A>C:p.E321D | Missense | - | - | - | 13.84 | 0 | 1 |
| ROCK1 | chr18 | 18622059 | T | C | NM_005406 | c.958A>G:p.R320G;- | Missense | - | - | - | 28 | 0 | 1 |
| ROCK1 | chr18 | 18622088 | T | C | NM_005406 | c.929A>G:p.K310R | Missense | 0.0002 | - | 0.0003 | 21.6 | 11 | 4 |
| ROCK1 | chr18 | 18625299 | C | A | NM_005406 | c.544G>T:p.V182L | Missense | - | - | - | 25.2 | 0 | 1 |
| ROCK1 | chr18 | 18625358 | A | C | NM_005406 | c.485T>G:p.V162G | Missense | - | - | - | 27.8 | 1 | 0 |
| ROCK1 | chr18 | 18629168 | T | C | NM_005406 | c.299A>G:p.K100R | Missense | - | - | - | 17.21 | 1 | 0 |
| ROCK1 | chr18 | 18690839 | A | T | NM_005406 | c.33T>A:p.F11L | Missense | - | - | - | 11.84 | 0 | 1 |
| S1PR2 | chr19 | 10334570 | T | C | NM_004230 | c.1012A>G:p.M338V | Missense | - | - | - | 2.227 | 0 | 1 |
| S1PR2 | chr19 | 10334639 | G | A | NM_004230 | c.943C>T:p.R315W | Missense | 0.0013 | 0.0006 | 0.0012 | 24.2 | 1 | 2 |
| S1PR2 | chr19 | 10334663 | T | A | NM_004230 | c.919A>T:p.R307W | Missense | 0.0042 | 0.0075 | 0.0033 | 19.87 | 24 | 10 |
| S1PR2 | chr19 | 10334677 | G | A | NM_004230 | c.905C>T:p.P302L | Missense | 0 | - | 0 | 12.45 | 1 | 0 |
| S1PR2 | chr19 | 10334692 | C | T | NM_004230 | c.890G>A:p.R297Q | Missense | 0 | - | - | 22.9 | 0 | 1 |
| S1PR2 | chr19 | 10334725 | A | G | NM_004230 | c.857T>C:p.V286A | Missense | 0.0021 | 0.0019 | 0.0025 | 26 | 4 | 4 |
| S1PR2 | chr19 | 10334731 | T | - | NM_004230 | c.851delA:p.N284Tfs*110 | Frameshift deletion | - | - | - | - | 1 | 1 |
| S1PR2 | chr19 | 10334735 | G | - | NM_004230 | c.847delC:p.L283Sfs*111 | Frameshift deletion | - | - | - | - | 1 | 1 |
| S1PR2 | chr19 | 10334801 | C | T | NM_004230 | c.781G>A:p.V261I | Missense | 0 | 0 | 0 | 4.772 | 1 | 0 |
| S1PR2 | chr19 | 10334855 | T | C | NM_004230 | c.727A>G:p.I243V | Missense | - | - | - | 14.59 | 0 | 1 |
| S1PR2 | chr19 | 10334929 | G | A | NM_004230 | c.653C>T:p.S218L | Missense | 0 | - | - | 28 | 1 | 0 |
| S1PR2 | chr19 | 10334933 | G | A | NM_004230 | c.649C>T:p.R217C | Missense | 0 | - | 0 | 34 | 0 | 1 |
| S1PR2 | chr19 | 10334954 | C | A | NM_004230 | c.628G>T:p.V210L | Missense | - | - | - | 9.702 | 0 | 1 |
| S1PR2 | chr19 | 10334966 | C | G | NM_004230 | c.616G>C:p.V206L | Missense | - | - | - | 24.7 | 1 | 0 |
| S1PR2 | chr19 | 10335280 | G | A | NM_004230 | c.302C>T:p.T101M | Missense | 0.0002 | - | 0.0003 | 24.4 | 2 | 0 |
| S1PR2 | chr19 | 10335403 | C | T | NM_004230 | c.179G>A:p.R60Q | Missense | 0.0028 | 0.0037 | 0.0027 | 27.5 | 11 | 14 |
| S1PR2 | chr19 | 10335511 | G | A | NM_004230 | c.71C>T:p.T24M | Missense | 0 | 0 | 0 | 11.62 | 1 | 0 |
| S1PR2 | chr19 | 10335552 | G | T | NM_004230 | c.30C>A:p.N10K | Missense | 0.0006 | 0 | 0.0006 | 14.19 | 0 | 4 |
| S1PR2 | chr19 | 10335566 | A | G | NM_004230 | c.16T>C:p.S6P | Missense | - | - | - | 24.6 | 0 | 3 |
| LILRB1 | chr19 | 55142727 | A | T | NM_001081637 | c.40A>T:p.S14C | Missense | - | - | - | 12.83 | 1 | 0 |
| LILRB1 | chr19 | 55142739 | A | T | NM_001081637 | c.52A>T:p.R18W | Missense | 5.80E-05 | - | 0 | - | 2 | 0 |
| LILRB1 | chr19 | 55142742 | A | G | NM_001081637 | c.55A>G:p.T19A | Missense | - | - | - | 16.63 | 1 | 0 |
| LILRB1 | chr19 | 55142748 | G | A | NM_001081637 | c.61G>A:p.V21M | Missense | 0.0002 | - | 0.0002 | - | 1 | 2 |
| LILRB1 | chr19 | 55142951 | G | T | NM_001081637 | c.71G>T:p.G24V | Missense | 0.0002 | - | - | 18.55 | 1 | 0 |
| LILRB1 | chr19 | 55143034 | G | A | NM_001081637 | c.154G>A:p.G52S | Missense | 0 | 0 | 0 | 0.002 | 0 | 1 |
| LILRB1 | chr19 | 55143061 | T | C | NM_001081637 | c.181T>C:p.Y61H | Missense | - | - | - | 0.002 | 0 | 1 |
| LILRB1 | chr19 | 55143089 | T | C | NM_001081637 | c.209T>C:p.I70T | Missense | - | - | - | 0.001 | 0 | 1 |
| LILRB1 | chr19 | 55143158 | C | G | NM_001081637 | c.278C>G:p.A93G | Missense | - | - | - | 12.53 | 1 | 0 |
| LILRB1 | chr19 | 55143199 | C | T | NM_001081637 | c.319C>T:p.R107C | Missense | 0.0001 | 0 | 0.0002 | 22.7 | 0 | 1 |
| LILRB1 | chr19 | 55143217 | C | T | NM_001081637 | c.337C>T:p.P113S | Missense | - | - | - | 7.975 | 1 | 0 |
| LILRB1 | chr19 | 55143519 | C | A | NM_001081637 | c.492C>A:p.H164Q | Missense | 5.80E-05 | - | - | 0.734 | 0 | 2 |
| LILRB1 | chr19 | 55143523 | C | G | NM_001081637 | c.496C>G:p.Q166E | Missense | 0.0003 | 0.0006 | 0.0003 | 0.003 | 1 | 1 |
| LILRB1 | chr19 | 55143550 | C | T | NM_001081637 | c.523C>T:p.R175C | Missense | 0.0002 | 0.0006 | 0.0002 | 11.99 | 1 | 1 |
| LILRB1 | chr19 | 55143551 | G | A | NM_001081637 | c.524G>A:p.R175H | Missense | 0.0002 | 0.0012 | 0.0002 | 0.007 | 3 | 2 |
| LILRB1 | chr19 | 55143565 | G | A | NM_001081637 | c.538G>A:p.A180T | Missense | 0.0002 | - | 0.0001 | 23 | 0 | 1 |
| LILRB1 | chr19 | 55143599 | G | A | NM_001081637 | c.572G>A:p.R191H | Missense | 0.0001 | - | 0.0002 | 0.003 | 1 | 0 |
| LILRB1 | chr19 | 55143607 | T | A | NM_001081637 | c.580T>A:p.W194R | Missense | - | - | - | 0.134 | 0 | 1 |
| LILRB1 | chr19 | 55143609 | G | A | NM_001081637 | c.582G>A:p.W194X | Stopgain | 0.0008 | 0.0006 | 0.0013 | 23.8 | 1 | 2 |
| LILRB1 | chr19 | 55143628 | G | A | NM_001081637 | c.601G>A:p.D201N | Missense | - | - | - | 0.187 | 1 | 1 |
| LILRB1 | chr19 | 55143632 | C | T | NM_001081637 | c.605C>T:p.S202L | Missense | 0.0048 | 0.0043 | 0.005 | 2.983 | 24 | 16 |
| LILRB1 | chr19 | 55143647 | A | T | NM_001081637 | c.620A>T:p.E207V | Missense | 0 | 0 | 0 | 0.001 | 1 | 0 |
| LILRB1 | chr19 | 55143650 | G | A | NM_001081637 | c.623G>A:p.W208X | Stopgain | - | - | - | 27.2 | 0 | 1 |
| LILRB1 | chr19 | 55143689 | G | A | NM_001081637 | c.661+1G>A | Splicing | - | - | - | 13.28 | 0 | 1 |
| LILRB1 | chr19 | 55143975 | C | A | NM_001081637 | c.722C>A:p.T241N | Missense | - | - | - | 0.001 | 0 | 1 |
| LILRB1 | chr19 | 55144033 | C | G | NM_001081637 | c.780C>G:p.D260E | Missense | 0 | 0 | 0 | 0.001 | 1 | 0 |
| LILRB1 | chr19 | 55144064 | G | A | NM_001081637 | c.811G>A:p.A271T | Missense | 0 | 0 | 0 | 0.05 | 3 | 0 |
| LILRB1 | chr19 | 55144100 | A | G | NM_001081637 | c.847A>G:p.T283A | Missense | 0.0003 | 0 | 0.0001 | 0.001 | 0 | 4 |
| LILRB1 | chr19 | 55144106 | G | A | NM_001081637 | c.853G>A:p.G285S | Missense | 0 | - | 0 | 0.152 | 1 | 0 |
| LILRB1 | chr19 | 55144125 | A | G | NM_001081637 | c.872A>G:p.Y291C | Missense | 0.0003 | 0.0012 | 0.0005 | 6.152 | 2 | 4 |
| LILRB1 | chr19 | 55144131 | G | T | NM_001081637 | c.878G>T:p.G293V | Missense | 0 | 0 | 0 | 22.6 | 0 | 1 |
| LILRB1 | chr19 | 55144146 | A | C | NM_001081637 | c.893A>C:p.Y298S | Missense | 5.80E-05 | 0 | 0.0001 | 0.103 | 1 | 1 |
| LILRB1 | chr19 | 55144174 | G | C | NM_001081637 | c.921G>C:p.W307C | Missense | 5.80E-05 | - | 0.0001 | 16.36 | 1 | 1 |
| LILRB1 | chr19 | 55144175 | T | C | NM_001081637 | c.922T>C:p.S308P | Missense | - | - | - | 22.5 | 0 | 1 |
| LILRB1 | chr19 | 55144179 | C | T | NM_001081637 | c.926C>T:p.A309V | Missense | 0.0001 | 0.0006 | 0.0001 | 9.193 | 2 | 0 |
| LILRB1 | chr19 | 55144189 | C | A | NM_001081637 | c.936C>A:p.D312E | Missense | - | - | - | 1.319 | 1 | 0 |
| LILRB1 | chr19 | 55144208 | G | A | NM_001081637 | c.955G>A:p.A319T | Missense | 0.0015 | 0.0006 | 0.007 | 0.001 | 21 | 1 |
| LILRB1 | chr19 | 55144481 | AG | - | NM_001081637 | c.973_974del:p.R325Sfs*38 | Frameshift deletion | 0 | - | - | - | 1 | 0 |
| LILRB1 | chr19 | 55144494 | C | T | NM_001081637 | c.986C>T:p.S329L | Missense | 0.0001 | - | 0.0001 | 8.896 | 0 | 1 |
| LILRB1 | chr19 | 55144501 | G | T | NM_001081637 | c.993G>T:p.Q331H | Missense | 0.0026 | 0.0037 | 0.0022 | 0.001 | 6 | 1 |
| LILRB1 | chr19 | 55144532 | G | A | NM_001081637 | c.1024G>A:p.V342M | Missense | 5.80E-05 | - | 0.0001 | 23.3 | 0 | 3 |
| LILRB1 | chr19 | 55144555 | G | C | NM_001081637 | c.1047G>C:p.Q349H | Missense | 0 | - | - | 0.003 | 1 | 0 |
| LILRB1 | chr19 | 55144611 | G | A | NM_001081637 | c.1103G>A:p.R368H | Missense | 0.0003 | 0 | 0.0006 | 0.064 | 0 | 1 |
| LILRB1 | chr19 | 55144623 | C | T | NM_001081637 | c.1115C>T:p.T372M | Missense | 0.0003 | 0 | 0.0001 | 0.004 | 0 | 4 |
| LILRB1 | chr19 | 55144628 | C | T | NM_001081637 | c.1120C>T:p.Q374X | Stopgain | - | 0 | - | 11.14 | 0 | 1 |
| LILRB1 | chr19 | 55144658 | A | G | NM_001081637 | c.1150A>G:p.M384V | Missense | - | - | - | 1.749 | 0 | 1 |
| LILRB1 | chr19 | 55144670 | A | G | NM_001081637 | c.1162A>G:p.T388A | Missense | - | - | - | 13.06 | 1 | 0 |
| LILRB1 | chr19 | 55144683 | C | T | NM_001081637 | c.1175C>T:p.A392V | Missense | 0 | - | 0 | 10.69 | 0 | 1 |
| LILRB1 | chr19 | 55144703 | G | A | NM_001081637 | c.1195G>A:p.G399S | Missense | 0 | - | 0 | 0.014 | 1 | 0 |
| LILRB1 | chr19 | 55144710 | A | T | NM_001081637 | c.1202A>T:p.Q401L | Missense | 0.0003 | 0 | 0.0001 | 0.001 | 1 | 4 |
| LILRB1 | chr19 | 55144711 | G | C | NM_001081637 | c.1203G>C:p.Q401H | Missense | 0.0003 | 0 | 0.0001 | 0.001 | 1 | 4 |
| LILRB1 | chr19 | 55144720 | A | C | NM_001081637 | c.1212A>C:p.K404N | Missense | 0.0003 | 0 | 0.0001 | 0.003 | 1 | 4 |
| LILRB1 | chr19 | 55144721 | C | T | NM_001081637 | c.1213C>T:p.P405S | Missense | - | - | - | 4.981 | 1 | 0 |
| LILRB1 | chr19 | 55144733 | A | T | NM_001081637 | c.1225A>T:p.T409S | Missense | 0.0013 | 0.0012 | 0.0015 | 0.001 | 3 | 3 |
| LILRB1 | chr19 | 55144734 | C | T | NM_001081637 | c.1226C>T:p.T409I | Missense | - | - | - | 13.38 | 2 | 1 |
| LILRB1 | chr19 | 55144754 | G | A | NM_001081637 | c.1246G>A:p.E416K | Missense | - | - | - | 17 | 0 | 1 |
| LILRB1 | chr19 | 55145092 | C | T | NM_001081637 | c.1265C>T:p.P422L | Missense | 0.0002 | 0 | 0.0002 | 13.89 | 1 | 0 |
| LILRB1 | chr19 | 55145113 | C | T | NM_001081637 | c.1286C>T:p.P429L | Missense | 0.0002 | 0 | 0.0002 | 17.04 | 1 | 1 |
| LILRB1 | chr19 | 55145139 | G | C | NM_001081637 | c.1312G>C:p.A438P | Missense | - | - | - | 0.069 | 1 | 0 |
| LILRB1 | chr19 | 55145421 | G | T | NM_001081637 | c.1313-1G>T | Splicing | 0 | - | 0 | 7.208 | 1 | 0 |
| LILRB1 | chr19 | 55145472 | G | A | NM_001081637 | c.1363G>A:p.G455S | Missense | 0 | - | 0.0004 | 2.084 | 1 | 2 |
| LILRB1 | chr19 | 55146148 | CTC | - | NM_001081637 | c.1420_1422del:p.L480del | NonFrameshift deletion | 0.0027 | 0.0025 | 0.0069 | - | 8 | 6 |
| LILRB1 | chr19 | 55146169 | T | C | NM_001081637 | c.1441T>C:p.F481L | Missense | 0 | - | 0 | 0.004 | 0 | 1 |
| LILRB1 | chr19 | 55146176 | T | C | NM_001081637 | c.1448T>C:p.I483T | Missense | 0 | - | - | 0.43 | 1 | 0 |
| LILRB1 | chr19 | 55146187 | C | T | NM_001081637 | c.1459C>T:p.R487X | Stopgain | 0 | - | 0 | 32 | 1 | 0 |
| LILRB1 | chr19 | 55146191 | G | A | NM_001081637 | c.1463G>A:p.R488H | Missense | 0 | - | 0 | 0.673 | 2 | 2 |
| LILRB1 | chr19 | 55146212 | C | T | NM_001081637 | c.1484C>T:p.S495L | Missense | 0 | 0 | 0 | 9.604 | 0 | 1 |
| LILRB1 | chr19 | 55146627 | G | A | NM_001081637 | c.1559G>A:p.W520X | Stopgain | 0.0002 | - | 0.0002 | 26.7 | 1 | 0 |
| LILRB1 | chr19 | 55146715 | G | A | NM_001081637 | c.1568G>A:p.S523N | Missense | - | - | - | 0.178 | 1 | 0 |
| LILRB1 | chr19 | 55146721 | C | G | NM_001081637 | c.1574C>G:p.A525G | Missense | - | - | - | 1.709 | 1 | 1 |
| LILRB1 | chr19 | 55147016 | G | A | NM_001081637 | c.1609G>A:p.V537M | Missense | 0 | - | 0 | 2.023 | 1 | 0 |
| LILRB1 | chr19 | 55147023 | A | G | NM_001081637 | c.1616A>G:p.H539R | Missense | 0.0003 | 0.0012 | 0.0003 | 2.591 | 0 | 1 |
| LILRB1 | chr19 | 55147058 | C | T | NM_001081637 | c.1651C>T:p.R551W | Missense | 5.80E-05 | 0 | 0.0001 | 23 | 0 | 1 |
| LILRB1 | chr19 | 55147987 | G | A | NM_001081637 | c.1696G>A:p.E566K | Missense | 0.001 | 0.002 | 0.0013 | 7.117 | 5 | 1 |
| LILRB1 | chr19 | 55148010 | G | C | NM_001081637 | c.1719G>C:p.R573S | Missense | - | - | - | 13.69 | 1 | 1 |
| LILRB1 | chr19 | 55148014 | G | T | NM_001081637 | c.1723G>T:p.E575X | Stopgain | 0 | - | 0 | 35 | 0 | 1 |
| LILRB1 | chr19 | 55148075 | C | T | NM_001081637 | c.1784C>T:p.A595V | Missense | 0 | 0 | 0 | 8.389 | 3 | 1 |
| LILRB1 | chr19 | 55148103 | G | - | NM_001081637 | c.1812delG:p.A605Lfs*9 | Frameshift deletion | 0.0001 | 0 | 0 | - | 0 | 1 |
| LILRB1 | chr19 | 55148105 | T | A | NM_001081637 | c.1812+2T>A | Splicing | 0.0001 | 0 | 0 | 0.081 | 1 | 1 |
| LILRB1 | chr19 | 55148201 | C | T | NM_001081637 | c.1831C>T:p.P611S | Missense | 0 | 0.0006 | 0 | 3.686 | 0 | 1 |
| LILRB1 | chr19 | 55148246 | C | T | NM_001081637 | c.1876C>T:p.R626W | Missense | 0 | 0 | 0 | 17.33 | 0 | 1 |
| LILRB1 | chr19 | 55148247 | G | A | NM_001081637 | c.1877G>A:p.R626Q | Missense | 0 | 0 | 0 | 5.065 | 0 | 1 |
| LILRB1 | chr19 | 55148253 | C | G | NM_001081637 | c.1883C>G:p.A628G | Missense | 0.0006 | 0.0012 | 0.0003 | 11.01 | 0 | 1 |
| RTN4R | chr22 | 20229281 | G | C | NM_023004 | c.1375C>G:p.P459A | Missense | 9.68E-05 | - | - | 1.134 | 0 | 2 |
| RTN4R | chr22 | 20229302 | T | C | NM_023004 | c.1354A>G:p.S452G | Missense | - | - | - | 0.008 | 2 | 0 |
| RTN4R | chr22 | 20229385 | C | T | NM_023004 | c.1271G>A:p.R424H | Missense | - | - | - | 25 | 1 | 0 |
| RTN4R | chr22 | 20229413 | G | C | NM_023004 | c.1243C>G:p.R415G | Missense | - | - | - | 23.9 | 1 | 0 |
| RTN4R | chr22 | 20229431 | G | C | NM_023004 | c.1225C>G:p.P409A | Missense | - | - | - | 4.008 | 0 | 1 |
| RTN4R | chr22 | 20229517 | T | C | NM_023004 | c.1139A>G:p.N380S | Missense | 0 | - | 0 | 17.48 | 0 | 1 |
| RTN4R | chr22 | 20229526 | C | T | NM_023004 | c.1130G>A:p.R377Q | Missense | 0.0008 | 0 | 0.001 | 23 | 0 | 2 |
| RTN4R | chr22 | 20229527 | G | A | NM_023004 | c.1129C>T:p.R377W | Missense | 0.0001 | 0 | 0.0001 | 24.2 | 0 | 1 |
| RTN4R | chr22 | 20229539 | C | T | NM_023004 | c.1117G>A:p.G373S | Missense | 0 | 0.0006 | 0 | 7.2 | 0 | 1 |
| RTN4R | chr22 | 20229547 | G | A | NM_023004 | c.1109C>T:p.P370L | Missense | 0 | - | 0 | 1.668 | 2 | 0 |
| RTN4R | chr22 | 20229569 | C | T | NM_023004 | c.1087G>A:p.V363M | Missense | 0.0032 | 0.0049 | 0.0027 | 10.12 | 16 | 8 |
| RTN4R | chr22 | 20229625 | G | T | NM_023004 | c.1031C>A:p.A344D | Missense | 0.0003 | - | 0.0001 | 13.59 | 1 | 0 |
| RTN4R | chr22 | 20229628 | T | G | NM_023004 | c.1028A>C:p.K343T | Missense | - | - | - | 23.8 | 1 | 0 |
| RTN4R | chr22 | 20229667 | A | T | NM_023004 | c.989T>A:p.L330Q | Missense | 5.83E-05 | - | 0.0001 | 1.492 | 2 | 0 |
| RTN4R | chr22 | 20229680 | C | T | NM_023004 | c.976G>A:p.D326N | Missense | 0.0001 | - | 0.0003 | 0.612 | 2 | 1 |
| RTN4R | chr22 | 20229881 | C | T | NM_023004 | c.775G>A:p.D259N | Missense | 0.0005 | - | 0.0005 | 13.08 | 2 | 2 |
| RTN4R | chr22 | 20229997 | T | C | NM_023004 | c.659A>G:p.H220R | Missense | 0 | - | 0 | 0.001 | 0 | 1 |
| RTN4R | chr22 | 20230000 | G | A | NM_023004 | c.656C>T:p.P219L | Missense | 5.80E-05 | - | 0.0001 | 20.2 | 0 | 1 |
| RTN4R | chr22 | 20230016 | C | T | NM_023004 | c.640G>A:p.V214M | Missense | 0.0001 | 0 | 0.0001 | 25.1 | 0 | 1 |
| RTN4R | chr22 | 20230025 | G | T | NM_023004 | c.631C>A:p.Q211K | Missense | 0 | - | - | 19.67 | 1 | 1 |
| RTN4R | chr22 | 20230070 | G | A | NM_023004 | c.586C>T:p.R196C | Missense | 0 | 0 | 0 | 28.8 | 0 | 1 |
| RTN4R | chr22 | 20230133 | G | A | NM_023004 | c.523C>T:p.R175C | Missense | 0.0003 | - | 0.0005 | 25.6 | 2 | 1 |
| RTN4R | chr22 | 20230140 | G | C | NM_023004 | c.516C>G:p.D172E | Missense | 0.0015 | 0.0006 | 0.0019 | 17.78 | 3 | 5 |
| RTN4R | chr22 | 20230163 | C | T | NM_023004 | c.493G>A:p.A165T | Missense | 0 | 0 | 0 | 10.71 | 1 | 0 |
| RTN4R | chr22 | 20230264 | C | T | NM_023004 | c.392G>A:p.R131H | Missense | 0.0003 | 0 | 0.0006 | 22.3 | 5 | 0 |
| RTN4R | chr22 | 20230267 | C | A | NM_023004 | c.389G>T:p.G130V | Missense | 0.0004 | - | 0.0002 | 13.3 | 4 | 6 |
| RTN4R | chr22 | 20230301 | G | A | NM_023004 | c.355C>T:p.R119W | Missense | 0 | 0 | 0 | 24.9 | 1 | 1 |
| RTN4R | chr22 | 20230363 | G | A | NM_023004 | c.293C>T:p.A98V | Missense | 0.0002 | 0.0006 | 0.0002 | 18.96 | 2 | 1 |
| RTN4R | chr22 | 20230372 | C | T | NM_023004 | c.284G>A:p.R95Q | Missense | 0.0005 | 0.0006 | 0.0006 | 4.29 | 0 | 1 |
| RTN4R | chr22 | 20230453 | C | T | NM_023004 | c.203G>A:p.R68H | Missense | 0.0017 | 0 | 0.0024 | 20.9 | 0 | 1 |
| RTN4R | chr22 | 20230499 | C | T | NM_023004 | c.157G>A:p.V53M | Missense | 0.0001 | 0 | 0.0001 | 0.033 | 2 | 2 |
| RTN4R | chr22 | 20230552 | T | C | NM_023004 | c.104A>G:p.N35S | Missense | 5.87E-05 | - | 0.0001 | 0.002 | 0 | 1 |
| RTN4R | chr22 | 20230568 | C | A | NM_023004 | c.88G>T:p.A30S | Missense | 0 | - | - | 2.538 | 1 | 0 |
| RTN4R | chr22 | 20230571 | C | G | NM_023004 | c.85G>C:p.G29R | Missense | - | - | - | 5.78 | 0 | 1 |
| RTN4R | chr22 | 20255606 | C | G | NM_023004 | c.10G>C:p.A4P | Missense | - | - | - | 18.19 | 1 | 0 |

**Supplementary Table 8 the rare variants of Nogo-related genes identified in the sLOPD cohort**

| **Gene** | **Chr** | **Position** | **Ref** | **Alt** | **NM_number** | **AAChangerefGene** | **Consequence** | **gnomAD_exome_EAS** | **gnomAD_genome_EAS** | **ExAC_EAS** | **CADD** | **WGS**  **(Case)** | **WGS**  **(Control)** |
| --- | --- | --- | --- | --- | --- | --- | --- | --- | --- | --- | --- | --- | --- |
| MTOR | chr1 | 11174503 | C | T | NM_004958 | c.7172G>A:p.G2391D | Missense | - | - | - | 33 | 1 | 0 |
| MTOR | chr1 | 11177116 | T | A | NM_004958 | c.6961A>T:p.T2321S | Missense | - | - | - | 24.5 | 0 | 1 |
| MTOR | chr1 | 11181369 | A | C | NM_004958 | c.6867T>G:p.H2289Q | Missense | - | - | - | 21 | 1 | 0 |
| MTOR | chr1 | 11187767 | C | T | NM_004958 | c.6130G>A:p.V2044M | Missense | 0 | 0 | - | 34 | 1 | 0 |
| MTOR | chr1 | 11189856 | C | T | NM_004958 | c.5653G>A:p.V1885I | Missense | 5.80E-05 | 0 | 0.0001 | 22.1 | 0 | 1 |
| MTOR | chr1 | 11190630 | C | T | NM_004958 | c.5569G>A:p.E1857K | Missense | 0 | - | 0 | 23.8 | 0 | 1 |
| MTOR | chr1 | 11190639 | C | T | NM_004958 | c.5560G>A:p.E1854K | Missense | 0 | - | 0 | 22.9 | 0 | 1 |
| MTOR | chr1 | 11190647 | C | T | NM_004958 | c.5552G>A:p.S1851N | Missense | - | - | - | 22.2 | 2 | 0 |
| MTOR | chr1 | 11190693 | C | T | NM_004958 | c.5506G>A:p.A1836T | Missense | 0.0003 | 0.0012 | 0.0005 | 12.62 | 1 | 0 |
| MTOR | chr1 | 11190695 | G | A | NM_004958 | c.5504C>T:p.A1835V | Missense | - | - | - | 22.3 | 0 | 1 |
| MTOR | chr1 | 11190765 | C | T | NM_004958 | c.5434G>A:p.D1812N | Missense | 0 | - | 0 | 22.4 | 1 | 0 |
| MTOR | chr1 | 11194424 | G | A | NM_004958 | c.5230C>T:p.H1744Y | Missense | 0.0002 | - | 0.0001 | 18.22 | 0 | 1 |
| MTOR | chr1 | 11199413 | T | G | NM_004958 | c.5078A>C:p.H1693P | Missense | - | - | - | 23.7 | 1 | 0 |
| MTOR | chr1 | 11199444 | G | A | NM_004958 | c.5047C>T:p.R1683W | Missense | 5.81E-05 | 0.0006 | - | 34 | 1 | 0 |
| MTOR | chr1 | 11199607 | C | T | NM_004958 | c.4981G>A:p.G1661S | Missense | 0 | - | 0 | 25.2 | 1 | 0 |
| MTOR | chr1 | 11210212 | C | T | NM_004958 | c.4541G>A:p.R1514Q | Missense | - | 0 | - | 32 | 1 | 0 |
| MTOR | chr1 | 11217266 | T | C | NM_004958 | c.4412A>G:p.K1471R | Missense | - | - | - | 22.7 | 2 | 0 |
| MTOR | chr1 | 11270894 | C | T | NM_004958 | c.3631G>A:p.V1211M | Missense | - | - | - | 22.4 | 1 | 0 |
| MTOR | chr1 | 11272380 | G | C | NM_004958 | c.3550C>G:p.L1184V | Missense | 0.0005 | - | 0.0003 | 23.2 | 1 | 2 |
| MTOR | chr1 | 11272449 | G | A | NM_004958 | c.3481C>T:p.R1161X | Stopgain | - | - | - | 43 | 1 | 0 |
| MTOR | chr1 | 11272469 | C | T | NM_004958 | c.3461G>A:p.R1154Q | Missense | 0 | - | 0 | 35 | 1 | 0 |
| MTOR | chr1 | 11272478 | T | C | NM_004958 | c.3452A>G:p.Y1151C | Missense | 0 | 0 | 0 | 24.8 | 0 | 1 |
| MTOR | chr1 | 11272529 | G | A | NM_004958 | c.3401C>T:p.A1134V | Missense | 0.0044 | 0.0056 | 0.0049 | 16.89 | 12 | 6 |
| MTOR | chr1 | 11273473 | G | A | NM_004958 | c.3268C>T:p.R1090C | Missense | 0.0001 | - | 0.0002 | 29.3 | 0 | 1 |
| MTOR | chr1 | 11273587 | T | C | NM_004958 | c.3154A>G:p.I1052V | Missense | 5.80E-05 | - | 0.0001 | 19.38 | 1 | 0 |
| MTOR | chr1 | 11288750 | C | T | NM_004958 | c.3005G>A:p.R1002Q | Missense | - | - | - | 24.7 | 0 | 1 |
| MTOR | chr1 | 11288778 | C | T | NM_004958 | c.2977G>A:p.V993I | Missense | 5.80E-05 | - | - | 17.13 | 1 | 0 |
| MTOR | chr1 | 11293472 | T | C | NM_004958 | c.2404A>G:p.I802V | Missense | 0.0001 | - | 0.0001 | 23.7 | 0 | 1 |
| MTOR | chr1 | 11297977 | C | A | NM_004958 | c.2131G>T:p.A711S | Missense | - | - | - | 33 | 1 | 0 |
| MTOR | chr1 | 11298591 | G | A | NM_004958 | c.1870C>T:p.R624C | Missense | 5.80E-05 | 0 | 0.0001 | 30 | 1 | 0 |
| MTOR | chr1 | 11298609 | T | C | NM_004958 | c.1852A>G:p.I618V | Missense | 5.80E-05 | - | 0.0001 | 19.32 | 1 | 0 |
| MTOR | chr1 | 11300603 | G | A | NM_004958 | c.1543C>T:p.P515S | Missense | - | - | - | 24.6 | 0 | 1 |
| MTOR | chr1 | 11307733 | T | C | NM_004958 | c.1174A>G:p.I392V | Missense | 0.0002 | - | 0.0003 | 10.14 | 1 | 2 |
| MTOR | chr1 | 11307923 | C | G | NM_004958 | c.1069G>C:p.V357L | Missense | - | - | - | 16.92 | 1 | 0 |
| MTOR | chr1 | 11307980 | A | G | NM_004958 | c.1012T>C:p.S338P | Missense | 0.0001 | 0 | 0.0001 | 18.4 | 1 | 0 |
| MTOR | chr1 | 11313946 | C | T | NM_004958 | c.790G>A:p.A264T | Missense | 5.80E-05 | - | - | 23.1 | 1 | 2 |
| MTOR | chr1 | 11316998 | G | A | NM_004958 | c.496C>T:p.H166Y | Missense | - | 0.0006 | - | 24 | 2 | 1 |
| MTOR | chr1 | 11317024 | C | T | NM_004958 | c.470G>A:p.G157D | Missense | - | - | - | 25.2 | 1 | 0 |
| MTOR | chr1 | 11319445 | C | T | NM_004958 | c.22G>A:p.A8T | Missense | 6.52E-05 | - | 0 | 23.3 | 0 | 1 |
| PLXNA2 | chr1 | 208200597 | A | C | NM_025179 | c.5676T>G:p.I1892M | Missense | 5.80E-05 | - | 0.0001 | 4.581 | 0 | 1 |
| PLXNA2 | chr1 | 208200649 | C | T | NM_025179 | c.5624G>A:p.R1875Q | Missense | 0.001 | 0.0006 | 0.001 | 27.2 | 4 | 1 |
| PLXNA2 | chr1 | 208200677 | C | G | NM_025179 | c.5596G>C:p.G1866R | Missense | 5.80E-05 | - | - | 23.5 | 1 | 0 |
| PLXNA2 | chr1 | 208206836 | G | A | NM_025179 | c.4883C>T:p.T1628M | Missense | 0 | 0 | 0.0002 | 34 | 1 | 0 |
| PLXNA2 | chr1 | 208211799 | C | A | NM_025179 | c.4681G>T:p.A1561S | Missense | 0.0001 | - | 0.0001 | 29.1 | 0 | 1 |
| PLXNA2 | chr1 | 208212193 | G | A | NM_025179 | c.4637C>T:p.P1546L | Missense | 0.0005 | - | 0.0006 | 34 | 0 | 1 |
| PLXNA2 | chr1 | 208212196 | C | T | NM_025179 | c.4634G>A:p.R1545Q | Missense | 0 | - | - | 35 | 1 | 0 |
| PLXNA2 | chr1 | 208212220 | T | C | NM_025179 | c.4610A>G:p.Y1537C | Missense | 0 | 0 | - | 26 | 1 | 0 |
| PLXNA2 | chr1 | 208212224 | C | A | NM_025179 | c.4606G>T:p.V1536L | Missense | - | - | - | 22.7 | 1 | 1 |
| PLXNA2 | chr1 | 208213045 | A | G | NM_025179 | c.4421T>C:p.I1474T | Missense | 0 | 0 | 0 | 27.6 | 1 | 0 |
| PLXNA2 | chr1 | 208213078 | C | T | NM_025179 | c.4388G>A:p.C1463Y | Missense | - | - | - | 26.5 | 1 | 0 |
| PLXNA2 | chr1 | 208213106 | C | T | NM_025179 | c.4360G>A:p.A1454T | Missense | 0 | - | - | 34 | 1 | 0 |
| PLXNA2 | chr1 | 208215573 | C | T | NM_025179 | c.4156G>A:p.V1386M | Missense | 0 | - | - | 34 | 0 | 1 |
| PLXNA2 | chr1 | 208215588 | G | A | NM_025179 | c.4141C>T:p.R1381C | Missense | - | - | - | 29.9 | 1 | 0 |
| PLXNA2 | chr1 | 208215644 | T | C | NM_025179 | c.4085A>G:p.K1362R | Missense | 0 | - | - | 24.6 | 0 | 1 |
| PLXNA2 | chr1 | 208215699 | C | T | NM_025179 | c.4030G>A:p.G1344R | Missense | 0 | - | 0 | 22.7 | 0 | 2 |
| PLXNA2 | chr1 | 208216416 | C | T | NM_025179 | c.4007G>A:p.R1336Q | Missense | 0.0003 | - | 0.0001 | 24.1 | 2 | 2 |
| PLXNA2 | chr1 | 208216548 | G | T | NM_025179 | c.3875C>A:p.A1292D | Missense | - | - | - | 31 | 0 | 1 |
| PLXNA2 | chr1 | 208218017 | G | T | NM_025179 | c.3710C>A:p.P1237Q | Missense | - | - | - | 29.3 | 1 | 1 |
| PLXNA2 | chr1 | 208218053 | G | A | NM_025179 | c.3674C>T:p.S1225L | Missense | 0 | - | 0 | 25.7 | 1 | 0 |
| PLXNA2 | chr1 | 208218068 | A | G | NM_025179 | c.3659T>C:p.V1220A | Missense | - | - | - | 17.81 | 3 | 0 |
| PLXNA2 | chr1 | 208218087 | C | T | NM_025179 | c.3640G>A:p.V1214I | Missense | - | - | - | 25.5 | 1 | 0 |
| PLXNA2 | chr1 | 208218540 | G | A | NM_025179 | c.3511C>T:p.P1171S | Missense | - | - | - | 26.3 | 1 | 0 |
| PLXNA2 | chr1 | 208219257 | AA | - | NM_025179 | c.3460_3461del:p.L1154Gfs*41 | Frameshift deletion | - | - | - | - | 0 | 1 |
| PLXNA2 | chr1 | 208219398 | C | T | NM_025179 | c.3320G>A:p.R1107H | Missense | 0 | 0 | 0 | 27.9 | 0 | 1 |
| PLXNA2 | chr1 | 208224737 | G | T | NM_025179 | c.3025C>A:p.P1009T | Missense | 0.0005 | 0.0006 | 0.0005 | 17.8 | 0 | 1 |
| PLXNA2 | chr1 | 208225786 | T | C | NM_025179 | c.2879A>G:p.N960S | Missense | 5.80E-05 | - | 0.0001 | 0.548 | 0 | 1 |
| PLXNA2 | chr1 | 208227830 | C | T | NM_025179 | c.2792G>A:p.R931H | Missense | 0.0021 | 0.0037 | 0.0022 | 22.8 | 12 | 8 |
| PLXNA2 | chr1 | 208227831 | G | A | NM_025179 | c.2791C>T:p.R931C | Missense | 0.0008 | 0.0006 | 0.0008 | 32 | 2 | 2 |
| PLXNA2 | chr1 | 208227855 | C | T | NM_025179 | c.2767G>A:p.V923M | Missense | 0.0002 | - | 0.0001 | 17.77 | 1 | 1 |
| PLXNA2 | chr1 | 208234173 | C | T | NM_025179 | c.2596G>A:p.V866M | Missense | 0 | - | - | 22.6 | 1 | 0 |
| PLXNA2 | chr1 | 208234175 | G | A | NM_025179 | c.2594C>T:p.T865M | Missense | 0 | 0 | - | 32 | 2 | 0 |
| PLXNA2 | chr1 | 208252680 | G | C | NM_025179 | c.2511C>G:p.H837Q | Missense | 5.80E-05 | - | - | 22.5 | 1 | 0 |
| PLXNA2 | chr1 | 208252702 | C | T | NM_025179 | c.2489G>A:p.R830H | Missense | 0 | - | 0 | 23.5 | 1 | 0 |
| PLXNA2 | chr1 | 208252735 | C | T | NM_025179 | c.2456G>A:p.R819Q | Missense | 0 | - | 0 | 23.3 | 1 | 0 |
| PLXNA2 | chr1 | 208252736 | G | A | NM_025179 | c.2455C>T:p.R819W | Missense | 0.0008 | - | 0.0008 | 26.4 | 2 | 2 |
| PLXNA2 | chr1 | 208252757 | C | T | NM_025179 | c.2434G>A:p.G812S | Missense | 0 | - | 0 | 31 | 0 | 1 |
| PLXNA2 | chr1 | 208255835 | C | T | NM_025179 | c.2317G>A:p.D773N | Missense | 0.0001 | 0 | 0 | 23.1 | 2 | 0 |
| PLXNA2 | chr1 | 208257869 | C | A | NM_025179 | c.2154G>T:p.K718N | Missense | - | - | - | 17.39 | 1 | 0 |
| PLXNA2 | chr1 | 208266216 | C | T | NM_025179 | c.2012G>A:p.R671H | Missense | 0 | - | 0 | 25.8 | 1 | 0 |
| PLXNA2 | chr1 | 208270124 | G | C | NM_025179 | c.1836C>G:p.I612M | Missense | 5.80E-05 | - | 0.0001 | 15.66 | 1 | 2 |
| PLXNA2 | chr1 | 208270138 | C | T | NM_025179 | c.1822G>A:p.G608R | Missense | 0.0005 | - | 0.0001 | 28.5 | 1 | 0 |
| PLXNA2 | chr1 | 208272219 | C | T | NM_025179 | c.1703G>A:p.S568N | Missense | 0.0003 | - | - | 11.43 | 2 | 0 |
| PLXNA2 | chr1 | 208272256 | T | C | NM_025179 | c.1666A>G:p.I556V | Missense | - | - | - | 14.86 | 1 | 0 |
| PLXNA2 | chr1 | 208276544 | C | T | NM_025179 | c.1555G>A:p.E519K | Missense | - | - | - | 23 | 1 | 0 |
| PLXNA2 | chr1 | 208276555 | G | A | NM_025179 | c.1544C>T:p.T515M | Missense | 0.0001 | - | 0.0001 | 25.2 | 0 | 1 |
| PLXNA2 | chr1 | 208315730 | G | A | NM_025179 | c.1450C>T:p.R484W | Missense | 0 | 0 | 0 | 33 | 1 | 0 |
| PLXNA2 | chr1 | 208315739 | G | A | NM_025179 | c.1441C>T:p.P481S | Missense | 0 | - | - | 24.5 | 1 | 0 |
| PLXNA2 | chr1 | 208315789 | G | C | NM_025179 | c.1391C>G:p.P464R | Missense | 5.88E-05 | - | - | 16.68 | 0 | 1 |
| PLXNA2 | chr1 | 208315804 | C | T | NM_025179 | c.1376G>A:p.R459Q | Missense | 5.95E-05 | - | - | 23.3 | 1 | 0 |
| PLXNA2 | chr1 | 208383740 | A | G | NM_025179 | c.1256T>C:p.V419A | Missense | - | - | - | 27.4 | 1 | 0 |
| PLXNA2 | chr1 | 208383744 | G | T | NM_025179 | c.1252C>A:p.P418T | Missense | - | - | - | 15.8 | 0 | 1 |
| PLXNA2 | chr1 | 208390097 | C | T | NM_025179 | c.1171G>A:p.V391I | Missense | 0 | - | 0 | 23.7 | 0 | 1 |
| PLXNA2 | chr1 | 208390102 | T | G | NM_025179 | c.1166A>C:p.K389T | Missense | - | - | - | 23.6 | 4 | 6 |
| PLXNA2 | chr1 | 208390186 | C | T | NM_025179 | c.1082G>A:p.R361Q | Missense | 0.0004 | 0.0006 | 0.0001 | 21.1 | 4 | 4 |
| PLXNA2 | chr1 | 208390187 | G | A | NM_025179 | c.1081C>T:p.R361W | Missense | 0 | - | 0 | 33 | 1 | 0 |
| PLXNA2 | chr1 | 208390358 | C | T | NM_025179 | c.910G>A:p.G304R | Missense | 0.0003 | 0 | 0.0006 | 24.2 | 3 | 0 |
| PLXNA2 | chr1 | 208390487 | C | G | NM_025179 | c.781G>C:p.E261Q | Missense | - | - | 0.0001 | 18.89 | 1 | 0 |
| PLXNA2 | chr1 | 208390604 | C | G | NM_025179 | c.664G>C:p.V222L | Missense | - | - | - | 25.4 | 1 | 0 |
| PLXNA2 | chr1 | 208390637 | C | T | NM_025179 | c.631G>A:p.A211T | Missense | - | - | - | 17.65 | 1 | 0 |
| PLXNA2 | chr1 | 208390762 | T | C | NM_025179 | c.506A>G:p.Y169C | Missense | - | - | - | 14.82 | 1 | 0 |
| PLXNA2 | chr1 | 208390903 | G | T | NM_025179 | c.365C>A:p.S122Y | Missense | 5.80E-05 | 0.0012 | 0 | 23.4 | 1 | 0 |
| PLXNA2 | chr1 | 208391027 | C | G | NM_025179 | c.241G>C:p.V81L | Missense | - | - | - | 23.3 | 0 | 1 |
| PLXNA2 | chr1 | 208391084 | C | T | NM_025179 | c.184G>A:p.V62I | Missense | 0.0003 | 0.0006 | 0.0002 | 23.5 | 1 | 0 |
| PLXNA2 | chr1 | 208391092 | G | A | NM_025179 | c.176C>T:p.T59M | Missense | 0.0001 | 0.0012 | 0.0001 | 24.3 | 1 | 0 |
| PLXNA2 | chr1 | 208391126 | A | C | NM_025179 | c.142T>G:p.W48G | Missense | - | - | - | 23.6 | 0 | 1 |
| PLXNA2 | chr1 | 208391189 | G | T | NM_025179 | c.79C>A:p.L27M | Missense | - | - | - | 9.777 | 0 | 1 |
| PLXNA2 | chr1 | 208391192 | C | A | NM_025179 | c.76G>T:p.V26L | Missense | 0.0006 | 0.0012 | 0.0006 | 9.567 | 2 | 0 |
| PLXNA2 | chr1 | 208391236 | A | G | NM_025179 | c.32T>C:p.L11P | Missense | 0.0012 | 0.0019 | 0.0011 | 0.001 | 2 | 0 |
| RTN4 | chr2 | 55200306 | G | A | NM_020532 | c.3565C>T:p.R1189C | Missense | 0.0009 | 0.0006 | 0.0007 | 28.4 | 0 | 1 |
| RTN4 | chr2 | 55200727 | T | G | NM_020532 | c.3508A>C:p.N1170H | Missense | - | - | - | 26.9 | 0 | 1 |
| RTN4 | chr2 | 55209706 | G | A | NM_020532 | c.3305C>T:p.T1102M | Missense | 0.0009 | 0.0012 | 0.0007 | 32 | 4 | 1 |
| RTN4 | chr2 | 55209764 | T | C | NM_020532 | c.3247A>G:p.I1083V | Missense | 0.0004 | - | 0.0001 | 4.276 | 1 | 0 |
| RTN4 | chr2 | 55214692 | G | C | NM_020532 | c.3156C>G:p.I1052M | Missense | 0.0004 | - | 0.0001 | 27.6 | 3 | 0 |
| RTN4 | chr2 | 55252326 | G | A | NM_020532 | c.2909C>T:p.P970L | Missense | 0.0003 | - | 0.0002 | 23.5 | 2 | 1 |
| RTN4 | chr2 | 55252342 | C | T | NM_020532 | c.2893G>A:p.E965K | Missense | - | - | - | 19.99 | 0 | 1 |
| RTN4 | chr2 | 55252404 | G | A | NM_020532 | c.2831C>T:p.A944V | Missense | 0 | - | 0 | 9.641 | 1 | 0 |
| RTN4 | chr2 | 55252447 | C | T | NM_020532 | c.2788G>A:p.E930K | Missense | 0.0038 | 0.0025 | 0.0035 | 23.4 | 12 | 6 |
| RTN4 | chr2 | 55252452 | A | C | NM_020532 | c.2783T>G:p.V928G | Missense | - | - | 0.0001 | 0.015 | 0 | 1 |
| RTN4 | chr2 | 55252524 | G | A | NM_020532 | c.2711C>T:p.P904L | Missense | 0.0002 | 0.0006 | 0.0001 | 12.78 | 0 | 1 |
| RTN4 | chr2 | 55252596 | T | A | NM_020532 | c.2639A>T:p.D880V | Missense | 0 | - | 0 | 13.05 | 2 | 0 |
| RTN4 | chr2 | 55252705 | C | T | NM_020532 | c.2530G>A:p.D844N | Missense | - | - | - | 18.69 | 1 | 0 |
| RTN4 | chr2 | 55252734 | T | A | NM_020532 | c.2501A>T:p.E834V | Missense | 0.0001 | 0 | 0.0002 | 25.7 | 1 | 0 |
| RTN4 | chr2 | 55252743 | T | C | NM_020532 | c.2492A>G:p.Q831R | Missense | - | - | - | 24.4 | 1 | 0 |
| RTN4 | chr2 | 55252770 | A | C | NM_020532 | c.2465T>G:p.L822W | Missense | - | - | - | 12.2 | 0 | 1 |
| RTN4 | chr2 | 55252804 | T | C | NM_020532 | c.2431A>G:p.K811E | Missense | 0 | - | 0 | 14.04 | 2 | 1 |
| RTN4 | chr2 | 55252874 | T | G | NM_020532 | c.2361A>C:p.K787N | Missense | - | - | - | 8.347 | 0 | 1 |
| RTN4 | chr2 | 55253157 | G | A | NM_020532 | c.2078C>T:p.P693L | Missense | 0.0002 | - | 0.0002 | 23.7 | 0 | 1 |
| RTN4 | chr2 | 55253269 | G | C | NM_020532 | c.1966C>G:p.P656A | Missense | 0.0001 | - | 0.0001 | 24 | 1 | 0 |
| RTN4 | chr2 | 55253409 | G | A | NM_020532 | c.1826C>T:p.P609L | Missense | 0.0004 | 0.0019 | 0.0005 | 26.8 | 2 | 3 |
| RTN4 | chr2 | 55253560 | A | G | NM_020532 | c.1675T>C:p.C559R | Missense | - | - | - | 22.9 | 0 | 1 |
| RTN4 | chr2 | 55253831 | G | T | NM_020532 | c.1404C>A:p.N468K | Missense | - | - | - | 9.577 | 1 | 0 |
| RTN4 | chr2 | 55253851 | T | C | NM_020532 | c.1384A>G:p.I462V | Missense | - | - | - | 23.3 | 2 | 0 |
| RTN4 | chr2 | 55253886 | G | A | NM_020532 | c.1349C>T:p.T450M | Missense | 0.0002 | 0 | 0.0002 | 27.4 | 3 | 0 |
| RTN4 | chr2 | 55253920 | C | G | NM_020532 | c.1315G>C:p.E439Q | Missense | - | - | - | 25.4 | 1 | 1 |
| RTN4 | chr2 | 55254078 | T | A | NM_020532 | c.1157A>T:p.D386V | Missense | - | - | - | 25.9 | 0 | 1 |
| RTN4 | chr2 | 55254217 | G | C | NM_020532 | c.1018C>G:p.L340V | Missense | - | - | - | 0.004 | 1 | 0 |
| RTN4 | chr2 | 55254286 | C | G | NM_020532 | c.949G>C:p.A317P | Missense | 5.81E-05 | - | - | 0.038 | 1 | 1 |
| RTN4 | chr2 | 55254313 | G | T | NM_020532 | c.922C>A:p.P308T | Missense | 5.81E-05 | - | 0 | 11.16 | 0 | 1 |
| RTN4 | chr2 | 55254372 | T | C | NM_020532 | c.863A>G:p.D288G | Missense | 0.003 | 0.0006 | 0.0014 | 12.86 | 13 | 8 |
| RTN4 | chr2 | 55254397 | C | T | NM_020532 | c.838G>A:p.A280T | Missense | 0.0002 | - | 0.0002 | 23.3 | 0 | 1 |
| RTN4 | chr2 | 55254514 | G | A | NM_020532 | c.721C>T:p.L241F | Missense | 0 | - | 0 | 22.8 | 0 | 1 |
| RTN4 | chr2 | 55254580 | C | T | NM_020532 | c.655G>A:p.A219T | Missense | - | - | - | 6.875 | 1 | 0 |
| RTN4 | chr2 | 55255312 | G | A | NM_020532 | c.601C>T:p.R201C | Missense | 5.82E-05 | - | 0 | 26.1 | 1 | 0 |
| RTN4 | chr2 | 55276928 | G | A | NM_020532 | c.509C>T:p.P170L | Missense | 0 | 0 | 0 | 27.3 | 0 | 1 |
| RTN4 | chr2 | 55277026 | G | T | NM_020532 | c.411C>A:p.D137E | Missense | - | - | - | 13.12 | 1 | 0 |
| RTN4 | chr2 | 55277055 | C | A | NM_020532 | c.382G>T:p.V128F | Missense | - | - | - | 22.6 | 0 | 1 |
| RTN4 | chr2 | 55277061 | C | T | NM_020532 | c.376G>A:p.A126T | Missense | - | - | - | 11.76 | 2 | 0 |
| RTN4 | chr2 | 55277130 | C | G | NM_020532 | c.307G>C:p.E103Q | Missense | - | - | - | 22.3 | 1 | 0 |
| RTN4 | chr2 | 55277141 | G | A | NM_020532 | c.296C>T:p.P99L | Missense | 0.0011 | 0.0025 | 0.0044 | 11.4 | 5 | 3 |
| RTN4 | chr2 | 55277153 | G | A | NM_020532 | c.284C>T:p.P95L | Missense | - | - | - | 29.7 | 1 | 0 |
| RTN4 | chr2 | 55277181 | C | G | NM_020532 | c.256G>C:p.V86L | Missense | 0 | - | - | 22.8 | 1 | 0 |
| RTN4 | chr2 | 55277315 | TCC | - | NM_020532 | c.120_122del:p.E43del | NonFrameshift deletion | 0 | - | - | - | 0 | 1 |
| RTN4 | chr2 | 55277334 | C | T | NM_020532 | c.103G>A:p.E35K | Missense | 0 | - | - | 24.2 | 1 | 0 |
| RTN4 | chr2 | 55277436 | T | C | NM_020532 | c.1A>G:p.M1V | Missense | 0 | 0 | 0 | 21.5 | 0 | 1 |
| RTN4 | chr2 | 55307774 | C | G |  |  | Splicing | - | - | - | - | 3 | 0 |
| ITGAV | chr2 | 187455160 | A | C | NM_001145000 | c.95A>C:p.N32T | Missense | - | - | - | 28.8 | 0 | 1 |
| ITGAV | chr2 | 187466749 | C | T | NM_001145000 | c.187C>T:p.R63W | Missense | 0 | 0 | 0 | 33 | 0 | 1 |
| ITGAV | chr2 | 187466845 | C | T | NM_001145000 | c.283C>T:p.R95C | Missense | 0 | 0 | 0 | 26 | 0 | 1 |
| ITGAV | chr2 | 187466849 | G | A | NM_001145000 | c.287G>A:p.R96Q | Missense | 0.0013 | 0.0012 | 0.0015 | 16.98 | 2 | 3 |
| ITGAV | chr2 | 187500841 | G | A | NM_001145000 | c.556G>A:p.V186I | Missense | 0.0009 | 0.0006 | 0.0008 | 2.901 | 2 | 1 |
| ITGAV | chr2 | 187506129 | A | T | NM_001145000 | c.865A>T:p.I289F | Missense | 0.0007 | 0.0025 | 0.0002 | 26.8 | 0 | 2 |
| ITGAV | chr2 | 187506154 | G | A | NM_001145000 | c.890G>A:p.R297H | Missense | 0 | - | - | 34 | 1 | 0 |
| ITGAV | chr2 | 187506210 | G | A | NM_001145000 | c.946G>A:p.A316T | Missense | 0.0002 | 0.0006 | - | 15.53 | 2 | 3 |
| ITGAV | chr2 | 187506261 | C | T | NM_001145000 | c.997C>T:p.R333W | Missense | - | - | - | 34 | 1 | 0 |
| ITGAV | chr2 | 187516797 | A | G | NM_001145000 | c.1378A>G:p.T460A | Missense | 0.0001 | 0.0006 | - | 20.8 | 1 | 1 |
| ITGAV | chr2 | 187519408 | G | A | NM_001145000 | c.1429G>A:p.G477S | Missense | 0.0002 | - | 0.0002 | 31 | 4 | 1 |
| ITGAV | chr2 | 187521047 | C | G | NM_001145000 | c.1530C>G:p.S510R | Missense | - | - | - | 26.5 | 1 | 0 |
| ITGAV | chr2 | 187521057 | A | G | NM_001145000 | c.1540A>G:p.S514G | Missense | 0.0008 | 0.0006 | 0.001 | 9.346 | 2 | 1 |
| ITGAV | chr2 | 187521087 | G | C | NM_001145000 | c.1570G>C:p.G524R | Missense | 9.16E-05 | 0 | 0 | 22.8 | 2 | 0 |
| ITGAV | chr2 | 187529241 | T | C | NM_001145000 | c.1838T>C:p.I613T | Missense | 0.0005 | 0.0006 | 0.0005 | 28.4 | 1 | 0 |
| ITGAV | chr2 | 187529348 | G | A | NM_001145000 | c.1945G>A:p.G649R | Missense | 0 | 0 | 0 | 34 | 0 | 1 |
| ITGAV | chr2 | 187529871 | T | G | NM_001145000 | c.1984T>G:p.C662G | Missense | 5.85E-05 | - | 0.0001 | 29.8 | 1 | 0 |
| ITGAV | chr2 | 187529907 | G | A | NM_001145000 | c.2020G>A:p.V674I | Missense | 0.0001 | - | 0.0001 | 25.3 | 1 | 0 |
| ITGAV | chr2 | 187533499 | A | G | NM_001145000 | c.2336A>G:p.N779S | Missense | 0.0028 | 0.0012 | 0.0023 | 14.89 | 4 | 5 |
| ITGAV | chr2 | 187534462 | C | T | NM_001145000 | c.2519C>T:p.T840M | Missense | 0 | 0 | 0 | 22.2 | 1 | 0 |
| ITGAV | chr2 | 187540374 | G | A | NM_001145000 | c.2642G>A:p.G881E | Missense | 0 | 0 | 0 | 27.7 | 1 | 0 |
| ITGAV | chr2 | 187540581 | C | T | NM_001145000 | c.2747C>T:p.S916L | Missense | 0 | - | 0 | 32 | 0 | 1 |
| ITGAV | chr2 | 187541658 | A | G | NM_001145000 | c.2939A>G:p.Y980C | Missense | 5.80E-05 | - | - | 27.2 | 1 | 0 |
| RHOA | chr3 | 49397650 | C | T | NM_001313946 | c.322G>A:p.V108I | Missense | 0 | - | 0 | 9.337 | 0 | 1 |
| RHOA | chr3 | 49398367 | G | C | NM_001313943 | c.541C>G:p.H181D | Missense | - | - | - | 0.089 | 0 | 1 |
| RHOA | chr3 | 49398409 | G | A | NM_001313943 | c.499C>T:p.L167F | Missense | - | - | - | 3.93 | 1 | 0 |
| RHOA | chr3 | 49398491 | G | T | NM_001313943 | c.417C>A:p.H139Q | Missense | - | - | - | 0.261 | 1 | 2 |
| PIK3CB | chr3 | 138376533 | G | A | NM_006219 | c.2941C>T:p.R981W | Missense | 0.0002 | 0.0006 | - | 34 | 1 | 0 |
| PIK3CB | chr3 | 138376569 | G | C | NM_006219 | c.2905C>G:p.Q969E | Missense | - | - | - | 24.3 | 0 | 1 |
| PIK3CB | chr3 | 138382857 | C | A | NM_006219 | c.2687G>T:p.R896L | Missense | 0.0035 | 0.0019 | 0.004 | 35 | 9 | 8 |
| PIK3CB | chr3 | 138402580 | C | T | NM_006219 | c.2365G>A:p.V789I | Missense | 0.0003 | - | 0.0006 | 24.5 | 1 | 0 |
| PIK3CB | chr3 | 138403527 | C | A | NM_006219 | c.2255G>T:p.R752L | Missense | 0.0003 | - | - | 21.7 | 2 | 1 |
| PIK3CB | chr3 | 138403528 | G | A | NM_006219 | c.2254C>T:p.R752W | Missense | 0 | - | 0 | 27.9 | 0 | 1 |
| PIK3CB | chr3 | 138407735 | C | T | NM_006219 | c.2118G>A:p.M706I | Missense | - | - | - | 22.7 | 1 | 0 |
| PIK3CB | chr3 | 138407788 | C | T | NM_006219 | c.2065G>A:p.V689I | Missense | 0 | - | - | 10.2 | 0 | 1 |
| PIK3CB | chr3 | 138426114 | A | C | NM_006219 | c.1417T>G:p.L473V | Missense | 0.0001 | - | 0.0001 | 25.4 | 1 | 0 |
| PIK3CB | chr3 | 138431133 | G | A | NM_006219 | c.1316C>T:p.A439V | Missense | - | - | - | 32 | 2 | 0 |
| PIK3CB | chr3 | 138433432 | A | C | NM_006219 | c.1180T>G:p.L394V | Missense | 0.0002 | 0.0006 | 0.0002 | 21.6 | 2 | 0 |
| PIK3CB | chr3 | 138433525 | G | A | NM_006219 | c.1087C>T:p.L363F | Missense | - | - | - | 20.1 | 0 | 1 |
| PIK3CB | chr3 | 138452259 | G | C | NM_006219 | c.994C>G:p.P332A | Missense | - | - | - | 18.82 | 0 | 1 |
| PIK3CB | chr3 | 138453502 | G | A | NM_006219 | c.946C>T:p.P316S | Missense | - | - | - | 23.2 | 1 | 0 |
| PIK3CB | chr3 | 138461564 | G | A | NM_006219 | c.457C>T:p.R153C | Missense | 0.0003 | - | 0.0003 | 22.9 | 1 | 0 |
| PIK3CB | chr3 | 138474810 | C | A | NM_006219 | c.183G>T:p.K61N | Missense | - | - | - | 23.8 | 0 | 1 |
| PIK3CA | chr3 | 178917486 | A | G | NM_006218 | c.361A>G:p.I121V | Missense | 0 | 0 | 0 | 5.172 | 0 | 1 |
| PIK3CA | chr3 | 178917595 | A | G | NM_006218 | c.470A>G:p.N157S | Missense | 5.80E-05 | - | - | 4.343 | 1 | 0 |
| PIK3CA | chr3 | 178921402 | C | T | NM_006218 | c.884C>T:p.S295F | Missense | 0 | - | - | 24.3 | 1 | 0 |
| PIK3CA | chr3 | 178937462 | G | A | NM_006218 | c.1850G>A:p.R617Q | Missense | 0.0002 | - | 0.0001 | 23 | 0 | 2 |
| PIK3CA | chr3 | 178941879 | A | G | NM_006218 | c.2198A>G:p.K733R | Missense | 0.0058 | 0.0062 | 0.0059 | 22.4 | 9 | 5 |
| PIK3CA | chr3 | 178943810 | A | G | NM_006218 | c.2477A>G:p.N826S | Missense | - | - | - | 13.97 | 0 | 1 |
| PIK3CA | chr3 | 178947792 | A | G | NM_006218 | c.2667A>G:p.I889M | Missense | 0.0003 | - | 0.0005 | 9.645 | 1 | 0 |
| LIMK1 | chr7 | 73500083 | G | A | NM_002314 | c.61G>A:p.E21K | Missense | 0.0002 | 0 | 0 | 16.89 | 0 | 2 |
| LIMK1 | chr7 | 73507610 | C | T | NM_001204426 | c.31C>T:p.R11C | Missense | 0 | 0.0006 | - | 7.703 | 0 | 1 |
| LIMK1 | chr7 | 73507610 | C | G | NM_001204426 | c.31C>G:p.R11G | Missense | 0 | 0 | - | 5.542 | 1 | 0 |
| LIMK1 | chr7 | 73510965 | A | C | NM_001204426 | c.64A>C:p.S22R | Missense | 0.0019 | 0.0031 | 0.0019 | 14.53 | 1 | 0 |
| LIMK1 | chr7 | 73511043 | G | A | NM_001204426 | c.142G>A:p.E48K | Missense | 0.0003 | 0.0012 | 0.0002 | 34 | 1 | 0 |
| LIMK1 | chr7 | 73511044 | A | G | NM_001204426 | c.143A>G:p.E48G | Missense | - | - | - | 23 | 0 | 2 |
| LIMK1 | chr7 | 73511465 | C | A | NM_001204426 | c.245C>A:p.T82N | Missense | 0 | 0.0006 | - | 14.48 | 1 | 1 |
| LIMK1 | chr7 | 73511492 | C | T | NM_001204426 | c.272C>T:p.T91M | Missense | 0 | 0 | 0 | 25.6 | 2 | 0 |
| LIMK1 | chr7 | 73520213 | C | T | NM_001204426 | c.515C>T:p.P172L | Missense | 0.0002 | 0 | 0.0002 | 23 | 0 | 1 |
| LIMK1 | chr7 | 73520243 | C | G | NM_001204426 | c.545C>G:p.S182C | Missense | - | - | - | 28.3 | 1 | 0 |
| LIMK1 | chr7 | 73520435 | G | A | NM_001204426 | c.641G>A:p.R214H | Missense | 0.001 | 0 | 0.0012 | 23.7 | 4 | 4 |
| LIMK1 | chr7 | 73520543 | C | T | NM_001204426 | c.749C>T:p.A250V | Missense | 0.001 | 0.0012 | 0.0009 | 8.774 | 2 | 1 |
| LIMK1 | chr7 | 73520557 | C | T | NM_001204426 | c.763C>T:p.R255W | Missense | 0.0001 | - | 0.0001 | 32 | 1 | 0 |
| LIMK1 | chr7 | 73520558 | G | A | NM_001204426 | c.764G>A:p.R255Q | Missense | 0 | 0.0006 | 0 | 22.4 | 1 | 2 |
| LIMK1 | chr7 | 73521402 | G | A | NM_001204426 | c.842G>A:p.R281H | Missense | 0.0015 | 0.0006 | 0.0006 | 19.88 | 6 | 4 |
| LIMK1 | chr7 | 73521468 | C | T | NM_001204426 | c.908C>T:p.S303L | Missense | 0.0003 | 0.0006 | 0 | 26.6 | 0 | 1 |
| LIMK1 | chr7 | 73522258 | G | A | NM_001204426 | c.1021G>A:p.E341K | Missense | 7.47E-05 | - | - | 33 | 1 | 0 |
| LIMK1 | chr7 | 73523247 | C | T | NM_001204426 | c.1063C>T:p.R355X | Stopgain | - | - | - | 38 | 0 | 1 |
| LIMK1 | chr7 | 73530286 | A | G | NM_001204426 | c.1463A>G:p.N488S | Missense | 5.80E-05 | - | 0.0001 | 23.3 | 3 | 1 |
| LIMK1 | chr7 | 73535524 | G | A | NM_001204426 | c.1735G>A:p.G579S | Missense | 5.83E-05 | - | 0 | 12.79 | 1 | 0 |
| LIMK1 | chr7 | 73535585 | G | A | NM_001204426 | c.1796G>A:p.R599Q | Missense | 0 | - | 0 | 22.6 | 1 | 0 |
| DPYSL2 | chr8 | 26372058 | C | T | NM_001197293 | c.217C>T:p.H73Y | Missense | 0.0007 | - | - | - | 3 | 3 |
| DPYSL2 | chr8 | 26372073 | C | T | NM_001197293 | c.232C>T:p.P78S | Missense | - | - | - | - | 1 | 0 |
| DPYSL2 | chr8 | 26439491 | C | G | NM_001197293 | c.361C>G:p.R121G | Missense | - | - | - | 33 | 0 | 1 |
| DPYSL2 | chr8 | 26439515 | A | C | NM_001197293 | c.385A>C:p.I129L | Missense | - | - | - | 26.5 | 2 | 0 |
| DPYSL2 | chr8 | 26484172 | G | A | NM_001197293 | c.833G>A:p.R278H | Missense | 0.0038 | 0.0012 | 0.0031 | 22.8 | 19 | 4 |
| DPYSL2 | chr8 | 26484184 | C | T | NM_001197293 | c.845C>T:p.T282M | Missense | 0 | 0 | 0 | 34 | 0 | 1 |
| DPYSL2 | chr8 | 26485419 | C | T | NM_001197293 | c.968C>T:p.T323M | Missense | 0.0001 | - | 0.0001 | 34 | 1 | 0 |
| DPYSL2 | chr8 | 26501524 | G | A | NM_001197293 | c.1342G>A:p.V448I | Missense | 0.0002 | 0 | 0.0001 | 19.52 | 1 | 0 |
| DPYSL2 | chr8 | 26501590 | A | G | NM_001197293 | c.1408A>G:p.I470V | Missense | - | - | - | 8.313 | 0 | 1 |
| DPYSL2 | chr8 | 26505233 | C | T | NM_001197293 | c.1513C>T:p.R505C | Missense | 0 | 0 | 0 | 34 | 1 | 0 |
| DPYSL2 | chr8 | 26509809 | C | T | NM_001197293 | c.1633C>T:p.R545C | Missense | 0.0001 | - | 0.0001 | 35 | 0 | 1 |
| NTRK2 | chr9 | 87285694 | G | A | NM_001018064 | c.31G>A:p.A11T | Missense | 0.0002 | - | 0.0001 | 22.7 | 1 | 2 |
| NTRK2 | chr9 | 87285754 | G | T | NM_001018064 | c.91G>T:p.A31S | Missense | 0 | - | - | 23.2 | 1 | 0 |
| NTRK2 | chr9 | 87285820 | G | A | NM_001018064 | c.157G>A:p.V53M | Missense | 0.0002 | - | 0.0003 | 19.52 | 0 | 1 |
| NTRK2 | chr9 | 87285872 | A | C | NM_001018064 | c.209A>C:p.E70A | Missense | - | - | - | 26.7 | 1 | 0 |
| NTRK2 | chr9 | 87322782 | C | T | NM_001018064 | c.383C>T:p.T128M | Missense | 5.80E-05 | - | 0.0001 | 25.5 | 1 | 0 |
| NTRK2 | chr9 | 87322806 | G | A | NM_001018064 | c.407G>A:p.R136H | Missense | 0.0002 | - | 0.0001 | 22.8 | 2 | 0 |
| NTRK2 | chr9 | 87322823 | G | A | NM_001018064 | c.424G>A:p.E142K | Missense | - | - | - | 23.3 | 1 | 0 |
| NTRK2 | chr9 | 87338488 | G | T | NM_001018064 | c.584G>T:p.G195V | Missense | 0.0001 | - | 0.0001 | 19.37 | 1 | 0 |
| NTRK2 | chr9 | 87342656 | C | T | NM_001018064 | c.941C>T:p.A314V | Missense | 0.0001 | - | 0.0002 | 23.3 | 1 | 0 |
| NTRK2 | chr9 | 87342675 | C | G | NM_001018064 | c.960C>G:p.N320K | Missense | 5.80E-05 | - | - | 6.404 | 0 | 1 |
| NTRK2 | chr9 | 87359974 | C | T | NM_001018064 | c.1282C>T:p.R428W | Missense | 0 | - | - | 35 | 1 | 0 |
| NTRK2 | chr9 | 87482242 | C | T | NM_001018064 | c.1481C>T:p.S494L | Missense | - | - | - | 26.4 | 0 | 1 |
| NTRK2 | chr9 | 87482254 | C | T | NM_001018064 | c.1493C>T:p.P498L | Missense | - | - | - | 25.7 | 0 | 1 |
| NTRK2 | chr9 | 87549082 | C | G | NM_001018064 | c.1591C>G:p.Q531E | Missense | - | - | - | 23.4 | 0 | 1 |
| NTRK2 | chr9 | 87563404 | C | T | NM_001018064 | c.1744C>T:p.R582C | Missense | - | - | - | 35 | 1 | 0 |
| NTRK2 | chr9 | 87636351 | A | C | NM_001018064 | c.2468A>C:p.X823S | stoploss | - | - | - | 13.83 | 3 | 5 |
| AKT1 | chr14 | 105236689 | C | T | NM_001014431 | c.1432G>A:p.G478S | Missense | 0 | - | - | 28.5 | 1 | 0 |
| AKT1 | chr14 | 105236728 | G | C | NM_001014431 | c.1393C>G:p.R465G | Missense | 5.84E-05 | 0.0006 | 0.0001 | 25 | 1 | 0 |
| AKT1 | chr14 | 105239614 | C | T | NM_001014431 | c.931G>A:p.G311S | Missense | - | - | - | 27.7 | 0 | 1 |
| AKT1 | chr14 | 105239875 | G | A | NM_001014431 | c.745C>T:p.R249W | Missense | 0 | - | - | 34 | 1 | 0 |
| AKT1 | chr14 | 105241428 | T | G | NM_001014431 | c.552A>C:p.E184D | Missense | - | - | - | 15.63 | 1 | 0 |
| AKT1 | chr14 | 105242019 | C | G | NM_001014431 | c.405G>C:p.E135D | Missense | - | - | - | 15.84 | 2 | 0 |
| AKT1 | chr14 | 105242073 | CTC | - | NM_001014431 | c.349_351del:p.E117del | NonFrameshift deletion | 0 | 0 | 0 | - | 1 | 0 |
| AKT1 | chr14 | 105243058 | G | C | NM_001014431 | c.225C>G:p.I75M | Missense | 0.0003 | - | 0.0002 | 24.8 | 0 | 1 |
| AKT1 | chr14 | 105243083 | C | T | NM_001014431 | c.200G>A:p.R67Q | Missense | - | - | - | 27.9 | 0 | 1 |
| AKT1 | chr14 | 105259547 | C | T |  |  | Splicing | - | - | - | - | 1 | 0 |
| LINGO1 | chr15 | 77906630 | T | A | NM_032808 | c.1619A>T:p.E540V | Missense | - | - | - | 15.16 | 1 | 0 |
| LINGO1 | chr15 | 77906753 | G | A | NM_032808 | c.1496C>T:p.A499V | Missense | 0 | 0 | - | 28.2 | 1 | 0 |
| LINGO1 | chr15 | 77906864 | C | T | NM_032808 | c.1385G>A:p.R462Q | Missense | 0 | 0 | 0 | 14.6 | 0 | 1 |
| LINGO1 | chr15 | 77906909 | C | T | NM_032808 | c.1340G>A:p.R447Q | Missense | 0.0003 | - | 0 | 15.18 | 0 | 1 |
| LINGO1 | chr15 | 77907254 | C | T | NM_032808 | c.995G>A:p.R332H | Missense | 0 | 0.0006 | 0.0001 | 24.6 | 0 | 1 |
| LINGO1 | chr15 | 77907378 | A | G | NM_032808 | c.871T>C:p.F291L | Missense | - | - | - | 12.3 | 1 | 0 |
| LINGO1 | chr15 | 77907582 | G | A | NM_032808 | c.667C>T:p.R223W | Missense | 0 | - | 0 | 32 | 0 | 1 |
| LINGO1 | chr15 | 77907665 | T | C | NM_032808 | c.584A>G:p.Q195R | Missense | - | - | - | 12.15 | 1 | 0 |
| LINGO1 | chr15 | 77907979 | C | G | NM_032808 | c.270G>C:p.E90D | Missense | - | - | - | 0.778 | 0 | 2 |
| LINGO1 | chr15 | 77907984 | C | T | NM_032808 | c.265G>A:p.D89N | Missense | - | - | - | 20.7 | 1 | 0 |
| LINGO1 | chr15 | 77908130 | G | A | NM_032808 | c.119C>T:p.T40M | Missense | 0 | - | 0 | 6.351 | 0 | 1 |
| LINGO1 | chr15 | 77908215 | C | T | NM_032808 | c.34G>A:p.V12M | Missense | 0 | 0.0006 | 0.0016 | 14.78 | 2 | 0 |
| LINGO1 | chr15 | 77908223 | G | A | NM_032808 | c.26C>T:p.A9V | Missense | 0 | - | - | 17.35 | 1 | 0 |
| NGFR | chr17 | 47579431 | C | T | NM_002507 | c.73C>T:p.L25F | Missense | 0.0094 | 0.0081 | 0.0078 | 6.467 | 39 | 23 |
| NGFR | chr17 | 47583775 | G | A | NM_002507 | c.323G>A:p.R108H | Missense | 0 | 0 | 0 | 25.4 | 1 | 0 |
| NGFR | chr17 | 47584005 | G | A | NM_002507 | c.553G>A:p.D185N | Missense | 0.0012 | 0.0019 | 0.0003 | 24.1 | 2 | 0 |
| NGFR | chr17 | 47587917 | A | G | NM_002507 | c.712A>G:p.S238G | Missense | 0.0005 | 0.0006 | 0.0007 | 20.1 | 1 | 3 |
| NGFR | chr17 | 47587929 | G | A | NM_002507 | c.724G>A:p.V242M | Missense | 0 | 0 | - | 24.4 | 0 | 1 |
| NGFR | chr17 | 47587959 | A | C | NM_002507 | c.754A>C:p.I252L | Missense | - | - | - | 23 | 4 | 0 |
| NGFR | chr17 | 47588013 | A | G | NM_002507 | c.808A>G:p.I270V | Missense | 0.0004 | 0 | 0.0003 | 14.15 | 2 | 0 |
| NGFR | chr17 | 47589294 | C | T | NM_002507 | c.862C>T:p.R288W | Missense | 0 | - | 0 | 35 | 0 | 1 |
| NGFR | chr17 | 47589393 | A | G | NM_002507 | c.961A>G:p.T321A | Missense | - | - | - | 15.51 | 1 | 0 |
| NGFR | chr17 | 47590083 | C | A | NM_002507 | c.996C>A:p.D332E | Missense | - | - | - | 13.89 | 0 | 1 |
| NGFR | chr17 | 47590118 | G | A | NM_002507 | c.1031G>A:p.R344Q | Missense | 0 | - | - | 23.1 | 1 | 0 |
| NGFR | chr17 | 47590144 | G | A | NM_002507 | c.1057G>A:p.G353S | Missense | 5.82E-05 | 0 | 0 | 14.99 | 1 | 1 |
| NGFR | chr17 | 47590211 | C | G | NM_002507 | c.1124C>G:p.S375C | Missense | - | - | - | 21.9 | 1 | 0 |
| NGFR | chr17 | 47590307 | G | A | NM_002507 | c.1220G>A:p.R407H | Missense | 0.0011 | 0.0025 | 0.0009 | 34 | 4 | 3 |
| NGFR | chr17 | 47590321 | G | A | NM_002507 | c.1234G>A:p.D412N | Missense | 0.0002 | - | 0.0003 | 28.4 | 1 | 0 |
| NGFR | chr17 | 47590327 | G | A | NM_002507 | c.1240G>A:p.V414M | Missense | 0 | - | 0 | 26.7 | 1 | 2 |
| NGFR | chr17 | 47590364 | C | T | NM_002507 | c.1277C>T:p.P426L | Missense | 0.0001 | 0 | 0 | 26.2 | 1 | 2 |
| ROCK1 | chr18 | 18533608 | G | A | NM_005406 | c.3992C>T:p.T1331M | Missense | 0.0012 | 0.0006 | 0.0007 | 24.7 | 4 | 5 |
| ROCK1 | chr18 | 18533644 | T | A | NM_005406 | c.3956A>T:p.N1319I | Missense | 0 | - | 0 | 22.8 | 0 | 1 |
| ROCK1 | chr18 | 18534807 | A | G | NM_005406 | c.3790T>C:p.C1264R | Missense | - | 0 | - | 28.1 | 5 | 2 |
| ROCK1 | chr18 | 18534812 | C | T | NM_005406 | c.3785G>A:p.R1262Q | Missense | - | 0 | - | 24.4 | 5 | 2 |
| ROCK1 | chr18 | 18534997 | A | C | NM_005406 | c.3600T>G:p.Y1200X | Stopgain | 0 | - | - | 44 | 1 | 0 |
| ROCK1 | chr18 | 18546925 | C | T | NM_005406 | c.3305G>A:p.S1102N | Missense | - | - | - | 33 | 0 | 1 |
| ROCK1 | chr18 | 18546932 | A | T | NM_005406 | c.3298T>A:p.S1100T | Missense | - | - | - | 15.92 | 1 | 0 |
| ROCK1 | chr18 | 18547762 | A | G | NM_005406 | c.3143T>C:p.F1048S | Missense | - | - | - | 24.5 | 0 | 1 |
| ROCK1 | chr18 | 18559925 | A | G | NM_005406 | c.2600T>C:p.I867T | Missense | - | - | - | 21.8 | 1 | 0 |
| ROCK1 | chr18 | 18564483 | G | C | NM_005406 | c.2318C>G:p.T773S | Missense | 5.90E-05 | 0.0006 | 0 | 12.92 | 0 | 1 |
| ROCK1 | chr18 | 18571149 | C | T | NM_005406 | c.2131G>A:p.V711M | Missense | - | 0.0006 | - | 32 | 0 | 1 |
| ROCK1 | chr18 | 18571191 | G | A | NM_005406 | c.2089C>T:p.R697C | Missense | 5.80E-05 | - | - | 34 | 0 | 2 |
| ROCK1 | chr18 | 18572801 | G | T | NM_005406 | c.1983C>A:p.H661Q | Missense | - | - | - | 11.14 | 1 | 0 |
| ROCK1 | chr18 | 18572842 | C | G | NM_005406 | c.1942G>C:p.V648L | Missense | - | - | - | 17.96 | 0 | 1 |
| ROCK1 | chr18 | 18608740 | C | T | NM_005406 | c.1208G>A:p.R403H | Missense | 0 | 0 | 0 | 22.9 | 1 | 0 |
| ROCK1 | chr18 | 18619444 | G | A | NM_005406 | c.1040C>T:p.T347M | Missense | 0 | 0 | 0 | 27.6 | 0 | 1 |
| ROCK1 | chr18 | 18619454 | C | T | NM_005406 | c.1030G>A:p.A344T | Missense | - | - | - | 18.53 | 0 | 1 |
| ROCK1 | chr18 | 18622059 | T | C | NM_005406 | c.958A>G:p.R320G | Missense | - | - | - | 28 | 0 | 1 |
| ROCK1 | chr18 | 18622083 | G | C | NM_005406 | c.934C>G:p.L312V | Missense | - | - | - | 22.9 | 1 | 0 |
| ROCK1 | chr18 | 18622088 | T | C | NM_005406 | c.929A>G:p.K310R | Missense | 0.0002 | - | 0.0003 | 21.6 | 2 | 0 |
| ROCK1 | chr18 | 18625407 | C | T | NM_005406 | c.436G>A:p.D146N | Missense | - | - | - | 27.1 | 0 | 1 |
| ROCK1 | chr18 | 18690825 | T | C | NM_005406 | c.47A>G:p.N16S | Missense | 5.80E-05 | - | 0 | 5.116 | 1 | 0 |
| S1PR2 | chr19 | 10334639 | G | A | NM_004230 | c.943C>T:p.R315W | Missense | 0.0013 | 0.0006 | 0.0012 | 24.2 | 2 | 0 |
| S1PR2 | chr19 | 10334663 | T | A | NM_004230 | c.919A>T:p.R307W | Missense | 0.0042 | 0.0075 | 0.0033 | 19.87 | 16 | 6 |
| S1PR2 | chr19 | 10334677 | G | A | NM_004230 | c.905C>T:p.P302L | Missense | 0 | - | 0 | 12.45 | 1 | 1 |
| S1PR2 | chr19 | 10334725 | A | G | NM_004230 | c.857T>C:p.V286A | Missense | 0.0021 | 0.0019 | 0.0025 | 26 | 3 | 5 |
| S1PR2 | chr19 | 10334807 | A | G | NM_004230 | c.775T>C:p.C259R | Missense | - | - | - | 24 | 1 | 0 |
| S1PR2 | chr19 | 10334855 | T | C | NM_004230 | c.727A>G:p.I243V | Missense | - | - | - | 14.59 | 0 | 1 |
| S1PR2 | chr19 | 10334939 | C | T | NM_004230 | c.643G>A:p.V215M | Missense | 0 | - | 0 | 24.6 | 1 | 0 |
| S1PR2 | chr19 | 10334954 | C | A | NM_004230 | c.628G>T:p.V210L | Missense | - | - | - | 9.702 | 0 | 1 |
| S1PR2 | chr19 | 10334954 | C | T | NM_004230 | c.628G>A:p.V210M | Missense | 0 | - | 0 | 23 | 1 | 0 |
| S1PR2 | chr19 | 10335280 | G | A | NM_004230 | c.302C>T:p.T101M | Missense | 0.0002 | - | 0.0003 | 24.4 | 1 | 0 |
| S1PR2 | chr19 | 10335403 | C | T | NM_004230 | c.179G>A:p.R60Q | Missense | 0.0028 | 0.0037 | 0.0027 | 27.5 | 15 | 7 |
| S1PR2 | chr19 | 10335552 | G | T | NM_004230 | c.30C>A:p.N10K | Missense | 0.0006 | 0 | 0.0006 | 14.19 | 2 | 1 |
| S1PR2 | chr19 | 10335566 | A | G | NM_004230 | c.16T>C:p.S6P | Missense | - | - | - | 24.6 | 1 | 1 |
| LILRB1 | chr19 | 55142477 | C | T |  |  | Splicing | 0 | - | 0 | 2.753 | 1 | 0 |
| LILRB1 | chr19 | 55142739 | A | T | NM_001081637 | c.52A>T:p.R18W | Missense | 5.80E-05 | - | 0 | - | 1 | 0 |
| LILRB1 | chr19 | 55142968 | A | G | NM_001081637 | c.88A>G:p.T30A | Missense | - | - | - | 11.16 | 0 | 1 |
| LILRB1 | chr19 | 55143034 | G | A | NM_001081637 | c.154G>A:p.G52S | Missense | 0 | 0 | 0 | 0.002 | 0 | 1 |
| LILRB1 | chr19 | 55143061 | T | C | NM_001081637 | c.181T>C:p.Y61H | Missense | - | - | - | 0.002 | 0 | 1 |
| LILRB1 | chr19 | 55143179 | A | G | NM_001081637 | c.299A>G:p.Y100C | Missense | 0 | 0 | 0 | 9.334 | 1 | 0 |
| LILRB1 | chr19 | 55143182 | G | T | NM_001081637 | c.302G>T:p.G101V | Missense | 0 | - | - | 0.001 | 1 | 0 |
| LILRB1 | chr19 | 55143199 | C | T | NM_001081637 | c.319C>T:p.R107C | Missense | 0.0001 | 0 | 0.0002 | 22.7 | 1 | 2 |
| LILRB1 | chr19 | 55143515 | A | C | NM_001081637 | c.488A>C:p.E163A | Missense | 0 | 0 | 0 | 0.314 | 0 | 1 |
| LILRB1 | chr19 | 55143519 | C | A | NM_001081637 | c.492C>A:p.H164Q | Missense | 5.80E-05 | - | - | 0.734 | 1 | 0 |
| LILRB1 | chr19 | 55143523 | C | G | NM_001081637 | c.496C>G:p.Q166E | Missense | 0.0003 | 0.0006 | 0.0003 | 0.003 | 1 | 0 |
| LILRB1 | chr19 | 55143551 | G | A | NM_001081637 | c.524G>A:p.R175H | Missense | 0.0002 | 0.0012 | 0.0002 | 0.007 | 1 | 2 |
| LILRB1 | chr19 | 55143596 | G | A | NM_001081637 | c.569G>A:p.S190N | Missense | 0 | 0 | 0 | 0.077 | 0 | 1 |
| LILRB1 | chr19 | 55143608 | G | C | NM_001081637 | c.581G>C:p.W194S | Missense | 0 | 0 | 0 | 0.051 | 0 | 1 |
| LILRB1 | chr19 | 55143610 | T | C | NM_001081637 | c.583T>C:p.Y195H | Missense | 0 | 0 | 0 | 7.367 | 0 | 1 |
| LILRB1 | chr19 | 55143632 | C | T | NM_001081637 | c.605C>T:p.S202L | Missense | 0.0048 | 0.0043 | 0.005 | 2.983 | 18 | 5 |
| LILRB1 | chr19 | 55143683 | T | G | NM_001081637 | c.656T>G:p.V219G | Missense | - | - | - | 13.17 | 1 | 0 |
| LILRB1 | chr19 | 55143689 | G | A | NM_001081637 | c.661+1G>A | Splicing | - | - | - | 13.28 | 0 | 1 |
| LILRB1 | chr19 | 55144039 | A | T | NM_001081637 | c.786A>T:p.E262D | Missense | - | - | - | 1.037 | 1 | 0 |
| LILRB1 | chr19 | 55144064 | G | A | NM_001081637 | c.811G>A:p.A271T | Missense | 0 | 0 | 0 | 0.05 | 1 | 0 |
| LILRB1 | chr19 | 55144083 | T | C | NM_001081637 | c.830T>C:p.L277P | Missense | 0 | 0 | 0 | 8.2 | 1 | 0 |
| LILRB1 | chr19 | 55144100 | A | G | NM_001081637 | c.847A>G:p.T283A | Missense | 0.0003 | 0 | 0.0001 | 0.001 | 1 | 3 |
| LILRB1 | chr19 | 55144117 | C | - | NM_001081637 | c.864delC:p.R289Afs*28 | Frameshift deletion | 0 | - | 0 | - | 1 | 0 |
| LILRB1 | chr19 | 55144125 | A | G | NM_001081637 | c.872A>G:p.Y291C | Missense | 0.0003 | 0.0012 | 0.0005 | 6.152 | 1 | 1 |
| LILRB1 | chr19 | 55144130 | G | A | NM_001081637 | c.877G>A:p.G293S | Missense | - | - | - | 23 | 0 | 1 |
| LILRB1 | chr19 | 55144145 | T | C | NM_001081637 | c.892T>C:p.Y298H | Missense | 5.80E-05 | - | 0.0001 | 22.5 | 1 | 0 |
| LILRB1 | chr19 | 55144154 | C | T | NM_001081637 | c.901C>T:p.H301Y | Missense | 0.0003 | 0 | 0.0002 | 0.017 | 1 | 0 |
| LILRB1 | chr19 | 55144159 | C | A | NM_001081637 | c.906C>A:p.N302K | Missense | 0 | - | 0 | 2.474 | 0 | 1 |
| LILRB1 | chr19 | 55144174 | G | C | NM_001081637 | c.921G>C:p.W307C | Missense | 5.80E-05 | - | 0.0001 | 16.36 | 2 | 0 |
| LILRB1 | chr19 | 55144488 | C | T | NM_001081637 | c.980C>T:p.S327F | Missense | 0 | - | - | 0.007 | 1 | 0 |
| LILRB1 | chr19 | 55144501 | G | T | NM_001081637 | c.993G>T:p.Q331H | Missense | 0.0026 | 0.0037 | 0.0022 | 0.001 | 5 | 2 |
| LILRB1 | chr19 | 55144532 | G | A | NM_001081637 | c.1024G>A:p.V342M | Missense | 5.80E-05 | - | 0.0001 | 23.3 | 0 | 4 |
| LILRB1 | chr19 | 55144611 | G | A | NM_001081637 | c.1103G>A:p.R368H | Missense | 0.0003 | 0 | 0.0006 | 0.064 | 0 | 1 |
| LILRB1 | chr19 | 55144623 | C | T | NM_001081637 | c.1115C>T:p.T372M | Missense | 0.0003 | 0 | 0.0001 | 0.004 | 1 | 2 |
| LILRB1 | chr19 | 55144670 | A | G | NM_001081637 | c.1162A>G:p.T388A | Missense | - | - | - | 13.06 | 1 | 0 |
| LILRB1 | chr19 | 55144710 | A | T | NM_001081637 | c.1202A>T:p.Q401L | Missense | 0.0003 | 0 | 0.0001 | 0.001 | 2 | 2 |
| LILRB1 | chr19 | 55144711 | G | C | NM_001081637 | c.1203G>C:p.Q401H | Missense | 0.0003 | 0 | 0.0001 | 0.001 | 2 | 2 |
| LILRB1 | chr19 | 55144713 | G | A | NM_001081637 | c.1205G>A:p.S402N | Missense | 5.80E-05 | - | 0 | 0.135 | 1 | 0 |
| LILRB1 | chr19 | 55144718 | A | G | NM_001081637 | c.1210A>G:p.K404E | Missense | 5.80E-05 | 0 | 0 | 0.033 | 1 | 0 |
| LILRB1 | chr19 | 55144720 | A | C | NM_001081637 | c.1212A>C:p.K404N | Missense | 0.0003 | 0 | 0.0001 | 0.003 | 2 | 2 |
| LILRB1 | chr19 | 55144730 | C | G | NM_001081637 | c.1222C>G:p.L408V | Missense | - | - | - | 16.85 | 1 | 0 |
| LILRB1 | chr19 | 55144733 | A | T | NM_001081637 | c.1225A>T:p.T409S | Missense | 0.0013 | 0.0012 | 0.0015 | 0.001 | 4 | 2 |
| LILRB1 | chr19 | 55144734 | C | T | NM_001081637 | c.1226C>T:p.T409I | Missense | - | - | - | 13.38 | 0 | 1 |
| LILRB1 | chr19 | 55144736 | C | T | NM_001081637 | c.1228C>T:p.H410Y | Missense | 5.80E-05 | - | 0.0001 | 0.001 | 1 | 0 |
| LILRB1 | chr19 | 55144737 | A | T | NM_001081637 | c.1229A>T:p.H410L | Missense | 5.80E-05 | - | 0.0001 | 0.001 | 1 | 0 |
| LILRB1 | chr19 | 55144754 | G | A | NM_001081637 | c.1246G>A:p.E416K | Missense | - | - | - | 17 | 0 | 1 |
| LILRB1 | chr19 | 55145092 | C | T | NM_001081637 | c.1265C>T:p.P422L | Missense | 0.0002 | 0 | 0.0002 | 13.89 | 1 | 0 |
| LILRB1 | chr19 | 55145113 | C | T | NM_001081637 | c.1286C>T:p.P429L | Missense | 0.0002 | 0 | 0.0002 | 17.04 | 2 | 0 |
| LILRB1 | chr19 | 55145139 | G | C | NM_001081637 | c.1312G>C:p.A438P | Missense | - | - | - | 0.069 | 1 | 0 |
| LILRB1 | chr19 | 55146148 | CTC | - | NM_001081637 | c.1420_1422del:p.L480del | NonFrameshift deletion | 0.0027 | 0.0025 | 0.0069 | - | 1 | 3 |
| LILRB1 | chr19 | 55146190 | C | T | NM_001081637 | c.1462C>T:p.R488C | Missense | 5.80E-05 | 0 | 0 | 11.02 | 0 | 1 |
| LILRB1 | chr19 | 55146191 | G | A | NM_001081637 | c.1463G>A:p.R488H | Missense | 0 | - | 0 | 0.673 | 0 | 1 |
| LILRB1 | chr19 | 55146627 | G | A | NM_001081637 | c.1559G>A:p.W520X | Stopgain | 0.0002 | - | 0.0002 | 26.7 | 1 | 0 |
| LILRB1 | chr19 | 55146715 | G | A | NM_001081637 | c.1568G>A:p.S523N | Missense | - | - | - | 0.178 | 2 | 0 |
| LILRB1 | chr19 | 55146721 | C | G | NM_001081637 | c.1574C>G:p.A525G | Missense | - | - | - | 1.709 | 1 | 0 |
| LILRB1 | chr19 | 55147023 | A | G | NM_001081637 | c.1616A>G:p.H539R | Missense | 0.0003 | 0.0012 | 0.0003 | 2.591 | 1 | 1 |
| LILRB1 | chr19 | 55147957 | G | A | NM_001081637 | c.1666G>A:p.D556N | Missense | 0 | - | 0 | 6.522 | 1 | 0 |
| LILRB1 | chr19 | 55147971 | G | C | NM_001081637 | c.1680G>C:p.Q560H | Missense | 0 | 0.0006 | 0 | 0.009 | 5 | 0 |
| LILRB1 | chr19 | 55147987 | G | A | NM_001081637 | c.1696G>A:p.E566K | Missense | 0.001 | 0.002 | 0.0013 | 7.117 | 0 | 2 |
| LILRB1 | chr19 | 55148014 | G | T | NM_001081637 | c.1723G>T:p.E575X | Stopgain | 0 | - | 0 | 35 | 0 | 1 |
| LILRB1 | chr19 | 55148075 | C | T | NM_001081637 | c.1784C>T:p.A595V | Missense | 0 | 0 | 0 | 8.389 | 3 | 0 |
| LILRB1 | chr19 | 55148103 | G | - | NM_001081637 | c.1812delG:p.A605Lfs*9 | Frameshift deletion | 0.0001 | 0 | 0 | - | 2 | 0 |
| LILRB1 | chr19 | 55148105 | T | A | NM_001081637 | c.1812+2T>A | Splicing | 0.0001 | 0 | 0 | 0.081 | 2 | 0 |
| LILRB1 | chr19 | 55148201 | C | T | NM_001081637 | c.1831C>T:p.P611S | Missense | 0 | 0.0006 | 0 | 3.686 | 1 | 0 |
| LILRB1 | chr19 | 55148295 | T | A | NM_001081637 | c.1925T>A:p.V642E | Missense | 0.0018 | 0.0012 | 0.0042 | 0.002 | 1 | 0 |
| RTN4R | chr22 | 20229238 | C | A | NM_023004 | c.1418G>T:p.C473F | Missense | - | - | - | 24 | 1 | 0 |
| RTN4R | chr22 | 20229248 | G | A | NM_023004 | c.1408C>T:p.L470F | Missense | - | - | - | 10.06 | 1 | 0 |
| RTN4R | chr22 | 20229281 | G | C | NM_023004 | c.1375C>G:p.P459A | Missense | 9.68E-05 | - | - | 1.134 | 1 | 1 |
| RTN4R | chr22 | 20229302 | T | C | NM_023004 | c.1354A>G:p.S452G | Missense | - | - | - | 0.008 | 1 | 0 |
| RTN4R | chr22 | 20229395 | G | A | NM_023004 | c.1261C>T:p.R421C | Missense | 0 | - | - | 25.5 | 1 | 0 |
| RTN4R | chr22 | 20229455 | C | T | NM_023004 | c.1201G>A:p.E401K | Missense | 0 | - | 0 | 21 | 1 | 0 |
| RTN4R | chr22 | 20229526 | C | T | NM_023004 | c.1130G>A:p.R377Q | Missense | 0.0008 | 0 | 0.001 | 23 | 1 | 1 |
| RTN4R | chr22 | 20229569 | C | T | NM_023004 | c.1087G>A:p.V363M | Missense | 0.0032 | 0.0049 | 0.0027 | 10.12 | 15 | 10 |
| RTN4R | chr22 | 20229625 | G | T | NM_023004 | c.1031C>A:p.A344D | Missense | 0.0003 | - | 0.0001 | 13.59 | 2 | 1 |
| RTN4R | chr22 | 20229628 | T | G | NM_023004 | c.1028A>C:p.K343T | Missense | - | - | - | 23.8 | 2 | 0 |
| RTN4R | chr22 | 20229634 | G | C | NM_023004 | c.1022C>G:p.A341G | Missense | - | - | - | 0.242 | 0 | 1 |
| RTN4R | chr22 | 20229680 | C | T | NM_023004 | c.976G>A:p.D326N | Missense | 0.0001 | - | 0.0003 | 0.612 | 2 | 1 |
| RTN4R | chr22 | 20229781 | C | T | NM_023004 | c.875G>A:p.R292H | Missense | 6.69E-05 | 0 | - | 11.93 | 0 | 1 |
| RTN4R | chr22 | 20229782 | G | A | NM_023004 | c.874C>T:p.R292C | Missense | 0 | - | 0 | 15.99 | 1 | 0 |
| RTN4R | chr22 | 20229881 | C | T | NM_023004 | c.775G>A:p.D259N | Missense | 0.0005 | - | 0.0005 | 13.08 | 1 | 0 |
| RTN4R | chr22 | 20229911 | G | T | NM_023004 | c.745C>A:p.L249M | Missense | - | - | - | 22.2 | 1 | 0 |
| RTN4R | chr22 | 20230000 | G | A | NM_023004 | c.656C>T:p.P219L | Missense | 5.80E-05 | - | 0.0001 | 20.2 | 1 | 0 |
| RTN4R | chr22 | 20230069 | C | T | NM_023004 | c.587G>A:p.R196H | Missense | 0 | 0 | 0 | 17.98 | 1 | 0 |
| RTN4R | chr22 | 20230090 | C | T | NM_023004 | c.566G>A:p.R189H | Missense | - | - | - | 21.9 | 1 | 0 |
| RTN4R | chr22 | 20230133 | G | A | NM_023004 | c.523C>T:p.R175C | Missense | 0.0003 | - | 0.0005 | 25.6 | 2 | 1 |
| RTN4R | chr22 | 20230140 | G | C | NM_023004 | c.516C>G:p.D172E | Missense | 0.0015 | 0.0006 | 0.0019 | 17.78 | 4 | 1 |
| RTN4R | chr22 | 20230163 | C | T | NM_023004 | c.493G>A:p.A165T | Missense | 0 | 0 | 0 | 10.71 | 1 | 0 |
| RTN4R | chr22 | 20230202 | C | T | NM_023004 | c.454G>A:p.G152S | Missense | - | - | - | 27.1 | 0 | 1 |
| RTN4R | chr22 | 20230205 | G | A | NM_023004 | c.451C>T:p.R151C | Missense | 0 | - | 0 | 28.4 | 1 | 0 |
| RTN4R | chr22 | 20230267 | C | A | NM_023004 | c.389G>T:p.G130V | Missense | 0.0004 | - | 0.0002 | 13.3 | 1 | 4 |
| RTN4R | chr22 | 20230300 | C | G | NM_023004 | c.356G>C:p.R119P | Missense | - | - | - | 20.6 | 1 | 0 |
| RTN4R | chr22 | 20230363 | G | A | NM_023004 | c.293C>T:p.A98V | Missense | 0.0002 | 0.0006 | 0.0002 | 18.96 | 1 | 1 |
| RTN4R | chr22 | 20230486 | G | A | NM_023004 | c.170C>T:p.A57V | Missense | - | - | - | 0.075 | 0 | 1 |
| RTN4R | chr22 | 20230499 | C | T | NM_023004 | c.157G>A:p.V53M | Missense | 0.0001 | 0 | 0.0001 | 0.033 | 1 | 2 |
| RTN4R | chr22 | 20230541 | C | T | NM_023004 | c.115G>A:p.V39M | Missense | - | - | - | 11.25 | 1 | 2 |
| RTN4R | chr22 | 20230568 | C | A | NM_023004 | c.88G>T:p.A30S | Missense | 0 | - | - | 2.538 | 3 | 0 |
| RTN4R | chr22 | 20230571 | C | G | NM_023004 | c.85G>C:p.G29R | Missense | - | - | - | 5.78 | 0 | 1 |
| RTN4R | chr22 | 20230598 | C | A | NM_023004 | c.58G>T:p.A20S | Missense | - | - | - | 17.61 | 0 | 2 |
| RTN4R | chr22 | 20230627 | C | T | NM_023004 | c.29G>A:p.R10Q | Missense | 0 | 0 | - | 4.91 | 2 | 0 |

**Reference**

Bernstein, I. L., Bernstein, D. I., Balakrishnan, K., & Korbee, L. (1989). Infertility treated with donor specific lymphocytes in recurrent idiopathic spontaneous abortion. *Transplant Proc, 21*(1 Pt 1), 565.

Guo, J. F., Zhang, L., Li, K., Mei, J. P., Xue, J., Chen, J., et al. (2018). Coding mutations in NUS1 contribute to Parkinson's disease. *Proc Natl Acad Sci U S A, 115*(45), 11567-11572. doi:10.1073/pnas.1809969115

Li, J., Shi, L., Zhang, K., Zhang, Y., Hu, S., Zhao, T., et al. (2018). VarCards: an integrated genetic and clinical database for coding variants in the human genome. *Nucleic Acids Res, 46*(D1), D1039-d1048. doi:10.1093/nar/gkx1039

Pan, H. X., Zhao, Y. W., Mei, J. P., Fang, Z. H., Wang, Y., Zhou, X., et al. (2020). GCH1 variants contribute to the risk and earlier age-at-onset of Parkinson's disease: a two-cohort case-control study. *Transl Neurodegener, 9*(1), 31. doi:10.1186/s40035-020-00212-3

Schunk, K., Weber, W., Strunk, H., Regentrop, H., Thelen, R., & Schild, H. (1989). [Traumatology and diagnosis of scaphoid fracture]. *Radiologe, 29*(2), 61-67.

Yang, H., & Wang, K. (2015). Genomic variant annotation and prioritization with ANNOVAR and wANNOVAR. *Nat Protoc, 10*(10), 1556-1566. doi:10.1038/nprot.2015.105

Zhao, Y., Qin, L., Pan, H., Liu, Z., Jiang, L., He, Y., et al. (2020). The role of genetics in Parkinson's disease: a large cohort study in Chinese mainland population. *Brain, 143*(7), 2220-2234. doi:10.1093/brain/awaa167
