# Supplementary figures and images for: Genetic Analysis of Neurite Outgrowth Inhibitor‐Associated Genes in Parkinson's Disease: A Cross‐Sectional Cohort Study
Source: CNS Neurosci Ther. 2024 Oct 2;30(10):e70070. doi: 10.1111/cns.70070 (PMC11445604; doi:10.1111/cns.70070)

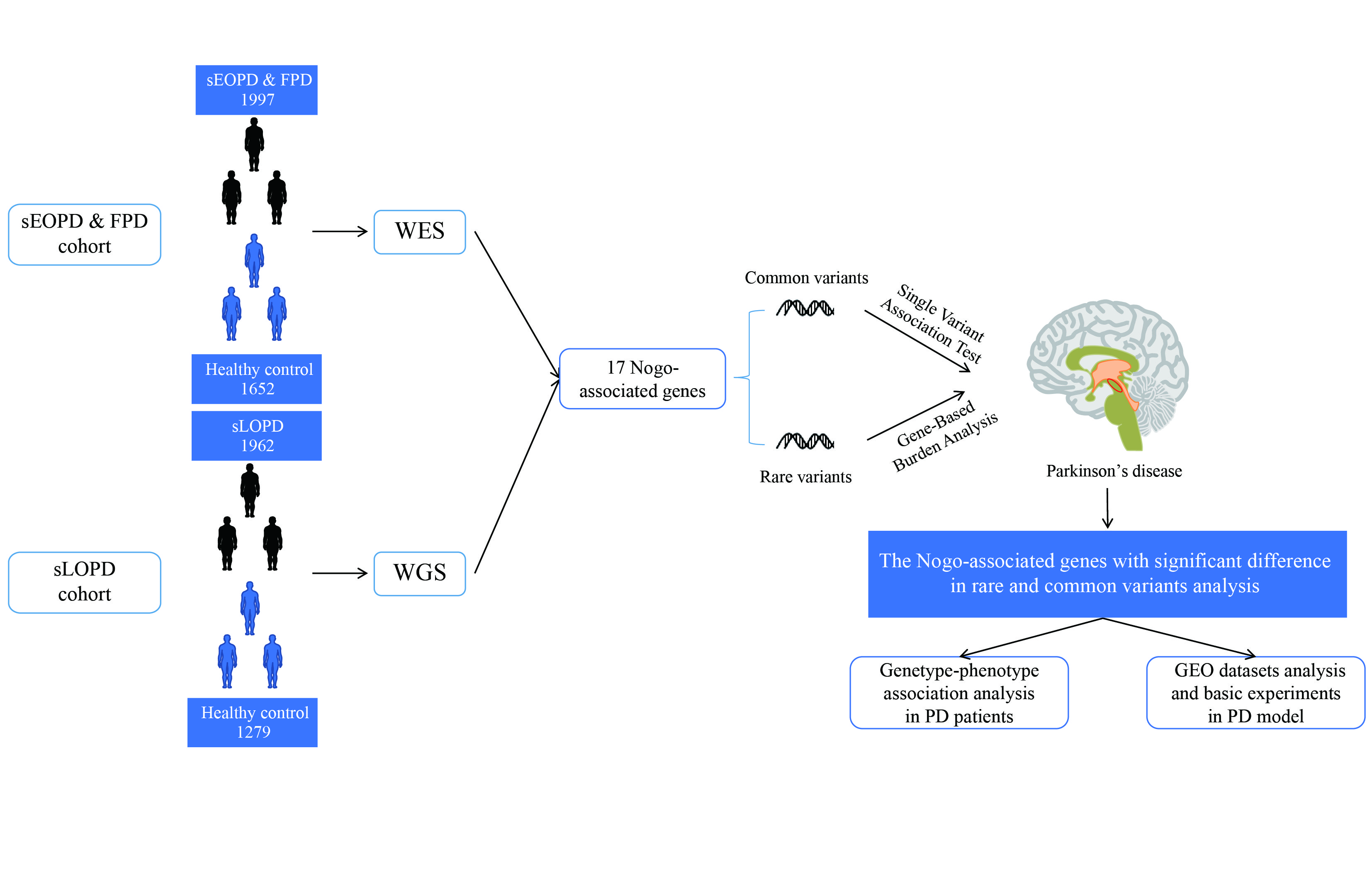

Supplement: Supplementary file 2 — Figure S1. Flowchart of this study. Subjects in the sEOPD & FPD cohorts underwent Whole exome sequence (WES) and Subjects in the sLOPD cohort underwent whole genome sequence (WGS). Based on above two cohorts, we aimed to systematically screen and identify Nogo‐associated genes. Gene‐based burden analysis (SKAT‐O and Fisher tests) were used to identify the relationship between rare variants of Nogo‐associated genes and PD risk. Single variant association test (Logistic regression) were used to identify the relationship between common variants of Nogo‐associated genes and PD risk. Next, genotype–phenotype association analysis in our study was complemented by Linear or Logistic regression, followed by GEO datasets analysis and basic experiments in PD model. [file CNS-30-e70070-s003.jpg]

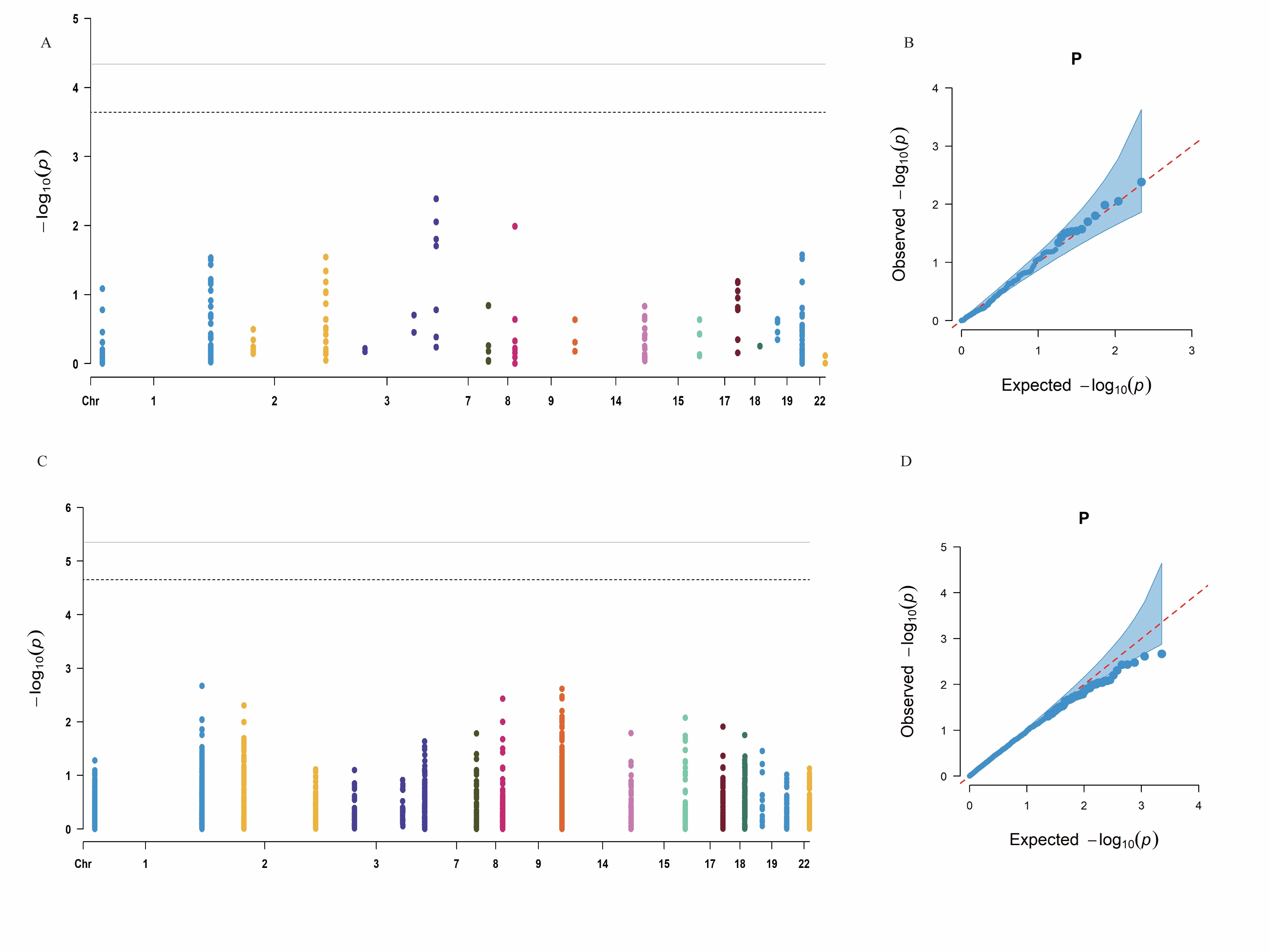

Supplement: Supplementary file 3 — Figure S2. Summary of single common variants in sEOPD & FPD cohort and sLOPD cohort. A, Manhattan plot of all common variants in the sEOPD & FPD cohort; B, QQ graph of all common variants in the sEOPD & FPD cohort; C, Manhattan plot of all common variants in the sLOPD cohort; D, QQ graph of all common variants in the sLOPD cohort. [file CNS-30-e70070-s001.jpg]
